# Pharmacodynamic study of radium-223 in men with bone metastatic castration resistant prostate cancer

**DOI:** 10.1371/journal.pone.0216934

**Published:** 2019-05-28

**Authors:** Andrew J. Armstrong, Santosh Gupta, Patrick Healy, Gabor Kemeny, Beth Leith, Michael R. Zalutsky, Charles Spritzer, Catrin Davies, Colin Rothwell, Kathryn Ware, Jason A. Somarelli, Kris Wood, Thomas Ribar, Paraskevi Giannakakou, Jiaren Zhang, Drew Gerber, Monika Anand, Wen-Chi Foo, Susan Halabi, Simon G. Gregory, Daniel J. George

**Affiliations:** 1 Department of Medicine, Division of Medical Oncology, Duke Cancer Institute, Duke University, Durham, NC, United States of America; 2 Department of Pharmacology and Cancer Biology, Duke University, Durham, NC, United States of America; 3 Duke Prostate and Urologic Cancer Center, Duke Cancer Institute, Durham, NC, United States of America; 4 Duke Molecular Physiology Institute, Duke University, Durham, NC, United States of America; 5 Department of Biostatistics, Duke University, Durham, NC, United States of America; 6 Department of Radiology, Duke University, Durham, NC, United States of America; 7 Weill Cornell Medical College, New York, NY, United States of America; 8 Duke Department of Pathology, Duke University, Durham, NC, United States of America; Hospital de Santa Maria, PORTUGAL

## Abstract

**Background:**

Radium-223 is a targeted alpha-particle therapy that improves survival in men with metastatic castration resistant prostate cancer (mCRPC), particularly in men with elevated serum levels of bone alkaline phosphatase (B-ALP). We hypothesized that osteomimicry, a form of epithelial plasticity leading to an osteoblastic phenotype, may contribute to intralesional deposition of radium-223 and subsequent irradiation of the tumor microenvironment.

**Methods:**

We conducted a pharmacodynamic study (NCT02204943) of radium-223 in men with bone mCRPC. Prior to and three and six months after radium-223 treatment initiation, we collected CTCs and metastatic biopsies for phenotypic characterization and CTC genomic analysis. The primary objective was to describe the impact of radium-223 on the prevalence of CTC B-ALP over time. We measured radium-223 decay products in tumor and surrounding normal bone during treatment. We validated genomic findings in a separate independent study of men with bone metastatic mCRPC (n = 45) and publicly accessible data of metastatic CRPC tissues.

**Results:**

We enrolled 20 men with symptomatic bone predominant mCRPC and treated with radium-223. We observed greater radium-223 radioactivity levels in metastatic bone tumor containing biopsies compared with adjacent normal bone. We found evidence of persistent Cellsearch CTCs and B-ALP (+) CTCs in the majority of men over time during radium-223 therapy despite serum B-ALP normalization. We identified genomic gains in osteoblast mimicry genes including gains of ALPL, osteopontin, SPARC, OB-cadherin and loss of RUNX2, and validated genomic alterations or increased expression at the DNA and RNA level in an independent cohort of 45 men with bone-metastatic CRPC and in 150 metastatic biopsies from men with mCRPC.

**Conclusions:**

Osteomimicry may contribute in part to the uptake of radium-223 within bone metastases and may thereby enhance the therapeutic benefit of this bone targeting radiotherapy.

## Introduction

Metastatic prostate cancer (PC) has a specific bone tropism, and the majority of men with lethal prostate cancer develop bone metastases, resulting in significant morbidity due to fracture, pain, and bone marrow failure[1–3]. The biological mechanisms underlying hematogenous spread to bone are incompletely understood, but several key pathways have been identified including osteomimicry, hematopoietic stem cell mimicry, and dysregulation of adhesion molecule expression favoring bone growth[4–7]. The majority of men with PC harbor osteoblastic bone metastases, resulting in an imbalance in pathologic bone formation over reabsorption. In patients, however, our poor understanding of the interaction between the bone microenvironment and disseminating PC cells limits our ability to target these processes.

Radium-223 is a bone targeting alpha-particle emitter, a targeted radiotherapy that can provide improved overall survival in men with bone-metastatic castration resistant PC (CRPC) and is now routinely used for men with symptomatic bone metastases from CRPC[8]. In a phase III randomized control trial, radium-223 reduced pathologic fractures and spinal cord compressions, while lowering serum markers of bone turnover such as levels of bone alkaline phosphatase, which correlated with improved survival[9–11]. However, the basis for the clinical activity and specific effects within the osteoblastic tumor microenvironment of mCRPC, has not been well studied.

Serum bone alkaline phosphatase (B-ALP, gene ALPL) has long been understood to be elevated in men with prostate cancer and bone metastases and was originally described by Huggins and Hodges to transiently rise and then fall after orchiectomy and clinical response to androgen deprivation therapy[12]. The ALPL enzyme is expressed by osteoblasts and is essential for bone matrix formation and the generation of free phosphates favoring bone development[13]. Recent data supports a key role, however, of ALPL in prostate cancer cells themselves through the regulation of epithelial plasticity, invasion, and resistance to cell death[14]. Osteomimicry, which is defined as the phenotypic acquisition of bone forming properties by cancer cells, has been observed in prostate cancer cells in vitro, and the expression of osteoblastic factors such as ALPL by PC cells is associated with these phenotypic changes in vitro (14). In patients, elevated expression of B-ALP correlates with worse outcomes[5, 14–17].

We hypothesized that osteomimicry and expression of osteoblastic factors by PC cells in bone metastases may lead to enhanced deposition and therapeutic benefit of radium-223. To address this question, we conducted a pharmacodynamic study of radium-223 in men with bone metastatic CRPC. Here we describe and validate genomic and phenotypic evidence supporting osteomimicry in circulating tumor cells (CTCs) from men undergoing radium-223 therapy, accompanied by enhanced radium-223 deposition in bone metastases.

## Methods

### Patient selection and clinical study design

We conducted a prospective investigator initiated translational study of radium-223 in men with bone metastases and progressive mCRPC. Eligibility for this study required histologic evidence of PC, >2 sites of bone metastases with at least one site amenable to bone biopsy, symptomatic disease as determined by the treating physician, prior therapy with either abiraterone or enzalutamide, and progressive disease despite ongoing androgen deprivation therapy as per PCWG2 guidelines[18]. For full details of eligibility, see clinicaltrials.gov NCT02204943 and the **Data Supplement**. All patients were treated at Duke University, whose IRB specifically approved this study undertwo IRB approved protocols Pro00053925 and Pro00056936. All patients provided written informed consent. For the validation cohort (PROPHECY study NCT02269982), men with bone metastatic mCRPC were included (n = 45, see **Data Supplement** for eligibility).

### Treatment

For the pharmacodynamic study (n = 20), radium-223 was administered per standard practice at dose of 55 kBq/kg every 4 weeks for up to 6 doses. Radium-223 was administered as a bolus intravenous (IV) injection (up to 1 minute). Concurrent therapy with ongoing androgen deprivation therapy, abiraterone or enzalutamide, prednisone, and denosumab or zoledronic acid was permitted. Standard-of-care assessments required baseline laboratory parameters including bone marrow, kidney and hepatic function, coagulation parameters, which were repeated on a monthly basis prior to the next radium-223 dose. Imaging with CT chest/abdomen/pelvis with and without contrast and Technetium-99 MDP bone scan was performed at baseline, after 3 months of therapy, and following completion of 6 doses of radium-223. Safety data were collected and reported using NCI Common Toxicity Criteria version 4.0. Progression was defined as radiographic progression on imaging (CT, bone scan) using PCWG2 criteria or symptomatic progression resulting in clinical deterioration as determined by the treating provider, or death, whichever came first. PSA levels were not used to define progression.

### Correlative studies

Research studies conducted included CT guided targeted optional bone biopsies in patients prior to radium-223 administration and these were repeated after 3 doses of radium-223 (day 15) and after 6 doses of radium-223. Circulating tumor cells (CTCs) were isolated and enumerated using the Cellsearch method[19] at baseline and day 1 of the 3^rd^ dose of radium-223, and at progression. CTCs were also collected in 7.5 mL EDTA tubes using our previously described negative selection method of red cell lysis and CD45 depletion followed by FACS sorting of CD45 negative cells. These CTCs were isolated for whole genome DNA copy number analysis using aCGH according to our prior published methods[20]. For whole genome copy number analysis of paired patient leukocytes (germline DNA) and CTCs, an Agilent 4X180K-comparative genomic hybridization (CGH) chip was used, and further analyzed using Agilent’s Cytogenomics software.

The expression of bone alkaline phosphatase (B-ALP) in CTCs was assessed using the Imagestream FACS/immunofluorescence analysis platform[21] following collection of CTCs in 7.5 mL whole blood in EDTA tubes. CTCs were isolated through red cell lysis using ammonium chloride and leukocytes depleted using magnetic beads specific to CD45 (RosetteSep CD45 Depletion Cocktail, StemCell Technologies). For immunofluorescent staining, the following reagents were used: anti-CD45 (Leinco, C1624 mouse monoclonal antibody, detected with Alexafluor 650 conjugated to anti-mouse IgG), DAPI, anti-EpCAM (ABD serotec, MCA1870G mouse monoclonal, detected with Alexafluor-555 conjugated to anti-mouse IgG), and anti-ALPL (B-ALP, ab108337 rabbit monoclonal antibody, detected with Alexa-488 conjugated to goat IgG anti-rabbit antibody). The Imagestream method was used to isolate single nucleated DAPI positive cells or clusters that lacked CD45 expression, and these cells were further characterized by immunofluorescence for the expression of EpCAM and B-ALP. Positive control cells for ALPL were HeLa cells, spiked into blood from healthy volunteers, and positive controls for EpCAM wereT47D breast cancer cells spiked into blood from healthy volunteers, while normal peripheral blood mononuclear cells (PBMCs) were used for positive CD45 controls. Negative controls for ALPL were T47D breast cancer cells and PBMCs, which did not express B-ALP (n = 10 healthy volunteers), and negative controls for EpCAM were HeLa cells and PBMCs (n = 10).

HeLa and LnCAP positive and negative control cells were cultured in X Medium (DMEM) supplemented with 10% fetal bovine serum (FBS) and 1% penicillin/streptomycin (pen-strep) at 37°C and 5% CO2 in a humidified incubator. Cell lines were obtained from the Duke University Cell Culture Facility, which performs routine mycoplasma testing and verifies cell identity by analysis of short tandem repeats. Cell lines were spiked into 7.5 mL of healthy volunteer blood collected in EDTA tubes under a Duke IRB approved clinical study, and the detection rate and gating thresholds for ALPL (HeLa cells as positive control, LnCAP cells as negative control) and EpCAM expression (LnCAP cells as positive controls, HeLa cells as negative controls) were established and applied for patient CTC biomarker assessments. Ten healthy volunteers contributed blood for these control spiking experiments. Leukocytes (peripheral blood mononuclear cells, or PBMCs) were also assessed from healthy volunteer blood for EpCAM and B-ALP expression.

RNA Sequencing was performed for the subset of patients enrolled in the PROPHECY validation study. CTCs were enriched following negative depletion using the RosetteSep Human CD45 depletion cocktail protocol. A matching leukocyte sample for each patient, obtained through FICOLL, was included as a control. CTC and leukocyte mRNA was used to prepare sequencing libraries using the SMART-Seq v4 Ultra Low Input RNA Kit (Clontech). Briefly, this template switching (Switching Mechanism at 5’ End of RNA Template) technology[22] was applied to amplify the extracted mRNA and provide direct cDNA synthesis for downstream Illumina RNA-Seq. Sample libraries were multiplexed and 50 base pair (bp) paired-end RNA-Seq was performed on Illumina HiSeq4000 platform. Trimmed RNA-Seq reads were aligned to human genome (version hg38) using STAR (version 2.5), and Fragments Per Kilobase of exon per Million mapped fragments (FPKM) was calculated using Cufflinks.

Freshly biopsied bone metastases were counted to assess the levels of radium-223 and decay products in bone[23]. Samples were processed using an automated dual-window sodium iodide gamma counter (Perkin Elmer Model 1480) and a single energy window was set to encompass emissions from radium-223 and its decay products. Biopsy samples were placed in sealed plastic tubes and counted after being sectioned into deep tumor contained targeted biopsies and superficial adjacent cortical bone biopsies. We compared activity levels in the biopsies with radium-223 samples of known activity (about 0.1 mCi), representing a known fraction of the injected dose administered to the patient; correction for room background radioactivity was applied. Because we anticipated that radioactivity levels in the biopsy samples would be low, each tube was counted for 10 minutes or until 1000 net counts were reached, which limited counting error to 3% counting error. Measurements were repeated in triplicate and results expressed as nCi per gram tissue and percent injected dose per gram tissue.

CTC cultures were grown using a negative selection method of CTC isolation in 7.5 mL EDTA whole blood, followed by red cell lysis and CD45 bead depletion of leukocytes. The CD45 negative fraction was then placed in culture F medium containing the Rho Kinase (ROCK) inhibitor Y-27632 and grown on irradiated mouse fibroblasts as per the Georgetown published method[24]. Cells were isolated and tested by aCGH for copy number analysis to confirm prostate cancer identity and compared with the matched patient CTCs. Expression of B-ALP, EpCAM, and CD45 of cultured CTCs was evaluated by immunofluorescence. We assessed the presence of human vs. mouse DNA using species specific primers (see **Data Supplement**).

### Statistical analysis plan

The primary endpoint of this study was to describe the prevalence of CTC B-ALP expression over time during radium-223 treatment, as a measure of prostate cancer osteomimicry. Exploratory endpoints included the detection of radium-223 derived radioactive emissions from bone metastases and the biodistribution of radium-223 in bone metastases as compared to adjacent normal bone, changes in CTC enumeration (Cellsearch) and serum ALPL levels over time, changes in CTC genomic alterations over time. Exploratory clinical endpoints included PSA declines from baseline during radium-223 therapy, progression free survival defined as the time from enrollment until clinical symptomatic progression, a skeletal related event, or radiographic progression as defined by PCWG2, and overall survival, defined as the time from enrollment until death. Skeletal related events were defined as a new pathologic fracture, the need for radiation to tumor site, or malignant spinal cord compression. PSA rises alone were not used to define progression. Date of death was determined directly from review of the patient’s medical records with source verification. These patients were all treated at a single institution and no patients were lost to follow up. Social security death indices and patient obituaries were used to verify dates of death with an administrative cutoff date of April 8, 2019.

As this was the first study to estimate the proportion of patients who over-express B-ALP in CTCs, as such the prevalence of patients who were expected to over-express B-ALP in the bone metastases was unknown. It was anticipated that at least 50% of enrolled men will over-express B-ALP in their CTCs and/or bone metastases. CTC B-ALP positivity was determined by the presence of at least one CTC expressing B-ALP, with a CTC defined as a CD45 negative, nucleated, intact cell. CTCs were also be defined by their EpCAM expression, and B-ALP expression also described within the EpCAM negative fraction of CTCs. With 20 patients enrolled in the study, the proportion of patients who will over-express alkaline phosphatase in the bone metastases (defined as any over-expression) can be estimated with a standard error of no greater than 11.2 percentage points and exact 95% confidence intervals based on the binomial distribution were computed. The expression levels of the biomarkers including CTC enumeration and RNA and protein biomarkers were summarized using descriptive statistics (means, SE, median, and inter-quartile ranges) at baseline and over time, with 95% confidence intervals around these estimates in both cases and healthy volunteer controls. CTC RNA expression was compared to normal leukocyte (FICOLL) RNA expression using paired t-tests under parametric conditions, two sided. We performed non-parametric methods including the Kruskal-Wallis test for the comparison of CTC B-ALP expression between patients and healthy volunteers. Waterfall plots for changes from baseline for all markers such as PSA and alkaline phosphatase at the indicated time point were used. The Spearman correlation coefficient and 95% confidence interval were calculated for each paired marker including the correlation of CTC B-ALP and serum ALP levels. The Kaplan-Meier product limit method was used to estimate the PFS and OS distributions in an exploratory manner.

## Results

### Patient characteristics

**Fig 1:**
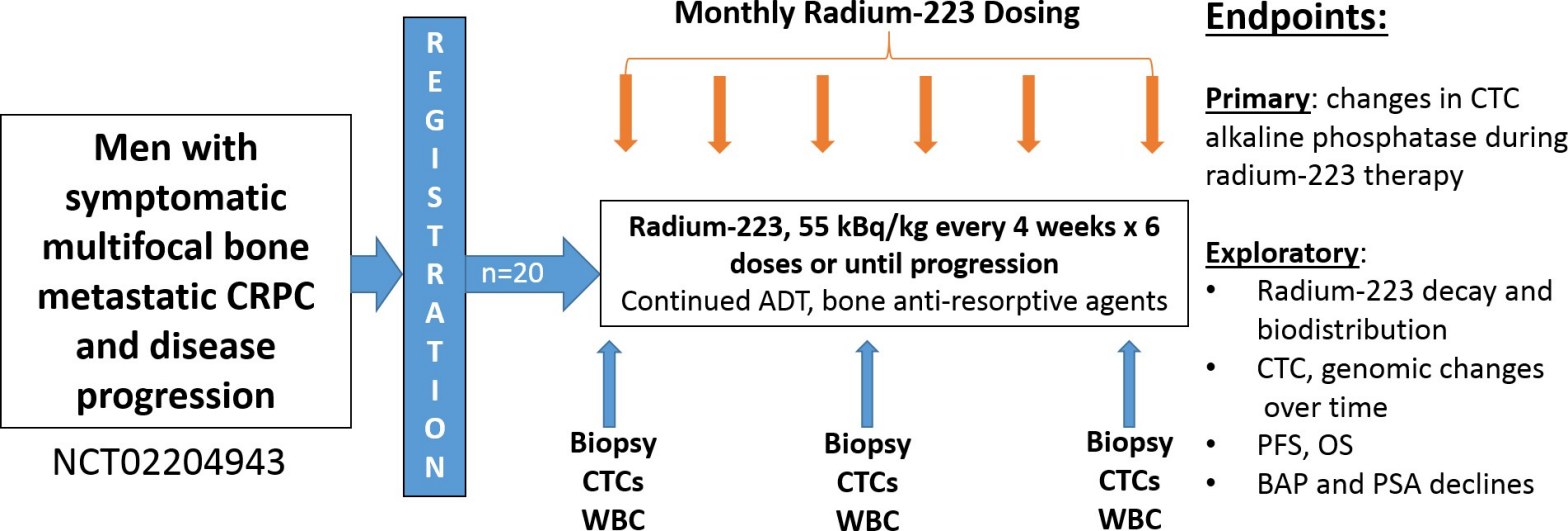
Schema for patient flow for the radium-223 pharmacodynamic study.

**Fig 2:**
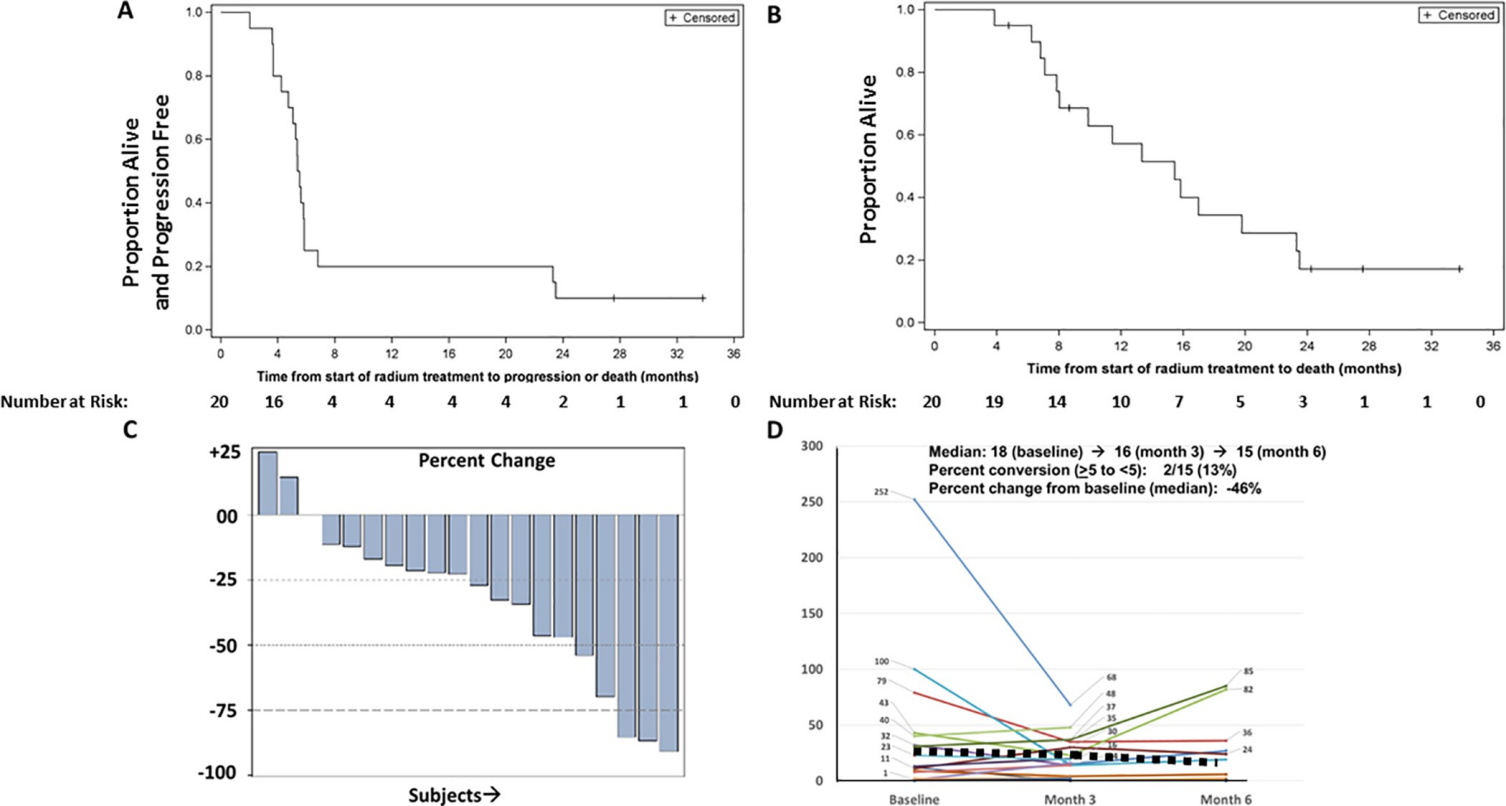
**A.** Kaplan-Meier plot of progression free survival of patients treated with radium-223. **B**. Kaplan-Meier plot of overall survival of patients treated with radium-223. **C**. Waterfall plot of serum bone alkaline phosphatase declines with radium-223 over time. **D.** Spider plot of CellSearch CTC enumeration and changes over time during radium-223 therapy.

In order to determine the relevance of osteomimicry to radium-223 deposition in bone, we conducted a translational pharmacodynamic study of 20 men with symptomatic bone metastatic CRPC between 1/6/15 and 2/27/17, with an administrative cutoff for data analysis on April 8, 2019. These patients were treated with up to 6 monthly injections of radium-223 (**Fig 1**) and contributed correlative CTC and tissue samples. The median age was 72, and 65% were white, 20% black, while 15% had other race/ethnicities. Of these men, 50% had Gleason high-risk 8–10 tumors at diagnosis, 100% had >2 bone metastases and 65% of men had >20 bone metastases at the time of radium-223 treatment. The median baseline PSA was 50 ng/ml, 75% had unfavorable Cellsearch CTC criteria (≥5 CTCs/7.5 mL blood, range 0–252)[19], and 55% of men had elevations in serum alkaline phosphatase at baseline. The men in the study were also heavily pre-treated: 50% had received prior docetaxel, 95% had prior enzalutamide, and 80% had prior abiraterone therapy. Thirty percent of men received concurrent abiraterone or enzalutamide therapy during radium-223 which were continued despite PSA progression on these therapies, and 85% received concurrent denosumab or zoledronic acid. **Table 1** and **Fig 2** describes the patient baseline characteristics and translational study schema, respectively. In the radium-223 study, no unexpected toxicities occurred, and toxicities observed are described in **S1 Table**.

**Table 1:** Baseline characteristics of the patients enrolled.

Baseline Characteristics	Value, % (n = 20)
Age, median years (range)	72 (range 54–86)
Race: white/black/other (%)	65/20/15
Gleason Sum 8–10 (%)	50%
Karnofsky Score ≥90 (%)	65%
Visceral Metastases (%)	5%
Bone metastases (%) %>20 bone metastases	100% 65%
PSA ng/ml (median)	50 (range 2–1896)
Cellsearch CTCs ≥5	75%
Visual Analogue Pain Scale 4–10, opiate use%	30%, 30%
Denosumab or zoledronic acid use	85%
Alkaline phosphatase IU/dl median (range) Alkaline phosphtase %>ULN	119 (41–551) 55%
Prior docetaxel/enzalutamide/abiraterone	50/95/80%

### Clinical outcomes with radium-223

The median number of radium-223 doses received was six, with 11 completing six doses, and 9 men (45%) stopping radium therapy due to progression prior to the receipt of 6 doses. The median progression-free and overall survival on this study was 5.5 months and 15.4 months, respectively (**Fig 2A and 2B**), and 18 men have progressed, while 15 men have died. At 12 months, 20% remained progression-free and 57% of men were alive, while at 24 months, 10% remained progression free and 17% were alive. Overall, 5% had a 30 percent or greater PSA decline, and the majority of men had a rise in PSA as their best response with a median 60% rise in serum PSA observed during therapy. Thirteen percent had a favorable CTC CellSearch conversion from ≥5 to <5 CTCs per 7.5 mL blood [19, 25, 26] although 13 (70%) of men had persistence of >5 CTCs despite radium-223 therapy and only 6 (30%) had ≤5 CTCs at the end of radium-223 therapy. The median CTC change with therapy was a drop of 46% (range -100% to +118%). Cellsearch CTC by subject and time point are provided in **S2 Table**. Serum levels of B-ALP declined by a median of 52% with therapy, and 53% of men had serum levels of B-ALP return to the normal range during treatment. Similar trends were observed with serum total AP levels on treatment, with 75% returning to the normal range; the median percent change in serum total alkaline phosphatase was a 25% decline (**Fig 2C and 2D**).

### Evidence of prostate cancer osteomimicry

**Fig 3:**
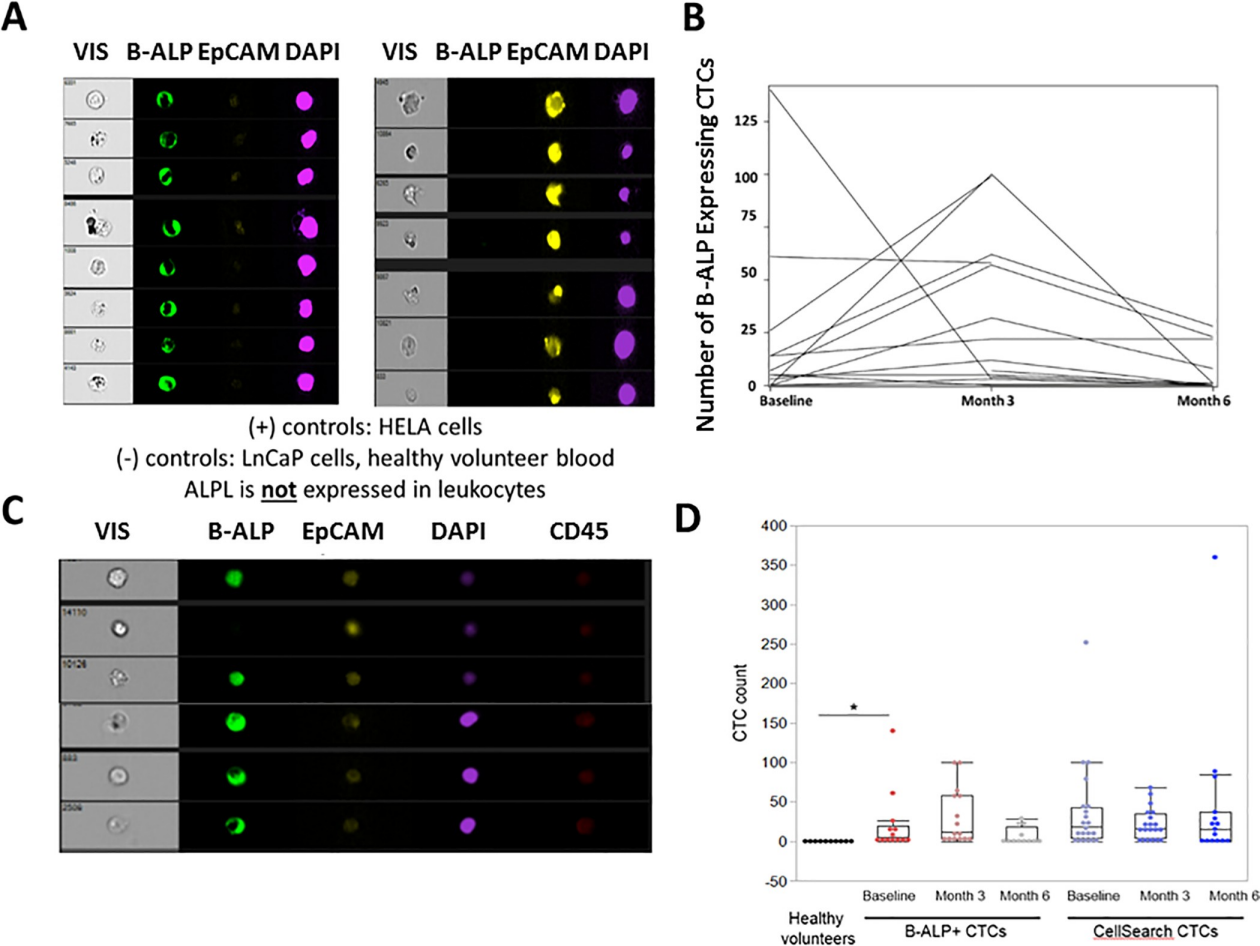
Immunofluorescent micrographs of CTCs expressing bone alkaline phosphatase (B-ALP, or BAP), Epithelial cell adhesion molecular (EpCAM), DAPI, and CD45. **A**. Expression of B-ALP and EpCAM in negative and positive control cell lines spiked into healthy volunteer blood. HELA cells positive for B-ALP (Green) and negative for EpCAM (Yellow) are shown on the left and T47D cells negative for B-ALP and positive for EpCAM are shown on the right. **B.** Changes in CTC B-ALP expression over time during radium-223 therapy. **C.** Examples of CTCs from men with bone metastatic CRPC treated with radium-223, demonstrating heterogeneity of CTC B-ALP expression in CTCs including in EpCAM negative CTCs. **D.** Plots of B-ALP (+) CTCs in healthy volunteers (n = 10) and patients (n = 20) over time, as well as Cellsearch CTCs (n = 20) from patients over time during radium-223 therapy (baseline, month 3 and month 6). *p = 0.0053 for difference in B-ALP (+) CTCs between healthy volunteers and mCRPC patients by non-parametric Kruskal-Wallis test.

To examine the expression of osteomimicry biomarkers, we examined expression of B-ALP in CTCs over time during radium-223 therapy. We utilized the Imagestream platform [27] and conducted studies of positive and negative control cell lines (HeLa for ALPL/ALPL, and LnCAP for EpCAM) spiked into healthy volunteer blood (n = 10). CTCs were defined as CD45 (PTPRC) negative, DAPI positive intact cells; B-ALP (BAP) and concurrent expression of the epithelial molecule EpCAM were then determined. B-ALP or EpCAM positive CTCs were not detected in normal healthy volunteers and were not expressed on normal healthy volunteer leukocytes (0 out of 10 healthy volunteers, median 0 cells). We established gating thresholds for positive B-ALP and EpCAM expression using positive and negative control cells spiked into blood from healthy volunteers (**Fig 3A**) and applied these positive thresholds for patient samples.

We observed evidence of B-ALP expression in CTCs from 69% (9/13) of CTC evaluable men and a median 5 B-ALP+ CTCs per 7.5 mL (range 0–140) (see **Fig 3B–3D**). B-ALP expression in both EpCAM+ and EpCAM- CD45- cells are described per patient and time point in **S2 Table,** and were B-ALP (+) CTCs were significantly higher in patients than healthy volunteers (p = 0.0053 by Kruskal-Wallis test). B-ALP expression was heterogeneous between and within patients, with B-ALP notably expressed in both EpCAM positive and negative CD45 negative cells and absent in normal healthy volunteers (**Fig 3C**). Examples of B-ALP expressing, and B-ALP-negative CTCs are shown in **S1 Fig**.

We tracked CTCs over time during radium-223 therapy, and found that 10/20 (50%) of men had persistently detectable CTCs (both EpCAM+ and EpCAM-) expressing B-ALP, with 25% having a decrease from baseline and 35% an increase from baseline in B-ALP-expressing CTCs (**Fig 3B and 3D**). We found persistence of CTCs over time, with no significant change in mean B-ALP (+) or Cellsearch-defined CTCs over time with radium-223 therapy (p = 0.16 and 0.45, respectively). CTC B-ALP expression did not correlate with serum ALPL expression (Spearman correlation coefficient <0.1) at baseline or at month six. While overall CTC enumeration by Cellsearch declined, the majority of men treated with radium-223 had persistence of both EpCAM positive and negative CTCs by Imagestream, with half of patients having persistent CTC B-ALP expression at the completion of radium-223 therapy despite normalized serum levels of B-ALP (**S2 Table**). The mean number of Cellsearch CTCs at the beginning of radium-223 was 39 and at completion of radium-223 was 44 CTCs per 7.5 mL whole blood. Given the limited sample, size, we are unable to associate CTC ALPL expression with clinical outcomes, as nearly all patients had progressive disease by 6–8 months.

**Fig 4:**
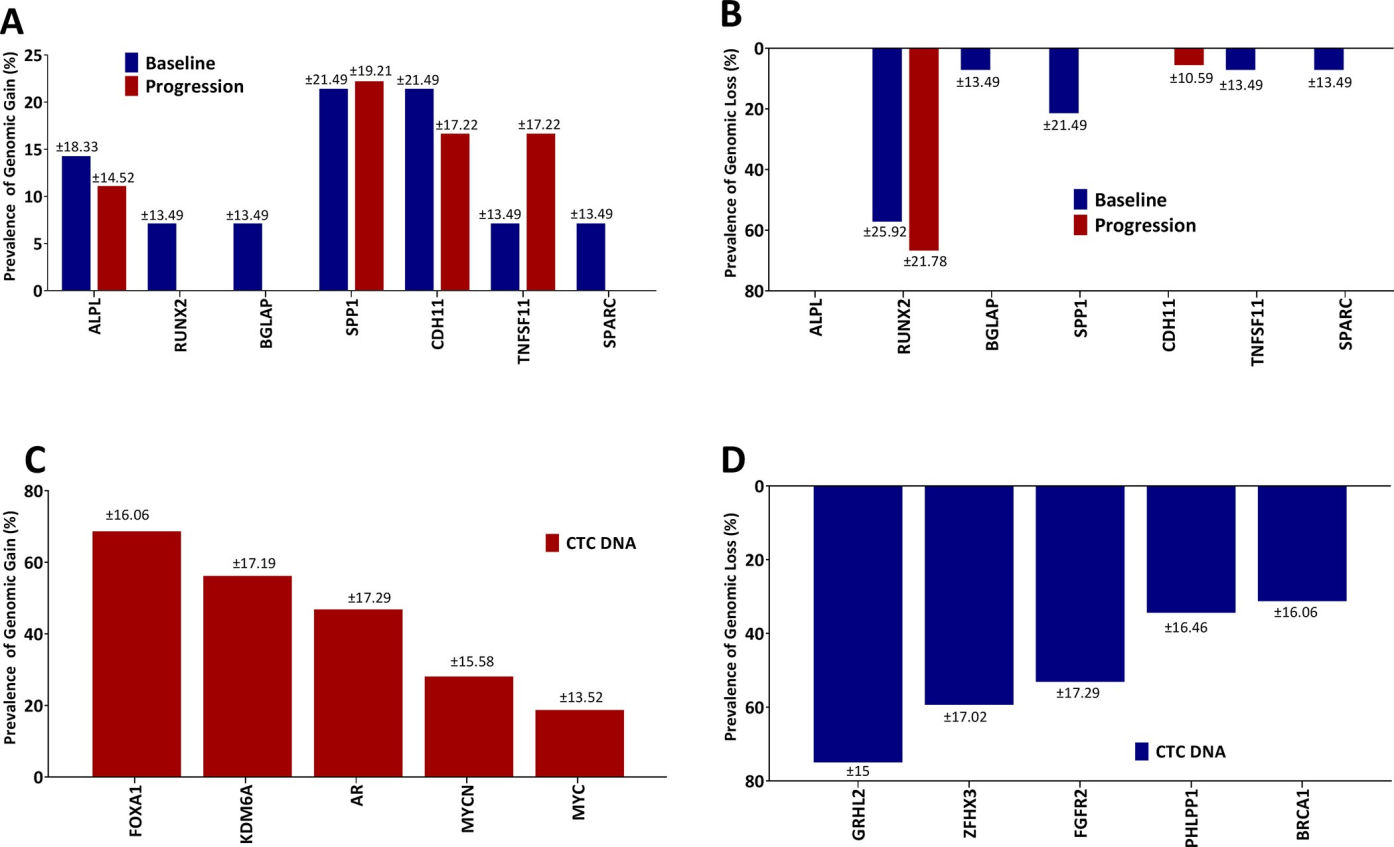
Copy number analysis of osteomimicry genes including **A**. Copy gain, and **B.** Copy loss in CTCs from men with mCRPC treated with radium-223. In addition, top five prevalent genomic alterations by copy gain and loss are shown in **C (**Red bars) and **D** (Blue bars), respectively. ALPL: bone alkaline phosphatase (ALPL), RUNX2: Runt Related Transcription Factor, BGLAP: Bone Gamma Carboxyglutamate Protein, SPP1: Osteopontin, CDH11: OB-Cadherin, TNFSF11: Tumor necrosis superfamily member, SPARC: Secreted Protein Acidic and Cystein Rich.

To understand the genetic basis for the expression of B-ALP (ALPL gene) expression in CTCs and a broader osteomimicry phenotype in prostate cancer CTCs, we analyzed CTC DNA by array-based comparative genomic hybridization (aCGH) using previously established methods[20]. We compared 14 baseline and 18 progression timepoint patient-matched CTC and cfDNA blood samples during radium-223 treatment. We found evidence of copy gain in seven key genes known to play an important role in osteoblast development and physiology: SPP1 (secreted phosphoprotein 1, osteopontin), CDH11 (osteoblast cadherin), ALPL, RUNX2 (runt-related transcription factor 2, CBFA1), BGLAP (bone gamma carboxyglutamate protein, osteocalcin), TNFSF11 (tumor necrosis factor ligand superfamily 11, also known as receptor activator of NF-kappa-B ligand, RANKL), and SPARC (secreted protein acidic and cystein rich, osteonectin). Gains were most common in osteopontin (21%), OB-cadherin (21%), ALPL (14%), and RANKL (7%) at baseline, and the majority of patients had persistence of these genomic gains at progression on radium-223 (**Fig 4A**). Copy loss of RUNX2 was notable in 57% of CTCs at baseline and this copy loss persisted in CTCs collected at progression (**Fig 4B**). In addition, deidentified genomic information from the radium study related to the osteomimicry genes containing copy number alterations by subject ID for external validation are shown in **S1 File**.

In addition to osteomimicry genes, we found common genomic gains in CTCs in key genomic drivers of aggressive mCRPC, including AR (47%), FOXA1 (69%), KDM6A (56%), MYC (19%), and MYCN (28%). (**Fig 4C and S2 Fig**) despite a wide range in CTC number in individual cases. Copy losses were also commonly observed for key tumor suppressors relevant to mCRPC biology, including GRHL2, ZFHX3, FGFR2, NCOR1, PHLPP1, and BRCA1 (**Fig 4D and S2 Fig).**

### Validation studies of osteomimicry

In order to validate the genomic evidence of osteomimicry genetic copy number alterations in a separate dataset of men with mCRPC, we performed aCGH of CTCs from a prospective cohort of men with bone metastases and mCRPC who were undergoing therapy with abiraterone acetate or enzalutamide in a separate IRB approved study (PROPHECY study, NCT02269982). In 45 men, providing 83 CTC samples (45 pre-treatment, and 38 at progression), we found evidence of copy gain of these 7 osteoblastic genes in a significant minority of patients, including ALPL (14.5%), osteopontin (11%), RANKL (2%), SPARC (7%), and OB-cadherin (10%) (**S3A Fig**). Loss of RUNX2 was also commonly observed in CTCs (37%) (**S3B Fig**). In addition, using RNA Sequencing of matched CTCs from the same time point and same patients, we found greater RNA abundance in several key osteomimicry genes (SPARC, BGLAP) in individual patient CTCs as well as loss of RUNX2 RNA expression (**S4 Fig**). ALPL and CDH-11 (OB-Cadherin) RNA abundance in CTCs was low, despite detection of ALPL and prior evidence of CTC CDH-11 protein expression [28], indicating potential post-transcriptional mechanisms of over-expression. We found greater CTC AR RNA abundance by RNA sequencing in CTCs over matched leukocytes, regardless of CTC AR gain, indicating that RNA expression may be disconnected to genomic gain.

We next examined the SU2C public database of 150 evaluable men with mCRPC [29] for genomic alterations in osteomimicry genes and the corresponding RNA levels in metastatic tissue according to metastatic site (bone vs. soft tissue/visceral sites). In order to further validate the functional and phenotypic evidence of osteomimicry gene expression in men with bone metastatic CRPC, we evaluated expression of these same core osteoblastic regulators in public databases according to metastatic site. In the SU2C dataset[29] (**S5 Fig**), we found overexpression of ALPL in 5% of cases, all from men with bone metastases, genomic gain or overexpression of SPARC in 9% of cases predominantly confined to men with bone metastases, and overexpression or genomic alteration of CDH11 and SPP1 in 9% and 3% of cases, respectively, with the majority of overexpressing cases harboring bone metastases. Similarly, analysis of microarray data from bone vs. visceral prostate cancer metastases [30] showed significant over-expression of all osteomimicry genes in bone metastases as compared to visceral metastases. The upregulation of these genes in bone vs. non-bone metastatic sites in these key transcripts ranged from 1.2–3.5 fold. While these data cannot exclude a contribution of bone stroma to the RNA expression profile, these findings are supportive of our direct visualization of ALPL expression in prostate CTCs from men with bone metastatic CRPC and the genomic alterations identified in CTCs from radium-223 treated men with bone metastases in this heavily pre-treated population. Thus, we confirm that osteomimicry gene copy number alterations are detectable in CTC DNA in an independent cohort of men with mCRPC and validate orthogonal phenotypic evidence of osteomimicry at the RNA level in CTCs and tumor biopsies from men with mCRPC.

**Fig 5:**
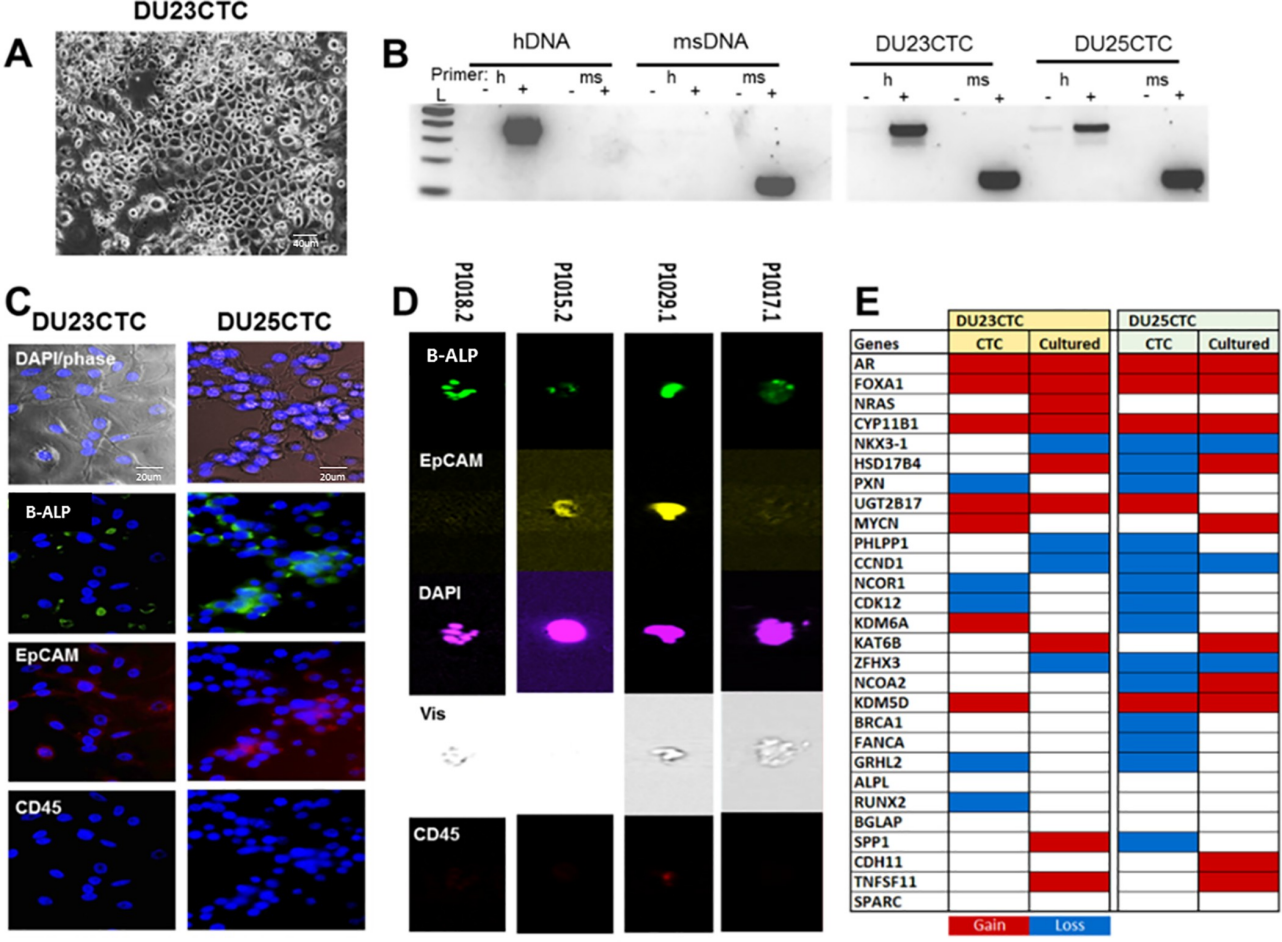
CTC low-passage cultures express ALPL and have common prostate cancer genetic alterations. **A.** Low passage CTC cultures display epithelial-like cobblestone morphology. **B.** A PCR-based assay for human and mouse DNA confirmed the presence of human DNA in the cultures. Human and mouse-specific positive and negative control DNAs (hDNA and msDNA) are shown on the left. L: 2 log ladder; h: human-specific primers; ms: mouse-specific primers. **C.** Immunofluorescence revealed ALPL expression, weak to moderate EpCAM, and no CD45 expression in CTC cultures. **D.** ImageStream analysis of ALPL/ALPL expression in CTCs. **E.** Array competitive genomic hybridization identified copy alterations in mutliple prostate cancer drivers in CTCs and cultures.

We next developed five short-term cultures from negatively selected CTCs from these patients using the irradiated mouse fibroblast/ROCK inhibitor method developed at Georgetown University [24]. While three of these cell lines did not persist long term, two persisted in culture and contained human DNA (called DU23CTC and DU25CTC). Morphologically, cells appeared epithelial, with cobblestone morphology and multiple cell-cell attachments (**Fig 5A**). We confirmed the presence of human cells in the CTC cultures using species-specific PCR for mouse and human DNA (**Fig 5B**). Immunofluorescence studies demonstrated ALPL and EpCAM positivity and lack of CD45 expression in these two cell lines (**Fig 5C**). ImageStream analysis of the CTCs from patients also indicated expression of ALPL in both EpCAM+ and EpCAM- cells (**Fig 5D**). Additionally, we analyzed these two cell lines by aCGH and compared DNA copy gains in these cells to the matched CTCs from the same patients. We found multiple copy number alterations in PC drivers, including gain of AR and FOXA1, consistent with their PC identity and derivation from the same patients (**Fig 5E**).

### Radium-223 uptake in osteoblastic bone metastases

**Fig 6:**
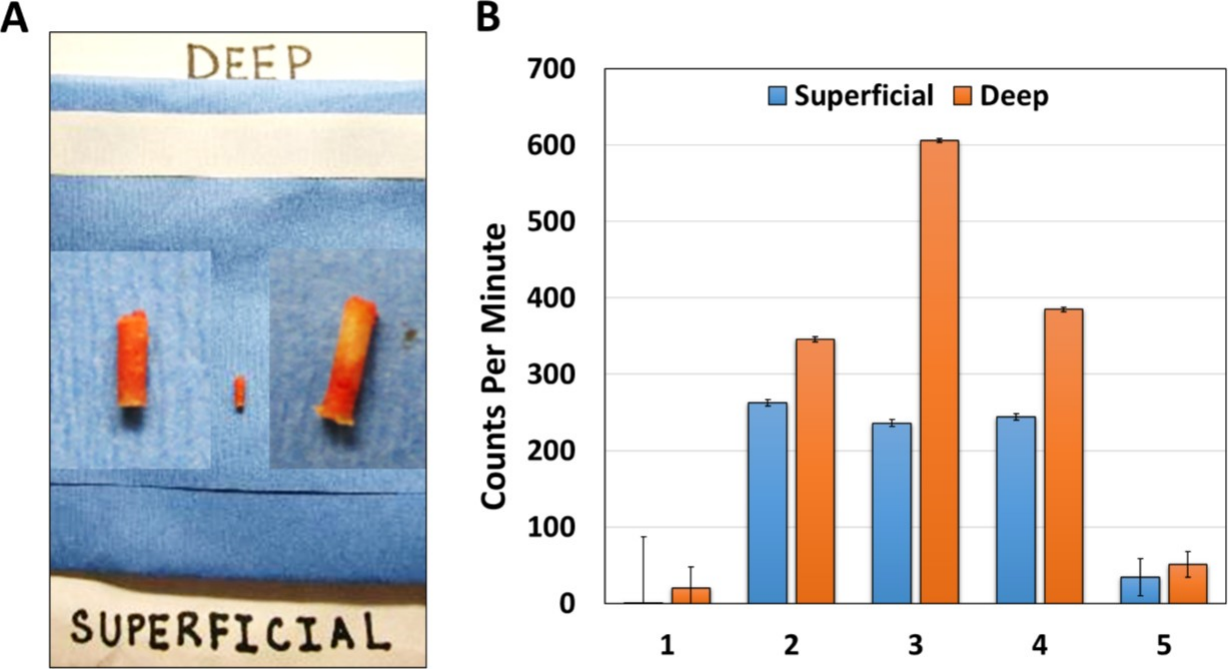
**A.** Examples of CT guided bone biopsy cores divided into superficial and deep sections for gamma emission analysis. **B.** Bar graph of superficial vs. deep bone gamma emission from 5 patients treated with radium-223 and collected after 3 doses. Error bars represent standard error with triplicate replicate samples.

Finally, in order to determine whether radium-223 is targeting the osteoblastic tumor containing regions of bone metastases from these men with mCRPC, we analyzed radium-223 and daughter-radionuclide emissions from the CT guided targeted bone biopsies of men undergoing radium-223 therapy. We utilized prior established methods[31, 32] for CT guided bone biopsies of sclerotic central and peripheral iliac bone metastases using an OnControl Biopsy bone drill system utilizing an 11 gauge introducer and 13 gauge core needle obtained under image guidance. Fresh biopsies were collected following 3 months of therapy (3 doses) and approximately 15 days following the third radium-223 dosing to determine persistent radium-223 and daughter-radionuclide deposition by counting gamma and x-ray emissions from this decay chain[23]. Five patients had sufficient bone biopsies at 3 months post-therapy initiation for this radioactivity counting analysis. In all cases, biopsies were sectioned into the deep tumor targeted biopsy region and the superficial cortical bone biopsy from the same bone drill core (**Fig 6A**). In all cases, gamma and x-ray emission over 10 minutes in triplicate was statistically significantly higher in deep tumor targeted bone biopsies as compared to superficial benign adjacent bone (**Fig 6B**). Representative images of H&E and cytokeratin expression in bone metastases from the time of deep bone biopsy are shown in **S6 Fig**. In two cases, no tumor was detected in either the superficial or deep biopsies, and radioactivity levels were reduced in these biopsies, although still slightly greater in the deep biopsy cores (**Fig 6B**). In three cases, radioactivity levels per gram of tissue were 1.3- to 2.7-fold higher in deep vs. superficial biopsies. This data suggests that radium and daughter radionuclide deposition is higher in tumor containing osteoblastic metastases as compared to sclerotic bone biopsies without visible tumor.

## Discussion

In this pharmacodynamic study, we report clinical phenotypic and genomic evidence of osteomimicry in circulating prostate cancer cells from men with metastatic castration-resistant disease undergoing treatment with radium-223. In parallel, we found evidence of increased radium and daughter radionuclide deposition in osteoblastic bone metastases with detectable tumor cells vs those without. Combined, we conclude that prostate cancer cellular osteomimicry may occur in men with mCRPC and osseous metastases and this may contribute to the intralesional deposition of radium-223 in the prostate cancer bone microenvironment. Osteomimicry may thus underlie some of the clinical benefits of radium-223 in men with bone metastases, and larger validation and mechanistic studies are now needed to confirm and extend this work.

While declines in serum bone alkaline phosphatase correlate with improved survival in men treated with radium-223[10, 11], we found that serum levels of ALPL did not correlate with circulating tumor cell expression of ALPL over time, and that most men treated with radium-223 have persistent CTCs which commonly express ALPL. These data support the concept that serum ALPL may reflect benign osteoblast production in reaction to prostate cancer osteoblastic bone metastases, and that radium-223 reduces this osteoblast production of ALPL, leading to improved clinical outcomes. However, while radium-223 uptake is greater in deep tumor containing biopsies, we observed and validated the persistence of osteomimetic CTCs and CTCs harboring aggressive genomic alterations in men with bone metastatic mCRPC despite radium-223 therapy. These observations, combined with the relatively short time to progression and limited PSA declines observed with radium-223 suggest that additional targeting of key osteomimetic and oncogenic pathways could further improve clinical outcomes. In addition, these osteoblastic metastases likely exhibit relative radioresistance due to the acquisition of genetic alterations that promote cell survival and DNA repair[33–36]. Our data also suggest that the survival benefit observed with radium-223 may be related to the observation that radium-223 targets the tumor microenvironment and osteoblasts given the reductions in serum bone alkaline phosphatase. However, radium-223 may not sufficiently target tumor cells directly, given the lack of a substantial impact on circulating tumor cells and the rapid clinical progressions observed.

In our study, we found that six key osteoblast regulators (ALPL, osteopontin, osteocalcin, OB-cadherin, RANKL, and SPARC) frequently exhibited copy gain and increased RNA expression in CTCs and tumor biopsies from men with bone metastases, while one key osteoblast and osteoclast regulator RUNX2 frequently exhibited copy loss in CTCs. In addition, we validated these findings in an independent cohort of 48 men with bone metastatic CRPC. Supporting this work, we previously identified OB-cadherin as frequently expressed in CTCs from patients with metastatic breast and prostate cancer (18), and OB-cadherin mechanistically promotes metastasis to bone in preclinical models of prostate cancer [37]. Similarly to the other osteomimicry genes, RUNX2 regulates osteoblast differentiation; however, RUNX2 is also a direct suppressor of cell cycle exit during pre-osteoblast proliferation [38]. Thus, loss of RUNX2 in this context might permit osteomimetic prostate cancer cells to gain a proliferative advantage subsequent to seeding in the bone environment. Further functional studies of the contribution of RUNX2 loss to bone metastases is planned.

CTCs may represent a divergent evolutionary branch that promote metastatic spread of cancers, and discovery of novel biomarkers specific to CTC survival and dissemination to target organs such as bone may be needed to prevent or delay metastatic spread. Our findings of osteomimicry in prostate CTCs from men with bone metastases support targeting epithelial plasticity pathways[37, 39–42] such as RANKL, c-MET, OB-cadherin, SNAIL, and chemokine [6] and TGF-beta signaling, which promote such bone tropic spread and survival in the bone marrow microenvironment. Estimates of the prevalence of genomic gains in regions encompassing osteomimicry genes range from 5–35%, with the most commonly gained genes being SPP1 (osteopontin, 35%) and CDH-11 (OB-cadherin, 30%). Osteopontin dysregulation in prostate cancer may cooperate with PTEN and SMAD4 loss to contribute to aggressive and metastatic prostate cancer in transgenic models[43]. However, at the RNA level, the greatest abundance of RNA in CTCs was SPARC (osteonectin), while RUNX2 loss was the most commonly deleted and underexpressed gene in CTCs related to osteoblast differentiation. The inability to observe RUNX2 deletion in bone metastases from public datasets despite the reproducible presence of RUNX2 loss in CTCs from men with bone metastases may reflect tumor heterogeneity, stromal contamination, or differences in detection sensitivity due to probe coverage of the RUNX2 gene. Future larger validation and mechanistic studies as well as single cell studies related to these individual regulators of these key osteomimicry genes and survival and promotion of bone metastasis and hormone and radiation treatment resistance are now needed.

A limitation of our study is the relatively small sample size and correlative nature of our findings. However, recent studies by Rao and colleagues mechanistically supports a key functional role of ALPL and osteomimicry in invasion, epithelial plasticity, and bone metastasis in patients with bone metastatic prostate cancer[14]. While our radium-223 study was not powered to detect clinical differences according to osteomimicry genomic alterations, our study is the first to report on genomic alterations in key osteoblast regulators including ALPL itself in 25% of men with prostate cancer, and supports these prior mechanistic phenotypic studies of ALPL in bone metastases and outcomes. A second limitation is the challenge in validating each of these osteoblast genes functionally in animal models for the promotion of bone metastasis and treatment resistance to radium-223 or other therapies. Animal models of prostate cancer osteoblastic bone macrometastasis derived from hematogenous spread of tumors do not presently exist despite many attempts[43–47], but our findings suggest the idea that genetically engineered models of prostate cancer harboring overexpression or amplification of osteopontin, OB-cadherin, SPARC, RANKL, or osteocalcin or deletion of RUNX2 may lead to osteoblastic bone metastases. Indeed, recent work on OB-cadherin overexpression and knock-down in cellular and animal models of prostate cancer supports a mechanistic role of this adhesion molecule in mediating bone metastasis[48]. This, coupled with our prior observations of OB-cadherin expression in prostate CTCs from men with bone metastases[28] supports the importance of this adhesion molecule in bone metastasis formation or maintenance.

We found evidence of genomic gain in approximately 17% of CTCs of TNFSF11, also known as receptor activator of NF-kappa-beta ligand (RANKL). The osteoblastic regulator RANKL is clearly important in prostate cancer bone metastasis biology[42], and the clinical targeting of this pathway with denosumab, a RANKL inhibitory monoclonal antibody, has been shown to delay bone metastasis and reduce the complication rates from bone metastases in patients[49]. RANKL also mediates epithelial plasticity in prostate cancer, and the co-targeting of several of these pathways may be necessary to prevent bone metastases[42]. Indeed, targeting RANKL early in prostate cancer bone metastases may be critical to protecting tumors from fractures associated with the decrease of osteoblastic activity from radium-223. A recent clinical trial (ERA-223) was discontinued early due to an increased fracture rate associated with the combination of radium-223 and abiraterone and prednisone (November 30, 2017 Bayer press release). Our patients were more heavily pretreated, and were largely resistant to AR targeting therapy, and most men had a significant tumor burden (> 20 osseous metastases) and well established sclerotic lesions. These more established and treatment resistant bone metastases may be less susceptible to fracture as a result to osteoblast and osteomimicry targeting than newly formed osteoblastic metastases that may respond more dramatically to hormonal therapies.

In conclusion, we have found genomic and phenotypic evidence supporting prostate cancer osteomimicry in circulating tumor cells from men with mCRPC and bone metastases. This evidence is supported by commonly observed genomic gains and higher RNA expression of key osteomimicry regulators as well as additional phenotypic evidence of bone alkaline phosphatase protein expression in CTCs and CTC derived cell cultures. The implications of these findings is that osteomimicry may contribute to the mechanisms of bone metastasis but can also be leveraged for bone targeting therapies as well as therapeutics designed to target plasticity pathways that regulate osteoblastic differentiation in prostate cancer. We also find that the genomic characterization of CTCs permitted the discovery of these novel findings, illustrating the importance of studying these potential seeds of metastasis for understanding tumor biology and progression. Our findings support a personalized approach to prevention or targeting of bone metastases in prostate cancer that account for the genetic dysregulation of key plasticity pathways important for bone metastasis and bone targeted therapies.

## Supporting information

S1 Table Toxicity summary related to radium-223 (n = 20) by grade. (DOCX)

S2 Table Summary of Cellsearch and Imagestream CTCs by subject and time point. BAP = B-ALP (bone alkaline phosphatase, referring to the protein product of the ALPL gene). EpCAM = Epithelial cell antigen. Number of Cellsearch CTCs and B-ALP (+) CTCs detected in patients with mCRPC (n = 20) at baseline, month 3, and month 6 of radium-223 therapy. Both EpCAM(+) and (-) CTCs by Imagestream are shown. Numbers are per 7.5 mL whole blood. X = missing sample or unevaluable sample.
(DOCX)

S1 Fig Images of selected patient CTCs expressing B-ALP (BAP), EpCAM, nuclear DAPI, and lacking CD45 using the Imagestream platform. **A**. Sorting of cells by size, DAPI expression, lack of CD45 expression, and EpCAM (E) or B-ALP (A) expression. **B.** Images of EpCAM (+) CTCs in yellow and B-ALP (+) CTCs in green are shown. Examples are taken from patient blood samples from the pharmacodynamics study of radium-223 in men with bone metastatic CRPC.
(TIF)

S2 Fig Heat map demonstrating copy gains and losses by arrayCGH of key genomic regions in CTCs from men with bone metastatic CRPC undergoing radium-223. Red indicates copy gain and blue indicates copy loss, while white indicates copy neutral status. Patients are denoted in columns while genes are denoted in rows. The prevalence of genomic alterations is indicated in the far right columns and the number of CTCs at the time of blood collection is noted in the bottom row.
(TIF)

S3 Fig Validation of copy number analysis of key osteomimicry genes gained in CTCs (Blue bars in A) or lost (Blue bars in B) from men with bone metastatic mCRPC treated (n = 83, PROPHECY study).
(TIF)

S4 Fig Plots of RNA expression by RNA sequencing of CTCs and patient matched leukocytes according to copy gain status in CTCs. **A.** AR; **B**. RUNX2; **C**. SPARC; **D.** ALPL; **E**. BGLAP; **F**. SPP1; **G.** CDH-11; **H.** TNFSF11 (RANKL). Ficoll = normal peripheral blood mononuclear cells (PBMCs) from the same patients.
(TIF)

S5 Fig Heatmap demonstrating genomic alterations in metastatic biopsies of men with mCRPC according to site of biopsy (bone vs. soft tissue/visceral metastases). A. Selected genomic alterations in key osteomimicry genes are shown according to DNA amplification or deletion, mutation, or mRNA upregulation. B. Fold change of selected mRNA species in bone metastases as compared to soft tissue/visceral metastases.
(TIF)

S6 Fig A-D. Photomicrographs of two deep bone metastatic biopsies taken during the radium-223 trial demonstrating tumor content by hematoxylin and eosin (H&E, left A and C) and pan-cytokeratin (right B and D) expression.
(TIF)

S1 File Heat map illustrating the osteomimicry genes containing copy number alterations by subject ID from radium-223 study (red-gain, blue-loss and while-copy neutral). (XLSX)

S2 File Supplementary Data. Additional descriptions of methods including clinical trial eligibility and additional details of human vs. mouse primer methods used for the CTC cell lines.
(DOCX)

S3 File TREND Checklist. Completed checklist providing the location of key aspects of the required study elements.
(PDF)

S4 File Radium-223 Clinical Protocol. Included is the final radium-223 pharmacodynamic study clinical protocol, redacted to remove confidential information.
(PDF)
