# Supplementary material for: Pharmacodynamic study of radium-223 in men with bone metastatic castration resistant prostate cancer
Source: PLoS One. 2019 May 28;14(5):e0216934. doi: 10.1371/journal.pone.0216934 (PMC6538141; doi:10.1371/journal.pone.0216934)
Supplement: S1 Table — (DOCX) [file pone.0216934.s001.docx]

| **Adverse Event term**  **(at least possibly related)** | **Grade 1 and 2**  **frequency in % (n)** | **Grade 3**  **frequency in % (n)** |
| --- | --- | --- |
| Fatigue | 40 (8) | 5 (1) |
| Pain | 30 (6) | 5 (1) |
| Anemia | 10 (2) | 25 (5) |
| Dehydration | 5 (1) | 5 (1) |
| Thromboembolic event |  | 5 (1) |
| Confusion |  | 5 (1) |
| Hypokalemia |  | 5 (1) |
| Peripheral nerve infection |  | 5 (1) |
| Nausea | 40 (8) |  |
| Anorexia | 25 (5) |  |
| Weight loss | 15 (3) |  |
| Hypoalbuminemia | 15 (3) |  |
| Diarrhea | 10 (2) |  |
| Dyspepsia | 10 (2) |  |
| Vomiting | 10 (2) |  |
| Dizziness | 10 (2) |  |
| Dysgeusia | 10 (2) |  |
| Insomnia | 10 (2) |  |
| Fall | 5 (1) |  |
| Platelet count decreased | 5 (1) |  |
| Gastroesophageal reflux disease | 5 (1) |  |
| Sinus tachycardia | 5 (1) |  |
| Gastrointestinal disorders - Other, specify | 5 (1) |  |
| Urinary Tract Infection | 5 (1) |  |
| Anthralgia | 5 (1) |  |
| Generalized muscle weakness | 5 (1) |  |
| Anxiety | 5 (1) |  |
| Dyspnea | 5 (1) |  |
| Rash maculo-papular | 5 (1) |  |
| Hypertension | 5 (1) |  |

**Supplementary Table 1**. Toxicity summary related to radium-223 (n=20) by grade.
