# Supplementary material for: Pharmacodynamic study of radium-223 in men with bone metastatic castration resistant prostate cancer
Source: PLoS One. 2019 May 28;14(5):e0216934. doi: 10.1371/journal.pone.0216934 (PMC6538141; doi:10.1371/journal.pone.0216934)
Supplement: S2 Table — BAP = B-ALP (bone alkaline phosphatase, referring to the protein product of the ALPL gene). EpCAM = Epithelial cell antigen. Number of Cellsearch CTCs and B-ALP (+) CTCs detected in patients with mCRPC (n = 20) at baseline, month 3, and month 6 of radium-223 therapy. Both EpCAM(+) and (-) CTCs by Imagestream are shown. Numbers are per 7.5 mL whole blood. X = missing sample or unevaluable sample. (DOCX) [file pone.0216934.s002.docx]

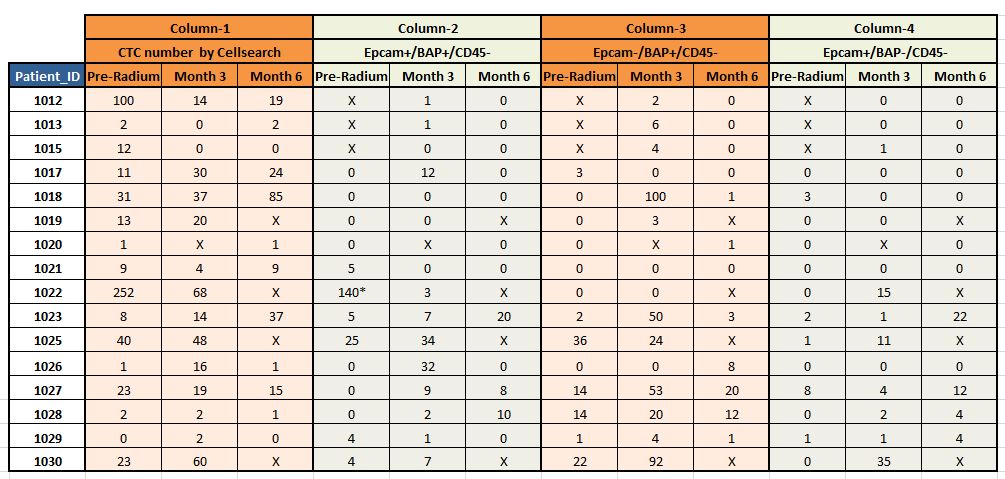


**S2 Table**. Summary of Cellsearch and Imagestream CTCs by subject and time point. BAP=ALPL (bone alkaline phosphatase). EpCAM=Epithelial cell antigen.
