# Supplementary figures and images for: Pharmacodynamic study of radium-223 in men with bone metastatic castration resistant prostate cancer
Source: PLoS One. 2019 May 28;14(5):e0216934. doi: 10.1371/journal.pone.0216934 (PMC6538141; doi:10.1371/journal.pone.0216934)

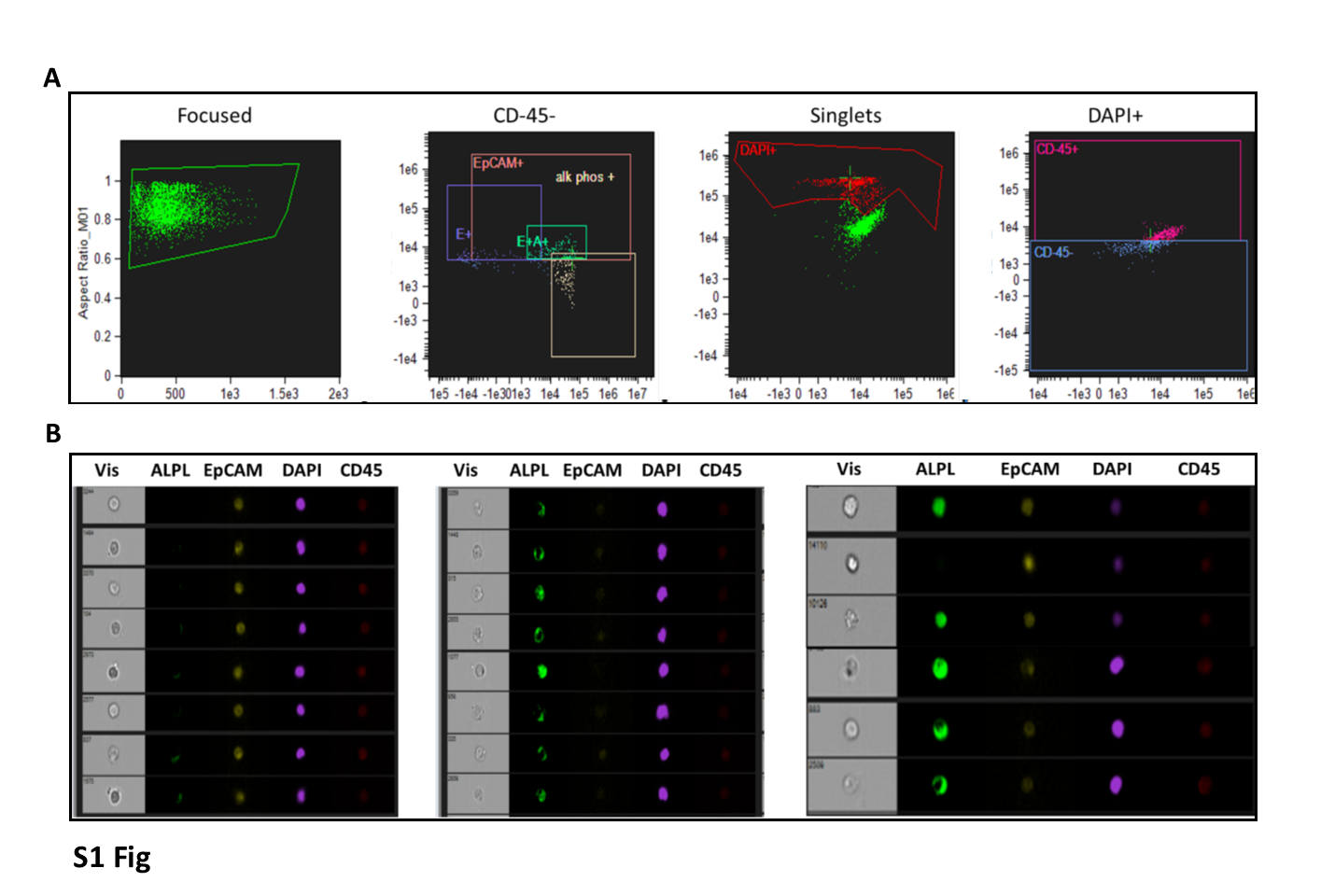

Supplement: S1 Fig — A. Sorting of cells by size, DAPI expression, lack of CD45 expression, and EpCAM (E) or B-ALP (A) expression. B. Images of EpCAM (+) CTCs in yellow and B-ALP (+) CTCs in green are shown. Examples are taken from patient blood samples from the pharmacodynamics study of radium-223 in men with bone metastatic CRPC. (TIF) [file pone.0216934.s003.tif]

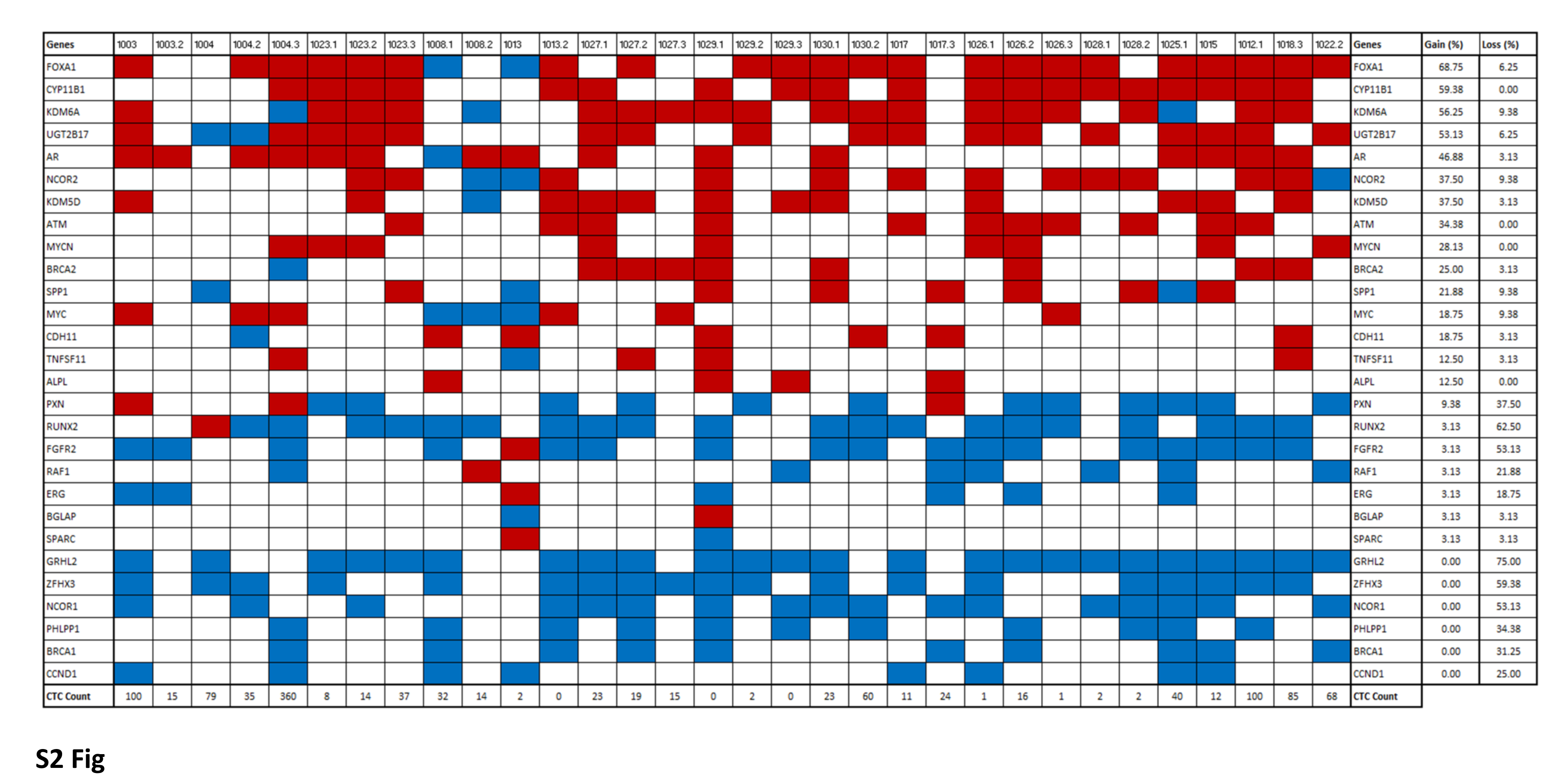

Supplement: S2 Fig — Red indicates copy gain and blue indicates copy loss, while white indicates copy neutral status. Patients are denoted in columns while genes are denoted in rows. The prevalence of genomic alterations is indicated in the far right columns and the number of CTCs at the time of blood collection is noted in the bottom row. (TIF) [file pone.0216934.s004.tif]

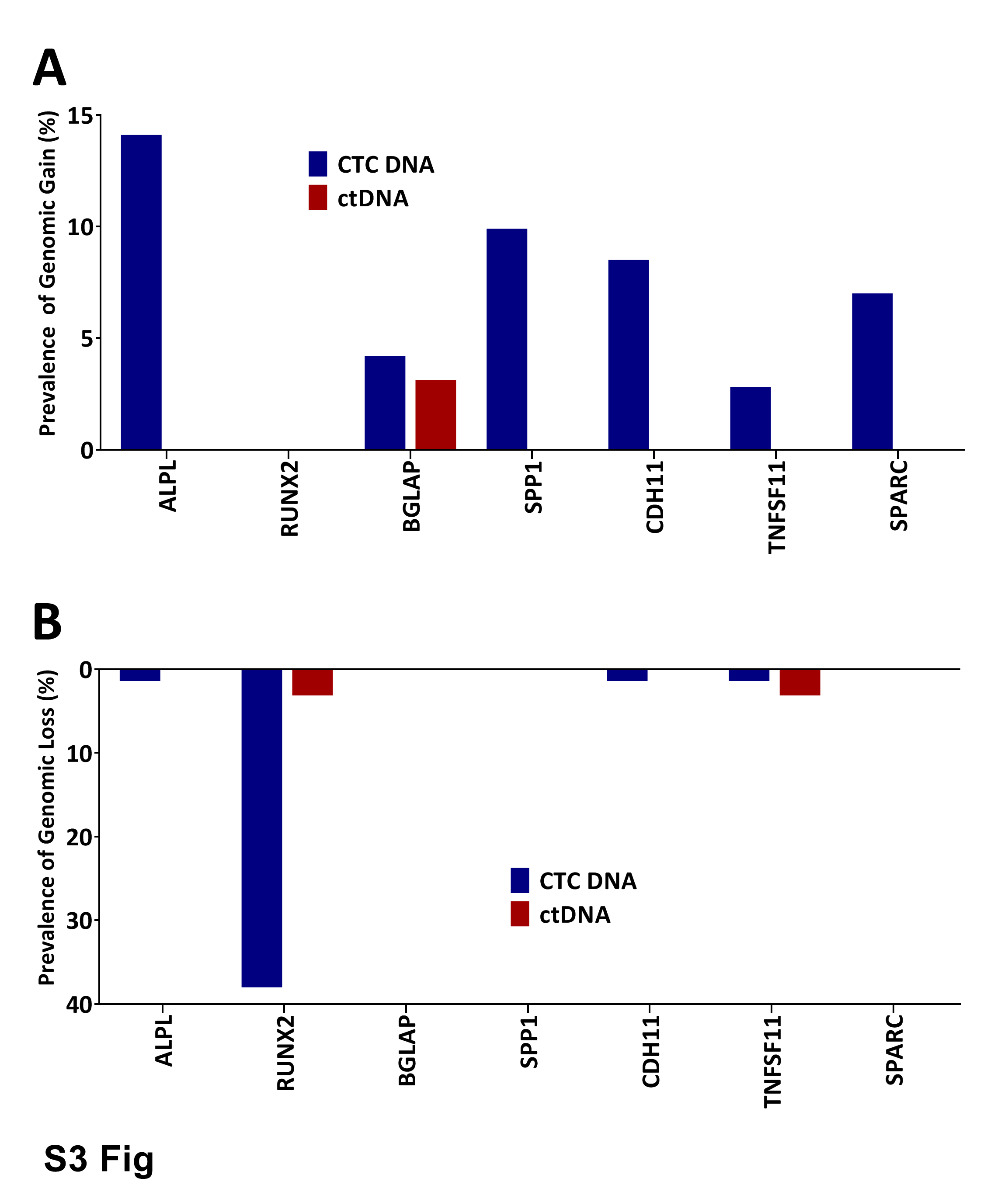

Supplement: S3 Fig — Validation of copy number analysis of key osteomimicry genes gained in CTCs (Blue bars in A) or lost (Blue bars in B) from men with bone metastatic mCRPC treated (n = 83, PROPHECY study). (TIF) [file pone.0216934.s005.tif]

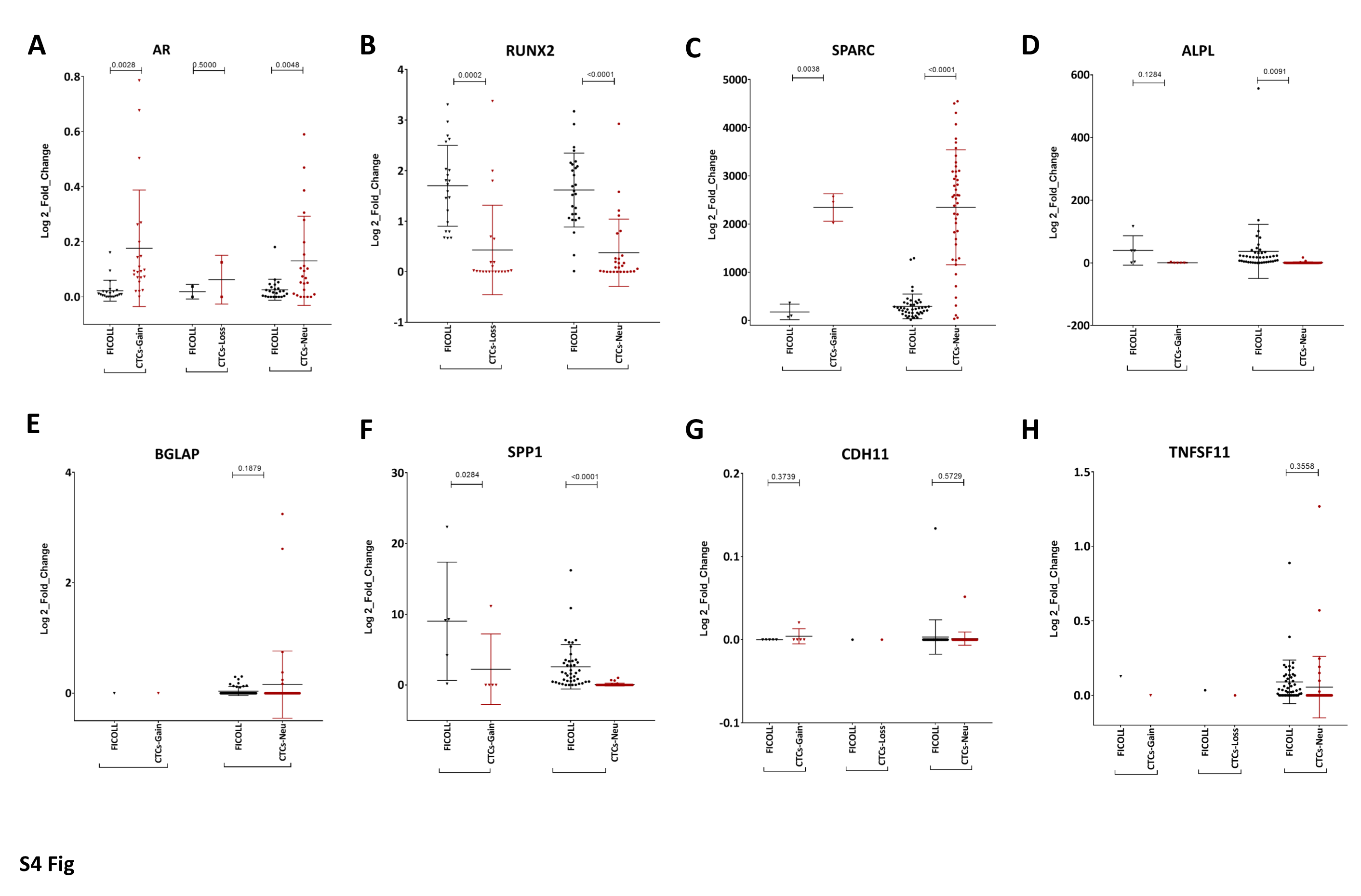

Supplement: S4 Fig — A. AR; B. RUNX2; C. SPARC; D. ALPL; E. BGLAP; F. SPP1; G. CDH-11; H. TNFSF11 (RANKL). Ficoll = normal peripheral blood mononuclear cells (PBMCs) from the same patients. (TIF) [file pone.0216934.s006.tif]

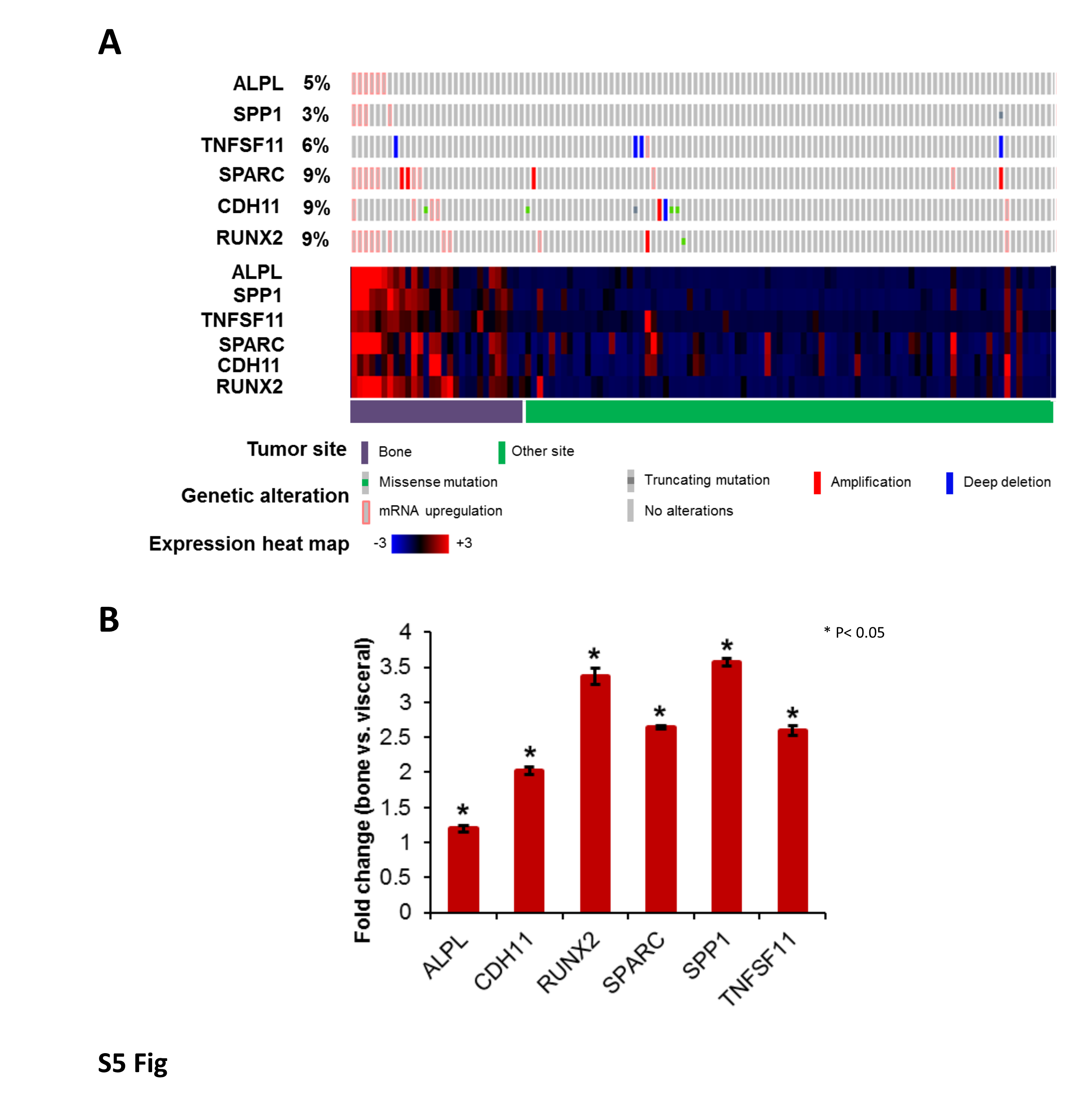

Supplement: S5 Fig — A. Selected genomic alterations in key osteomimicry genes are shown according to DNA amplification or deletion, mutation, or mRNA upregulation. B. Fold change of selected mRNA species in bone metastases as compared to soft tissue/visceral metastases. (TIF) [file pone.0216934.s007.tif]

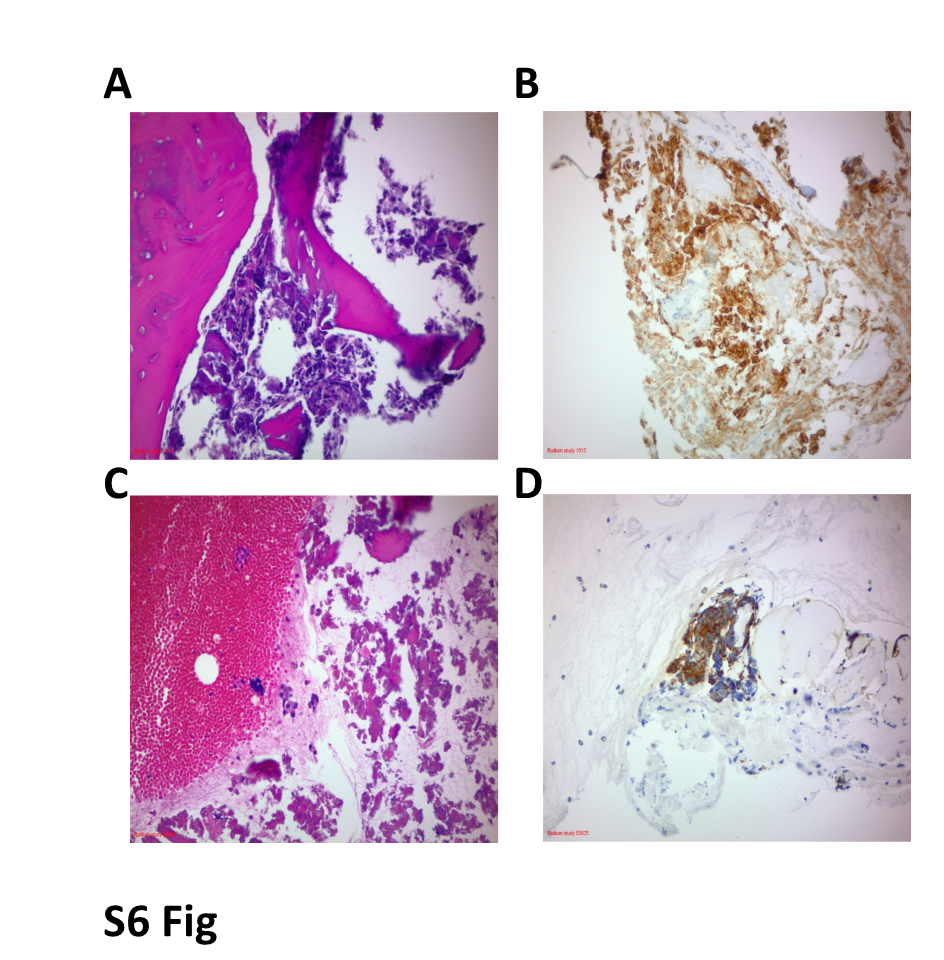

Supplement: S6 Fig — A-D. Photomicrographs of two deep bone metastatic biopsies taken during the radium-223 trial demonstrating tumor content by hematoxylin and eosin (H&E, left A and C) and pan-cytokeratin (right B and D) expression. (TIF) [file pone.0216934.s008.tif]
