# Supplementary material for: Pharmacodynamic study of radium-223 in men with bone metastatic castration resistant prostate cancer
Source: PLoS One. 2019 May 28;14(5):e0216934. doi: 10.1371/journal.pone.0216934 (PMC6538141; doi:10.1371/journal.pone.0216934)
Supplement: S2 File — Additional descriptions of methods including clinical trial eligibility and additional details of human vs. mouse primer methods used for the CTC cell lines. (DOCX) [file pone.0216934.s010.docx]

**Supplementary Methods**

For the radium-223 pharmacodynamic study (n=20), patients were excluded if they had inadequate bone marrow function defined as a white blood cell count of <3,000/mm^3^, an absolute neutrophil count (ANC) <1,500/mm^3^, platelet count <100,000/mm^3^, or hemoglobin <9.0 g/dl. Adequate renal and hepatic function were required and a wash-out period of 4 weeks from cytotoxic chemotherapy was required. Patients with visceral (lung, liver) metastases were excluded if lung nodules were >1 cm by longest diameter, as were patients with active or untreated CNS or epidural metastases and lymphadenopathy >3 cm by short axis diameter measurements.

For the PROPHECY validation cohort (n=45), we prospectively enrolled men with metastatic castration-resistant prostate cancer who were beginning standard-of-care treatment with enzalutamide or abiraterone. Prior exposure to enzalutamide or abiraterone was permitted for men who were planning to receive the opposite agent. Patients were required to have histologically confirmed prostate adenocarcinoma, progressive disease despite castration levels of serum testosterone (<50 ng per deciliter [1.73 nmol per liter]) with continued androgen deprivation therapy, and documented metastases as confirmed on computed tomography (CT) or bone scanning with technetium-99m–labeled methylene diphosphonate. Patients had to have three or more rising serum PSA values obtained 2 or more weeks apart, with the last value being 2.0 ng per milliliter or higher — criteria for PSA progression that are consistent with Prostate Cancer Clinical Trials Working Group 2 (PCWG2) guidelines. In addition, patients were required to have two or more high-risk, poor prognosis features including anemia with a hemoglobin <12.0 g/dl, elevated alkaline phosphatase above the institution upper limit of normal, elevated serum LDH above the institutional upper limit of normal, prior therapy with enzalutamide or abiraterone, presence of visceral metastasis on imaging, presence of clinically significant pain requiring opiate analgesics, Cellsearch CTC counts of >5 cells per 7.5 mL, a PSA doubling time of <3 months, or radiographic progression at entry. Patients were excluded if they planned to receive additional concurrent anticancer therapies. Prior docetaxel chemotherapy was permitted in the hormone-sensitive treatment setting.

Human vs. mouse DNA primers for cell line characterization

We assessed the presence of human vs. mouse DNA using human specific primers (F-TAG ACA TCG TAC ACG ACA CG; R-TCC AGG TTT ATG GAG GGT TC) or mouse specific primers (F-ATT ACA GCC GTA CTG CTC CTC CTA T; R-CCC AAA GAA TCA GAA CAG ATT C). Cycling conditions were 95°C for 2 min, followed by 35 cycles of 94°C for 15 s, 55°C for 50 s, and 72°C for 90 s, followed by 72°C for 7 min and a hold at 4°C using the PCR Core Kit (Cat #: 11578553001). Following PCR, reactions were run on a 1% agarose gel and imaged with the Licor Odyssey Imager.
