# Supplementary material for: Pharmacodynamic study of radium-223 in men with bone metastatic castration resistant prostate cancer
Source: PLoS One. 2019 May 28;14(5):e0216934. doi: 10.1371/journal.pone.0216934 (PMC6538141; doi:10.1371/journal.pone.0216934)
Supplement: S4 File — Included is the final radium-223 pharmacodynamic study clinical protocol, redacted to remove confidential information. (PDF) [file pone.0216934.s012.pdf]

DUKE CANCER INSTITUTE

A National Cancer Institute-designated Comprehensive Cancer Center

Pharmacodynamic Study of Radium-223 in Men with Bone Metastatic Castration-Resistant Prostate Cancer (CRPC)

Sponsor:	Andrew J Armstrong MD ScM Duke Cancer Institute
Funding Source:	Bayer Healthcare
Protocol Source:	Andrew J Armstrong MD ScM Duke Cancer Institute
Duke IRB#:	Pro00053925
IND#:	exempt

Version 1:	05/23/2014	Version 11:	03/17/16
Version 2:	09/09/2014	Version 12:	05/04/16
Version 3:	10/14/14	Version 13:	10/07/16
Version 4:	12/08/14	Version 14:	11/17/16
Version 5:	01/14/15	Version 15:	8/23/17
Version 6:	03/11/15		
Version7:	03/18/15		
Version 8:	08/19/15		
Version 9:	02/17/16 (NIST update)		
Version 10:	02/24/16		

Table of Contents

1 LIST OF ABBREVIATIONS 5

2 SYNOPSIS 6

3 STUDY SCHEMA 12

4 BACKGROUND AND SIGNIFICANCE 12

4.1 Study Disease 12

4.2 Study Agent: Radium-223 dichloride 13

4.2.1 Description 13

4.2.2 National Institute of Standards and Technology (NIST) Standardization 14

4.2.3 Mechanism of Action 15

4.2.4 Pre-clinical experience 15

4.2.5 Clinical experience 15

4.2.6 Rationale for US National Institute of Standards and Technology (NIST) Update . 18

4.3 Study Purpose/Rationale 19

5 OBJECTIVES AND ENDPOINTS 20

6 INVESTIGATIONAL PLAN 21

6.1 Study Design 21

6.2 Selection of Dose and Treatment Duration 22

6.3 Subject eligibility 23

6.3.1 Inclusion criteria 23

6.3.2 Exclusion Criteria 24

6.4 Patient Registration 25

6.4.1 Informed Consent 25

6.5 Early Study Termination 25

7 STUDY DRUG 25

7.1 Names, Classification, and Mechanism of Action 25

7.2 Labeling 26

7.3 General warning 26

7.4 Radiation protection 26

7.5 Dose calibration 27

7.6 Dosimetry 27

7.7 Dose handling 27
7.8 Dose calculation 28
7.9 Dose preparation 28
7.10 Dose administration 28
7.11 Dose Modification 28
7.12 Compliance and Accountability 29
7.13 Prior and Concomitant Therapy 29
7.14 Disposal and Destruction 30
8 SCREENING AND ON-STUDY TESTS AND PROCEDURES 31
8.1 Screening Examination 33
8.2 Treatment Period 33
8.3 End of Treatment 34
8.4 Interruption or discontinuation of treatment 34
8.5 Early Withdrawal of Subject(s) 35
8.5.1 Criteria for Early Withdrawal, drop outs/screen failures 35
8.5.2 Recording Requirements for Early Withdrawal 35
8.5.3 Replacement of Early Withdrawal(s) 35
8.6 Study Assessments 35
8.6.1 Medical History 35
8.6.2 Vital signs, Performance status and Physical Exam 36
8.6.3 Laboratory Evaluations 36
8.6.4 Imaging 36
8.6.5 Exploratory correlative studies 36
9 SAFETY MONITORING AND REPORTING 39
9.1 Adverse Events 40
9.2 Serious Adverse Events 40
9.3 Adverse Events Summary 41
9.4 Pregnancies 43
9.5 Safety Oversight Committee (SOC) 43
10 QUALITY CONTROL AND QUALITY ASSURANCE 43
10.1 Monitoring 43
10.2 Data Management and Processing 43
11 STATISTICAL METHODS AND DATA ANALYSIS 45

12 ADMINISTRATIVE AND ETHICAL CONSIDERATIONS 46
12.1 Regulatory and Ethical Compliance 46
12.2 DUHS Institutional Review Board and DCI Cancer Protocol Committee 46
12.3 Informed Consent 46
12.4 Privacy, Confidentiality, and Data Storage 46
12.5 Data and Safety Monitoring 47
12.6 Protocol Amendments 47
12.7 Records Retention 47
12.8 Publication policy 47
12.9 Conflict of Interest 47
13 References 49
14 APPENDICES 51
14.1 Performance Status 51
14.2 NCI COMMON TOXICITY CRITERIA 51
14.3 CRITERIA FOR DISEASE PROGRESSION 52

1 LIST OF ABBREVIATIONS

aCGH	Array comparative genomic hybridization
AE	Adverse Event
ALT	Alanine Aminotransferase
ALC	Absolute Lymphocyte Count
ALP	Alkaline phosphatase
AST	Aspartate Aminotransferase
BUN	Blood Urea Nitrogen
CBC	Complete Blood Count
CK	Cytokeratin
CMP	Comprehensive Metabolic Panel
CR	Complete Response
CRPC	Castration resistant prostate cancer
CT	Computed Tomography
CTC	Circulating tumor cell
CTCAE	Common Terminology Criteria for Adverse Events
DLT	Dose Limiting Toxicity
DSMB	Data and Safety Monitoring Board
ECOG	Eastern Cooperative Oncology Group
FISH	Fluorescene in situ hybridization
γ-H2AX	gamma H2AX
H&P	History & Physical Exam
HRPP	Human Research Protections Program
IHC	Immunohistochemistry
IV (or iv)	Intravenously
LDH	Lactate dehydrogenase
MTD	Maximum Tolerated Dose
NCI	National Cancer Institute
NIST	National Institutes of Standards and Technology
ORR	Overall Response Rate
OS	Overall Survival
PBMCs	Peripheral Blood Mononuclear Cells
PD	Progressive Disease
PFS	Progression Free Survival
p.o.	per os/by mouth/orally
PR	Partial Response
PSA	Prostate specific antigen
RT-PCR	Reverse transcription polymerase chain reaction
SAE	Serious Adverse Event
SD	Stable Disease
SGOT	Serum Glutamic Oxaloacetic Transaminase
SPGT	Serum Glutamic Pyruvic Transaminase
WBC	White Blood Cells
WES	Whole exome sequencing

2 SYNOPSIS

Purpose: The purpose of this study is to examine biomarkers of osteomimicry in bone metastases and circulating tumor cells (CTCs) of men with mCRPC before and during therapy with the bone-targeting radiopharmaceutical radium-223. This study will also examine the bio-distribution of radium-223 in bone and bone metastases of men with mCRPC.

Hypotheses:
1. We hypothesize that bone metastases and CTCs in men with mCRPC will commonly express markers of EMT/plasticity and osteomimicry, not just in the normal surrounding osteoblastic stroma but in the epithelial tumor cells themselves. 2. We hypothesize that radium-223 will target both of these compartments including the more mesenchymal/osteoblastic tumor cells and the surrounding osteoblasts in the active bone microenvironment, with a relative sparing of normal bone and bone marrow.

Background and Significance: Men with metastatic CRPC in 2013 have an improved prognosis based on therapeutic advances in immunotherapy (sipuleucel-T), hormonal therapies (abiraterone and enzalutamide), chemotherapy (docetaxel, cabazitaxel), and bone targeting radium-223 (Clark, 2013). Existing prognostic models have been developed in older eras when the majority of these treatments were not available and were limited to men treated with systemic chemotherapy (Halabi, 2003; Halabi, 2007; Smaletz, 2002) or post-chemotherapy (TAX327 post-docetaxel model)(Armstrong, 2007). While prognosis remains important, prediction of benefit with a systemic therapy is of greater importance, as knowledge of a biomarker is tied directly to a therapeutic implication and greater potential to maximize benefit and minimize harms and costs (Green, 2012). Thus, predictive biomarkers are needed in men with mCRPC.

Radium-223 has demonstrated improved overall survival (OS) in men with symptomatic bone-metastatic CRPC in the post-docetaxel and docetaxel ineligible population (Harrison, 2013; Nilsson, 2012; Nilsson, 2013; Parker, 2012; Parker, 2013). Baseline osteoblast activity biomarker alkaline phosphatase (ALP) has been reported to be prognostic (men with high alkaline phosphatase had shorter survival) in men with mCRPC (Sonpavde, 2010; Nilsson, 2013). In addition, there was a suggestion of predictive role for baseline ALP in that men with higher baseline ALP had a substantial and statistically significant benefit with radium-223 over placebo plus support care, while men with normal/low baseline AP had no clear clinical or statistical benefit (Nilsson, 2013; Parker 2013). These findings suggest that elevations in osteoblast activity, which may be linked to greater uptake and pharmacodynamics effects of radium-223 in bone, may be predictive of the benefits of this drug for improved OS. These findings have mechanistic support, biologic plausibility, and offer compelling evidence that may improve the efficacy and delivery and reduce the toxicity and costs of radium-223 in the clinic. However, it is currently unclear what the source of the alkaline phosphatase elevations are in men with bone-metastatic CRPC.

The osteomimicry hypothesis was first developed in 1999 and has largely been confined to date to preclinical and in vitro models rather than in patients (Koeneman, 1999). We propose that prostate cancer cells from men with bone metastatic CRPC will commonly express markers of EMT and osteomimicry through a biologic process termed epithelial plasticity, and that understanding this process will help direct the therapeutic potential of radium-223. Direct visualization of co-expression of epithelial markers with bone markers in metastases and CTCs will provide evidence for the osteomimicry hypothesis and the potential for radium-223 to target the metastatic tumor directly. In addition, the evaluation and visualization of the radium-223 localization and its decay products in bone metastatic lesions and the surrounding micro and macroenvironment will provide insights into the target (tumor, host, or both) of this radiopharmaceutical.

Design and Procedure: This will be a prospective, pharmacodynamic biomarker study of men with multifocal bone metastatic CRPC with disease progression, inclusive of men in the pre-docetaxel and post-docetaxel treatment space. Radium-223 will be given to all men, who will be followed until disease progression as per standard of care practice. Men will be allowed to continue concurrent use of standard of care hormonal therapies (androgen deprivation therapy, abiraterone acetate or enzalutamide), anti-resorptive therapies, and corticosteroids. Eligible men must have disease progression (radiographic, symptomatic, or PSA based progression) prior to entry.

See below schema for the clinical trial design.

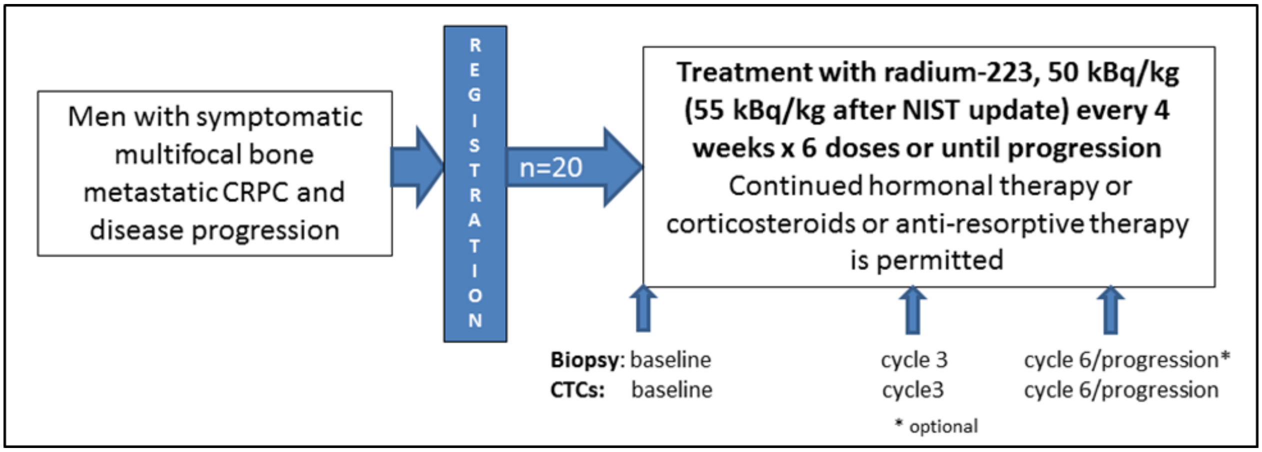

Selection of Subjects: Patients must have baseline evaluations performed prior to the first dose of study drug and must meet all inclusion and exclusion criteria, as below.

Inclusion criteria

1) Age ≥ 18 years.
2) Life expectancy of at least 12 weeks (3 months).
3) Subjects must be able to understand and be willing to sign the written informed consent form. A signed informed consent form must be appropriately obtained prior to the conduct of any trial-specific procedure.
4) Histologically confirmed diagnosis of adenocarcinoma of the prostate. Histologic variants of prostate cancer, including neuroendocrine features are permitted; however, pure small cell carcinoma of the prostate is excluded.
5) Presence of >2 sites of metastatic disease in bone as determined by bone scan or CT, and for men who opt-in for bone biopsy, they must have at least one site amenable to radiographically-guided metastatic biopsy as determined by the study radiologist.
6) Symptomatic castration-resistant bone metastatic disease as determined by the provider.
7) Prior or concurrent therapy with either abiraterone acetate or enzalutamide.
8) Ongoing ADT using an LHRH agonist (e.g. leuprolide, goserelin) or antagonist (e.g. degarelix) must continue on therapy unless prior bilateral orchiectomy has been performed.
9) Current evidence of disease progression as evidenced by one of the following:
   a. 2 consecutive rising PSA levels separated at least 1 week apart above nadir PSA on last systemic therapy. If no nadir, then 2 rising PSA values greater than baseline pretreatment value is required from the most immediate prior therapy, **OR**
   b. CT or bone scan based evidence of disease progression with bone metastasis (new lesions or growth of existing lesions), **OR**
   c. Evidence of symptomatic progression (increased pain in an area with known lesions confirmed on imaging).
10) All acute toxic effects of any prior treatment have resolved to NCI-CTCAE v4.0 Grade 2 or less.
11) Men of childbearing potential must agree to use adequate contraception beginning at the signing of the ICF until at least 30 days after the last dose of study drug. The definition of adequate contraception will be based on the judgment of the principal investigator or a designated associate.

12) Acceptable hematology and serum biochemistry screening values:
    a. White Blood Cell Count (WBC) ≥ 3,000/mm3
    b. Absolute Neutrophil Count (ANC) ≥ 1,500/mm3
    c. Platelet (PLT) count ≥ 100,000/mm3
    d. Hemoglobin (HGB) ≥ 9.0 g/dl
    e. Total bilirubin level ≤ 1.5 x institutional upper limit of normal (ULN)
    f. Aspartate aminotransferase (AST) and alanine aminotransferase (ALT) ≤ 2.5 x ULN
    g. Creatinine ≤ 1.5 x ULN
    h. Albumin > 2.5 g/dL
13) Willing and able to comply with the protocol, including follow-up visits and examinations

<u>Exclusion criteria</u>

1) Treatment with cytotoxic chemotherapy within previous 4 weeks, or failure to recover from AEs down to grade 2 or less due to cytotoxic chemotherapy administered more than 4 weeks previous (however, ongoing neuropathy is permitted)
2) Receiving concurrent systemic therapy with radionuclides (e.g., strontium-89, samarium-153, rhenium-186, or rhenium-188) for the treatment of bony metastases. Prior therapy with radium-223 is not permitted.
3) Other malignancy treated within the last 3 years (except non-melanoma skin cancer or low-grade superficial bladder cancer)
4) Visceral (i.e. liver, lung, etc) metastases (pulmonary nodules ≤1cm are permitted) as assessed by chest, abdominal or pelvic computed tomography (CT) (or other imaging modality)
5) Presence of active untreated CNS parenchymal or epidural spinal metastases
6) Lymphadenopathy exceeding 3 cm in short-axis diameter
7) Imminent spinal cord compression based on clinical findings and/or magnetic resonance imaging (MRI). Treatment should be completed for spinal cord compression.
8) Any other serious illness or medical condition, such as but not limited to:
    a. Any infection ≥ National Cancer Institute Common Terminology Criteria for Adverse Events (NCI-CTCAE) version 4.0 Grade 2
    b. Cardiac failure New York Heart Association (NYHA) III or IV
    c. Crohn’s disease or ulcerative colitis
    d. Bone marrow dysplasia
    e. Fecal incontinence
9) Inability to comply with the protocol and/or not willing or not available for follow-up assessments.
10) Any condition which, in the investigator’s opinion, makes the subject unsuitable for trial participation.
11) Concurrent cytotoxic chemotherapy or anticancer therapies other than abiraterone, prednisone or other glucocorticoids, enzalutamide, androgen deprivation therapy, bisphosphonates, and denosumab.
12) Concurrent use of another investigational drug or device therapy (i.e., outside of study treatment) during, or within 2 weeks of treatment initiation.

13) Major surgery within 30 days prior to start of study drug.

Subject Recruitment and Compensation: This study will be open to members of all demographic groups who meet the eligibility criteria. Subjects will be recruited from the clinics at the Duke Cancer Center. A caregiver known to the patient will introduce the study and if the patient is interested, a member of the study team can approach for enrollment. Patients will not be enrolled without prior approval of their physician (if not a member of the study team). We will need to review protected health information such as name, history number, appointment date and physician, past cancer history, other past medical history and other protected health information (PHI) to determine study eligibility. This activity is used only to identify subjects, and information resulting from this activity will be used only to assess eligibility of the subject. It will not be recorded in case report forms (CRFs), and it will not leave Duke University Health System (DUHS). However, once the subject has been assigned to the study and consent has been obtained, documentation of eligibility will be recorded. Subjects will not receive compensation but will be given parking vouchers for study visits.

Consent Process: Subjects will be asked about participation by their Duke Oncologist during a regular clinic visit. If the subject is interested, a research oncology nurse will review the consent form with them in a private area and answer any questions the subject might have. This process will take approximately 30 minutes. The consent form will be given to the subject and they will be asked to take it home to review and discuss with family or friends as they desire.

Subject's Capacity to Give Legally Effective Consent: Subjects who do not have capacity to give legally effective consent will not be enrolled.

Study Interventions: Radium-223 will be administered per standard practice at dose of 50 kBq/kg (55kBq/kg upon implementation of the NIST update) q 4weeks x up to 6 doses. Before administration of study drug, the patient must be well hydrated; the patient should be instructed to drink ad libitum. Aseptic technique should be used in the administration of Radium-223 dichloride. The syringe should be handed over to the individual who will perform the injection. The radium-223 will be administered as a bolus intravenous (IV) injection (up to 1 minute).

Study Assessment: Vital signs, performance status, and physical exam will be assessed at each visit. The following laboratory studies will be obtained at intervals specified in the study flow chart to assess subject safety, specifically the risk of infection, bone marrow, liver, and kidney abnormalities: complete blood count, blood urea nitrogen (BUN), creatinine, sodium, potassium, calcium, chloride, carbon dioxide, phosphorus (optional), glucose, albumin, total protein, total bilirubin, alkaline phosphatase, AST (SGOT) and ALT (SGPT). A CBC with differential is required within 2 weeks of each radium-223 dose in order to determine whether subsequent dosing can proceed.

Optional bone metastasis biopsy will be performed under radiologic guidance prior to radium-223 initiation, following informed consent and determination of eligibility and registration. The second optional bone biopsy will be obtained at 15-20 days after the cycle 3 radium-223 dose is given. Bone biopsies will only be performed after informed consent and in locations considered to be low risk by the study team and interventional radiologist and will only target pelvic bone metastases. The third **optional** bone biopsy will be 15-25 days after the cycle 6 radium-223 treatment or on disease progression. Fresh bone biopsy sample, formalin-fixed paraffin- embedded (FFPE) and frozen (OCT) samples will be collected at each time for correlative studies. IHC staining will be done on FFPE sample for expression of PSA, CK, ALP, O-cadherin, N-cadherin, vimentin, TWIST, SNAIL, beta-catenin, ZEB1, phosphohistone H2AX (Ser139) (γ-H2AX); and FISH will be done for androgen receptor (AR) expression and AR variants. OCT sample will be used for DNA and RNA isolation to proceed to aCGH (array comparative genomic hybridization), WES (whole exome sequencing) and RNA sequencing. Fresh bone biopsy sample will be used to evaluate radium-223 decay products. The second and third bone metastasis biopsies will also be compared with baseline biopsy for changes in apoptosis (TUNEL, cleaved caspase-3 expression), reduction in proliferation (Ki-67), the abundance of tumor cells (CK + or PSA + cells in bone). Bone metastasis will be sampled at the edge of metastatic lesion and at adjacent bone approximately 1cm distant to the metastatic lesion to survey the extent of physiologic impact of radium-223 and describe the changes in apoptosis (TUNEL, cleaved caspase-3 expression) and proliferation (Ki-67) regionally. Bone metastatic tumor tissue will also be used to evaluate the final decay

products of Ra-223 using radiochemistry assays by a member of our team in nuclear medicine and radiochemistry. **Further details for each assay/biomarker are provided in the laboratory manual.**

For efficacy assessment, standard-of-care biomarkers including PSA and lactate dehydrogenase (LDH) will be drawn prior to initiation (PSA only), month 1 (LDH only), month 3, month 6 (LDH only), and on progression. Research Cellsearch CTC tests for correlative studies will also be drawn at screening, month 1, month 3, month 6, and at progression: 7.5 mL Cellsave tubes (2) will be collected at each time point starting on day 1. In addition, five 10 mL EDTA tubes will be collected at the same time points for flow cytometric analysis. Captured CTCs from both CellSearch and flow cytometry will be examined for cytokeratin, ALP, PSA, PSMA expression by IHC, and AR amplification by FISH. Additional peripheral blood (total 15 ml) will be obtained prior to initiation of radium-223 for plasma isolation and whole blood RNA and plasma DNA extraction. Each whole blood RNA isolation will be duplicated by using two paxgene tubes. Extracted RNA will then be processed for RNA sequencing. And extracted peripheral blood plasma will be used for DNA isolation and target sequencing.

Standard of care CT scans with contrast of the chest, abdomen, and pelvis and a nuclear bone scan **or** a sodium-fluoride PET-CT (F-18) will be performed within 30 days prior to registration and on progression. F-18 NaF PET/CTs will only be performed when available through the separate Medicare NOPR registry.

Safety: Subjects receiving at least one dose of radium-223 will be evaluable for safety. Safety will be assessed by physical exam, laboratory assessments, review of concomitant medications, adverse event (AE) and serious adverse event (SAE) evaluations every cycle throughout the study. NCI Common Toxicity Criteria (v 4.0) (http://evs.nci.nih.gov/ftp1/CTCAE/About.html) will be used to record and monitor for adverse events. Treatment will be held for certain specified grade 3 or 4 adverse events until resolution to grade 1 as specified in the protocol.

Costs to the Subject: Radium-223 is considered standard of care for this study and is being used as per the USFDA label and will be paid by a subject's insurance. Research procedures covered by the study include research blood tests and bone biopsies. There are no additional costs to patients to be involved into the study.

Statistical Considerations: This is the first study to estimate the proportion of patients who will over-express ALP and other osteomimicry biomarkers in the CTCs and as such the prevalence of patients who are expected to over-express ALP in CTCs is unknown. It is though anticipated that at least 50% of men will over-express ALP in their circulating tumor cells. Alkaline phosphatase expression will be concurrently examined in the bone metastatic biopsies of men as well, if evaluable tissue is available. With 20 patients enrolled in the study, the proportion of patients who will over-express alkaline phosphatase in their CTCs (defined as any over-expression) can be estimated with a standard error of no greater than 11.2 percentage points. Descriptive statistics will be applied to the proportion of men expressing ALP at each time point (pre and post-treatment) and for each osteomimicry/EMT biomarker in bone/tissue and CTCs at each time point, as well as within the geographic distribution of marker expression within each metastatic biopsy (central vs periphery).

Data Analysis: This is a pilot correlative science study. For the primary objective, the proportion of patients who will over-express ALP in circulating tumor cells (total expression divided by total number of CTCs by FACS/imagestream analysis) will be estimated and the exact 95% confidence intervals based on the binomial distribution will be computed. CTCs will be defined as CD45 negative, nucleated, intact cells, and will be further characterized by expression of EpCAM, cytokeratin, and alkaline phosphatase based on control cell lines. The expression levels of the biomarkers will be summarized using descriptive statistics (means, SE, median, and inter-quartile ranges) at baseline and over time, with 95% confidence intervals around these estimates. Scatterplots and waterplots for changes from baseline for all markers at the indicated time point will be used. The Pearson's correlation coefficient and 95% confidence interval will be calculated for each paired markers. The Kaplan-Meier product limit method will be used to estimate the PFS and OS distributions. While it is expected that this pilot study with a small sample size to be exploratory, the estimates will be useful in the planning of larger trials and in exploring the relevance of osteomimicry to bone metastatic biology and radium-223 efficacy.

Data and Safety Monitoring: Data for safety and severe adverse events will be monitored on an ongoing basis through monthly investigator and staff meetings. Given the standard of care nature of the treatment, no early stopping rules are necessary for this study. The sponsor is responsible to comply with the local regulation and legislation for adverse events reporting. All observations pertinent to the safety of the study treatment will be recorded and included in the final report. AEs will be reported as described in section 9. For all events, the relationship to treatment and the intensity of the event will be determined by the investigator. Data will be collected using electronic case report forms.

Privacy, Data Storage, and Confidentiality: To the extent permitted by law and by signing the consent form, subjects allow access for representatives of the FDA, other national regulatory authorities, the Institutional Review Board, and sponsor monitors/representatives and collaborators to inspect the research and clinical records without removal of identifying information, such as name, initials, date of birth, sex, race, and location of the research study. If information from this study is presented publicly or published in a medical journal, subjects will not be identified by name, picture, or any other personally identifying information.

3 STUDY SCHEMA

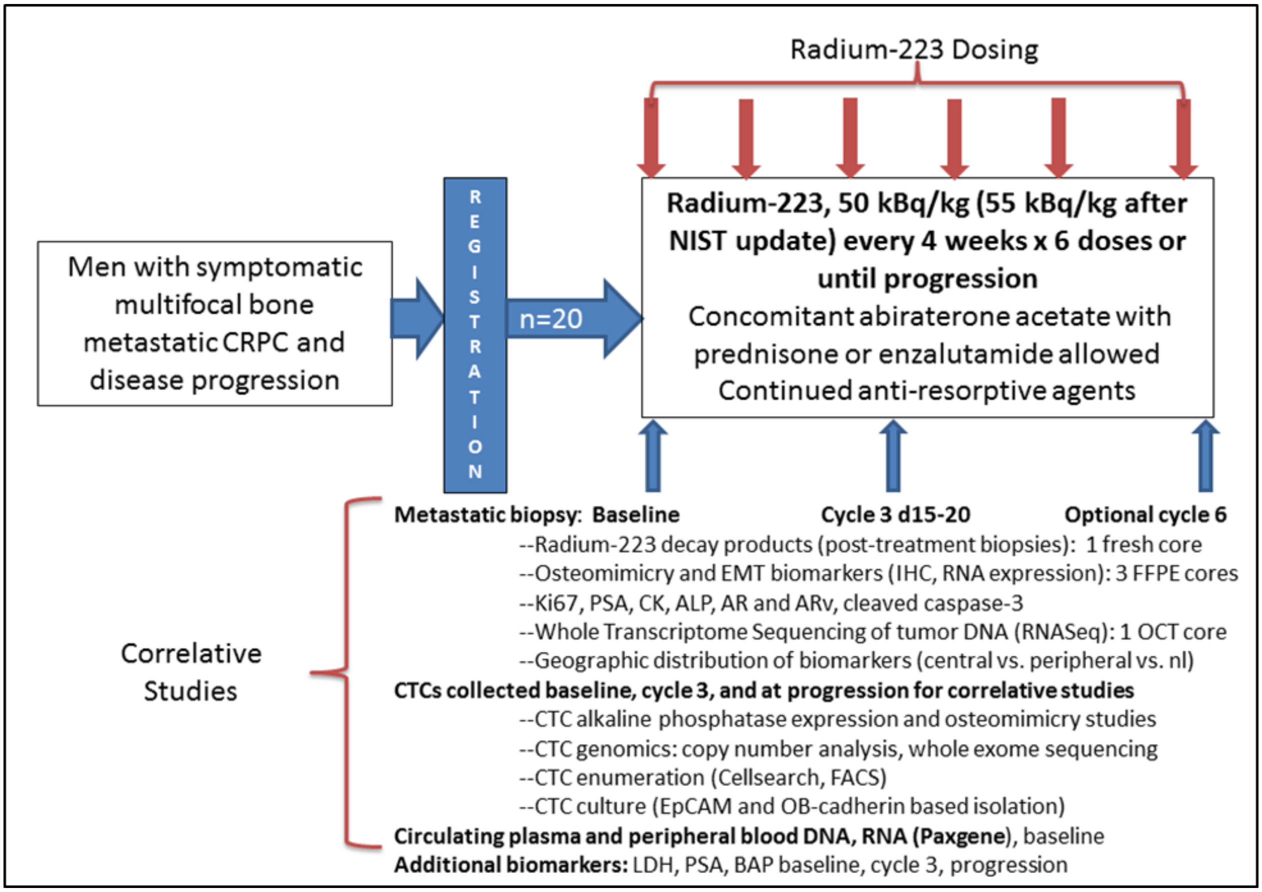

4 BACKGROUND AND SIGNIFICANCE

4.1 Study Disease

Prostate cancer is the most prevalent non-skin cancer in men of America, with almost 200,000 cases diagnosed in 2009. Although the clinical course is heterogeneous, metastatic prostate cancer is incurable and is the second leading cause of cancer death in the U.S.(Jemal, 2009). Medical or surgical castration is the mainstay of therapy for metastatic prostate cancer; however, response is usually temporary and the disease eventually escapes hormonal control. Castration resistant prostate cancer (CRPC) is defined by disease progression despite androgen deprivation and castrate levels of testosterone. Despite significant advances in diagnosis and management, prostate cancer is still a leading cause of cancer-related death in men and at this time the median survival for castration-resistant metastatic prostate cancer (CRPC) ranges between approximately 1-2 years depending on known prognostic factors. There have been a rapidly increasing number of new systematic agents have been approved by the USFDA for men with metastatic CRPC, based on the results of successful phase 3 trials of a diverse range of agents with immunomodulatory, hormonal, bone-targeting, and microtubule targeting mechanisms of action.

Over the past year, significant progress has been made in expanding the treatment options for patients with metastatic CRPC. Men with metastatic CRPC in 2013 have an improved prognosis based on therapeutic advances in immunotherapy (sipuleucel-T), hormonal therapies (abiraterone and enzalutamide), chemotherapy (docetaxel,

cabazitaxel), and bone targeting radium-223. Currently, however, few predictive biomarkers exist to guide the rational selection or sequencing of these systemic therapies. Prediction of benefit with a systemic therapy is of great importance, as knowledge of a biomarker is tied directly to a therapeutic implication and greater potential to maximize benefit and minimize harms and costs.

Radium-223 has demonstrated improved overall survival (OS) in men with symptomatic bone-metastatic CRPC in the post-docetaxel and docetaxel ineligible population (ALSYMPCA trial) (Nilsson, 2013; Parker, 2013). In this analysis, it was found that the baseline osteoblast activity biomarker alkaline phosphatase (ALP) was prognostic (men with high alkaline phosphatase had shorter survival), similar to what has been has been reported in multiple datasets of men with mCRPC. In addition, there was a suggestion of predictive role for baseline ALP in that men with higher baseline ALP had a substantial and statistically significant benefit with radium-223 over placebo plus support care, while men with normal/low baseline ALP had no clear clinical or statistical benefit. ALP has long been known to be prognostic for survival in men with metastatic cancer and improvements in ALP have been associated with improvements in survival after the initiation of hormonal therapy and docetaxel chemotherapy (Sonpavde, 2010). The findings in ALSYMPCA, however, suggest that elevations in osteoblast activity as measured by serum ALP, may be linked to greater uptake and pharmacodynamics effect of radium-223 in bone, and may be predictive of the benefits of this drug for improved OS. These findings have mechanistic support, biologic plausibility, and offer compelling evidence that may improve the efficacy and delivery and reduce the toxicity and costs of radium-223 in the clinic.

However, it is currently unclear what the source of the ALP elevations are in men with bone-metastatic CRPC. Under certain conditions, prostate cancer cells have been shown to undergo osteoblastic differentiation and osteomimicry and may ALP (Lin, 2001; Knerr, Int J Cancer 2004; Zhau, 2011). This osteomimicry hypothesis was first developed in 1999 and has largely been confined to date to preclinical and in vitro models rather than in patients (Koeneman, 1999). We have demonstrated that the circulating tumor cells from men with predominantly bone metastatic mCRPC commonly express the osteomimicry biomarker O-cadherin (70-80% expression) (Armstrong, 2011). O-cadherin has been shown to be induced through epithelial-mesenchymal transitions (EMT) and loss of E-cadherin expression, and promotes homing to bone, while experimental knockdown of O-cadherin reduces bone metastases in preclinical prostate cancer models (Chu, 2008). O-cadherin expression has additionally been shown to be expressed in the bone metastases of men with mCRPC, but not in the metastases of other organ sites, suggesting that a tumor-bone microenvironment cross talk may lead to epithelial plasticity and osteomimicry. These findings have therapeutic implications, given that bone-targeting agents such as radium-223 may have an ability to be taken up directly by bone forming prostate cancer cells rather than simply by cells in the tumor microenvironment.

Based on these findings, and that biomarkers of such osteomimicry may predict for the benefit of radium-223, the discovery of the bone forming ability of prostate cancer cells will provide evidence for the selective targeting of radium-223 to the osteoblastic tumor. We propose that prostate cancer cells (both tissue and CTCs) from men with bone metastatic CRPC will commonly express markers of EMT and osteomimicry through a biologic process termed epithelial plasticity, and that understanding this process will help direct the therapeutic potential of radium-223. Direct visualization of co-expression of epithelial markers with bone markers in metastases and CTCs will provide evidence for the osteomimicry hypothesis and the potential for radium-223 to target the metastatic tumor directly. In addition, the evaluation and visualization of the radium-223 localization and its decay products in bone metastatic lesions and the surrounding micro and macroenvironment will provide insights into the target (tumor, host, or both) of this radiopharmaceutical.

4.2 Study Agent: Radium-223 dichloride

4.2.1 Description

Radium-223 dichloride, an alpha particle-emitting pharmaceutical, is a radiotherapeutic drug. Radium-223 dichloride is supplied as a clear, colorless, isotonic, and sterile solution to be administered intravenously with pH between 6 and 8. The product is supplied in single-dose glass vials with a radioactivity concentration of 1000

kBq/mL (0.03 mCi) at the reference date (1100 kBq/mL; 0.03 mCi after the NIST implementation date). Radium is present in the solution as a free divalent cation. The volume per vial is 6 mL, corresponding to 6 MBq (6.6 MBq after implementation of NIST update) at the reference date.The inactive ingredients are 6.3 mg/mL sodium chloride USP (tonicity agent), 7.2 mg/mL sodium citrate USP (for pH adjustment), 0.2 mg/mL hydrochloric acid USP (for pH adjustment), and water for injection USP.

The molecular weight of radium-223 dichloride, 223RaCl2, is 293.9 g/mol. Radium-223 has a half-life of 11.4 days. The specific activity of radium-223 is 1.9 MBq (51.4 microcurie)/ng (2.1 MBq (56.8 microcurie) after the NIST update). The six-stage-decay of radium-223 to stable lead-207 occurs via short-lived daughters, and is accompanied predominantly by alpha emissions. There are also beta and gamma emissions with different energies and emission probabilities. The fraction of energy emitted from radium-223 and its daughters as alpha-particles is 95.3% (energy range of 5 - 7.5 MeV). The fraction emitted as beta-particles is 3.6% (average energies are 0.445 MeV and 0.492 MeV), and the fraction emitted as gamma-radiation is 1.1% (energy range of 0.01 - 1.27 MeV).

4.2.2 National Institute of Standards and Technology (NIST) Standardization

The quantification of radium-223 radioactivity in Xofigo (radium-223 dichloride;BAY 88-8223) is based on the primary standardization performed by the US NIST. National Institute of Standards and Technology prepares the standard reference material (SRM) using an official dial setting (primary standardization) as published (Cessna, 2010). The NIST SRM is used to calibrate the instruments in production and quality control for both the drug substance and drug product. Additionally, the NIST SRM is used to prepare the NIST traceable Ra-223 reference materials which are then sent to the end-users (e.g., nuclear medicine laboratory physicians or technicians) for dial-setting of their dose calibrators, to allow verification of the patient dose.

In 2014, NIST performed a re-assessment of the primary standardization based on preliminary information suggesting a potential discrepancy of approximately 8-10% between the published NIST primary standardization (Cessna, 2010) and results obtained by other national metrology institutes (United Kingdom, Germany, Japan). After completion of the re-assessment, NIST reported their findings (Zimmerman, 2015) and had issued a revised NIST SRM in 2015. The discrepancy in the NIST standardization was determined to be −9.5% between activity values obtained using the old reference standard relative to the new primary standardization. Consequently the current numerical values need to be corrected by approx. + 10.5%.

The current NIST standard for radium-223 dichloride will remain in effect until the FDA has fully approved the regulatory variation submitted for Xofigo and is anticipated in the 2nd quarter of 2016. All sites are expected to begin preparation for the updated NIST standardization and obtain all necessary IRB approvals. Bayer will continue to notify sites about the status of the regulatory approval and the date that the updated NIST standardization is to be implemented. Upon notification, and prior to the implementation, all sites are expected to add a new dial setting to their dose calibrators for the new NIST standardization for radium-223 dichloride, which should be documented on the appropriate study forms. The change in the numerical description of the patient's dose, product strength and labeled vial activity does not impact the safety or efficacy of Xofigo. The change in the NIST radium-223 standard has no impact on subjects; dose subjects are receiving, and will continue to receive. Subjects will receive the same actual dose and volume that was studied in Study 15245 (BC1-06 dosimetry study) and is associated with the proven safety and efficacy of radium-223 dichloride, though the stated nominal radiation dose received is being updated to reflect the new standard. The formula for the calculation of the volume to be administered has to be changed respectively. (see dosing section)

Subjects who are on-treatment at the time the new NIST reference standard goes into effect should be notified of this change and should be required to sign a Patient Information Sheet to acknowledge that they have received information on the updated NIST standard calibration. All patients enrolled after the new reference standard is in effect should sign a revised Informed Consent Form that contains the updated NIST standardization.

4.2.3 Mechanism of Action

The active moiety of Radium-223 dichloride is the alpha particle-emitting isotope radium-223, which mimics calcium and forms complexes with the bone mineral hydroxyapatite at areas of increased bone turnover, such as bone metastases. The high linear energy transfer of alpha emitters (80 keV/micrometer) leads to a high frequency of double-strand DNA breaks in adjacent cells, resulting in an anti-tumor effect on bone metastases. The alpha particle range from radium-223 dichloride is less than 100 micrometers (less than 10 cell diameters) which limits damage to the surrounding normal tissue.

4.2.4 Pre-clinical experience

In single and repeated dose toxicity studies in rats, the main findings were reduced body weight gain, hematological changes, reduced serum alkaline phosphatase and microscopic findings in the bone marrow (depletion of hematopoietic cells, fibrosis), spleen (secondary extra-medullary hematopoiesis) and bone (depletion of osteocytes, osteoblasts, osteoclasts, fibro-osseous lesions, disruption/disorganization of the physis/growth line).

These findings were related to radiation-induced impairment of hematopoiesis and a reduction of osteogenesis and occurred beginning in the dose range of 20 (0.00056 mCi) – 80 kBq (0.0022 mCi) per kg body weight, with the exception of body weight decreases.

Dose-limiting myelotoxicity was seen in dogs after single administration of 450 kBq (0.012 mCi) Radium-223 dichloride per kg body weight (9 times the clinically recommended dose).

Osteosarcomas, a known effect of bone-seeking radionuclides, were observed at clinically relevant doses in rats 7 – 12 months after start of treatment. Osteosarcomas were not observed in dog studies. The presence of neoplastic changes, other than osteosarcomas, was also reported in the longer term (12 to 15 months) rat toxicity studies. Due to its mode of action, and as seen with conventional radiotherapy and other radiotherapeutics, radium-223 dichloride may have the potential to induce secondary malignancies. No case of osteosarcoma has been reported in clinical studies with Radium-223 dichloride. The risk for patients to develop osteosarcomas with exposure to Radium-223 dichloride is unknown at present

Studies on reproductive and developmental toxicity have not been performed. Since Radium-223 dichloride binds to bone, the potential risk for toxic effects in the male gonads in cancer patients with castration-resistant prostate cancer is very low, but cannot be excluded. Studies on the mutagenic and carcinogenic potential of Radium-223 dichloride have not been performed.

No histological changes were observed in organs involved in the excretion of Radium-223 dichloride. No significant effects were seen on vital organ systems, i.e. cardiovascular (dog), respiratory or central nervous systems (rat), after single dose administration of 450 to 1000 kBq (497 kBq- 1100kBq after implementation of NIST update), (per kg body weight (9 (dog) to 20 (rat) times the clinically recommended dose.

4.2.5 Clinical experience

The clinical development of Radium-223 dichloride includes phase I and phase II studies in prostate cancer patients with bone metastases. The results of these completed studies indicated that safety and tolerability of Radium-223 dichloride in CRPC/HRPC patients with bone metastases was well tolerated, and that there was evidence of dose related efficacy against bone markers and other markers of disease. In addition there was an effect on median overall survival in a Phase II (BC1-02) placebo-controlled study. These studies enabled the initiation of the Phase III ALSYMPCA (ALpharadin in SYMptomatic Prostate CAncer) study.

The clinical safety and efficacy of Radium-223 dichloride have been evaluated in a double-blind, randomized, multiple dose, phase III multicenter study (ALSYMPCA) in castration-resistant prostate cancer patients with bone metastases. The primary efficacy endpoint was Overall Survival (OS).

At the cut-off date of the pre-planned interim analysis, a total of 809 patients were randomized 2:1 to receive Radium-223 dichloride 50 kBq kg (corresponding to 55kBq/kg after implementation of the NIST) intravenously every 4 weeks for 6 cycles (N=541) plus best standard of care or matching placebo plus best standard of care (N=268). Best standard of care included e.g. local external beam radiotherapy, corticosteroids, antiandrogens, estrogens, estramustine or ketoconazole.

An updated descriptive analysis of safety and of OS was performed in 921 randomized patients prior to implementing crossover (i.e. offering patients in the placebo group to receive Radium-223 dichloride treatment).

The results of both, interim and updated analysis, revealed that OS was significantly longer in patients treated with Radium-223 dichloride plus best standard of care compared to patients treated with placebo plus best standard of care. For the updated analysis, an increase in median overall survival of 3.6 months was seen with Radium-223 dichloride plus best standard of care compared to placebo plus best standard of care (HR =0.695 (95% CI 0.581/0.832), median OS 14.9 months versus 11.3 months, respectively).

In the ALSYMPCA study, the results of the interim analysis and the updated analysis showed also a significant improvement in all main secondary endpoints in the Radium-223 dichloride arm compared to the placebo arm:

- Time to first SRE (defined as time to EBRT, time to first pathological bone fracture, time to spinal cord compression and time to surgical intervention) was statistically significantly longer in the radium-223 chloride group compared to placebo (median number of months=15.6 for radium-223 chloride versus 9.8 months for placebo (HR=0.658, 95 CI 0.522–0.830, p= 0.00037).
- Time to total ALP progression (defined as ≥ 25% increase compared to baseline/nadir) was statistically significantly longer in the radium-223 chloride group 7.4 months compared to placebo 3.8 months (HR = 0.167, 95% CI 0.129 – 0.217; p= <0.00001).
- Time to PSA progression (defined as a ≥ 25% increase and an increase in absolute value of ≥ 2 ng/mL compared to baseline/nadir) was also significantly prolonged in patients receiving Radium-223 dichloride compared to patients receiving placebo (HR = 0.643, 95% CI 0.539,0.768; p = <0.00001)
- A total ALP response (defined as a confirmed ≥ 30% or ≥ 50% reduction compared to baseline) at week 12 was observed in higher proportions of subjects who were treated with radium-223 chloride group (47% and 3% respectively) compared to those in the placebo (3% and <1% respectively) group.

Subgroup survival analysis showed a consistent survival benefit for treatment with Radium-223 dichloride, independent of total alkaline phosphatase (ALP), current use of bisphosphonates, prior use of docetaxel and baseline ECOG status. The results from the phase III ALSYMPCA study regarding time to external beam radiation therapy (EBRT) for pain relief and fewer patients reporting bone pain as an adverse event in the Radium-223 dichloride group indicate a positive effect on bone pain.

The most common adverse reactions (≥ 10%) in patients receiving Radium-223 dichloride were nausea, diarrhea, vomiting, and peripheral edema (Table 3). Grade 3 and 4 adverse events were reported among 57% of Radium-223 dichloride-treated patients and 63% of placebo treated patients. The most common hematologic laboratory abnormalities in Radium-223 dichloride-treated patients (≥ 10%) were anemia, lymphocytopenia, leukopenia, thrombocytopenia, and neutropenia (Table 2). Treatment discontinuations due to adverse events occurred in 17% of patients who received Radium-223 dichloride and 21% of patients who received placebo.

The most common hematologic laboratory abnormalities leading to discontinuation for Radium-223 dichloride were anemia (2%) and thrombocytopenia (2%).

Table 1 shows adverse reactions occurring in ≥ 2% of patients and for which the incidence for Radium-223 dichloride exceeds the incidence for placebo.

Table 1 Adverse Reactions in the Randomized Trial

System/Organ Class Preferred Term	Radium-223 dichloride (n=600)		Placebo (n=301)	
	Grades 1-4 %	Grades 3-4 %	Grades 1-4 %	Grades 3-4 %
**Blood and lymphatic system disorders**				
Pancytopenia	2	1	0	0
**Gastrointestinal disorders**				
Nausea	36	2	35	2
Diarrhea	25	2	15	2
Vomiting	19	2	14	2
**General disorders and administration site conditions**				
Peripheral edema	13	2	10	1
**Renal and urinary disorders**				
Renal failure and impairment	3	1	1	1

Laboratory Abnormalities

Table 2 shows hematologic laboratory abnormalities occurring in > 10% of patients and for which the incidence for Radium-223 dichloride exceeds the incidence for placebo.

Table 2: Hematologic Laboratory Abnormalities

Hematologic Laboratory Abnormalities	Xofigo (n=600)		Placebo (n=301)	
	Grades 1-4 %	Grades 3-4 %	Grades 1-4 %	Grades 3-4 %
Anemia	93	6	88	6
Lymphocytopenia	72	20	53	7
Leukopenia	35	3	10	<1
Thrombocytopenia	31	3	22	<1
Neutropenia	18	2	5	<1

Laboratory values were obtained at baseline and prior to each 4-week cycle.

As an adverse reaction, grade 3-4 thrombocytopenia was reported in 6% of patients on Radium-223 dichloride and in 2% of patients on placebo. Among patients who received Radium-223 dichloride, the laboratory abnormality grade 3-4 thrombocytopenia occurred in 1% of docetaxel naïve patients and in 4% of patients who had received prior docetaxel. Grade 3-4 neutropenia occurred in 1% of docetaxel naïve patients and in 3% of patients who have received prior docetaxel.

Fluid Status

Dehydration occurred in 3% of patients on Radium-223 dichloride and 1% of patients on placebo. Radium-223 dichloride increases adverse reactions such as diarrhea, nausea, and vomiting which may result in dehydration. Monitor patients' oral intake and fluid status carefully and promptly treat patients who display signs or symptoms of dehydration or hypovolemia.

Injection Site Reactions

Erythema, pain, and edema at the injection site were reported in 1% of patients on Radium-223 dichloride.

Secondary malignant neoplasms
No cases of radiation-induced cancer have been reported in reported in clinical trials with radium-223 dichloride in follow-up of up to three years. However, the radiation dose resulting from therapeutic exposure may result in higher incidence of cancer (e.g. sarcomas of the bone, or leukemia), mutations and a potential for development of hereditary defects.

Bone Marrow Suppression
In the randomized trial, 2% of patients on the Radium-223 dichloride arm experienced bone marrow failure or ongoing pancytopenia compared to no patients treated with placebo. There were two deaths due to bone marrow failure and for 7 of 13 patients treated with Radium-223 dichloride, bone marrow failure was ongoing at the time of death. Among the 13 patients who experienced bone marrow failure, 54% required blood transfusions. Four percent (4%) of patients on the Radium-223 dichloride arm and 2% on the placebo arm permanently discontinued therapy due to bone marrow suppression.

In the randomized trial, deaths related to vascular hemorrhage in association with myelosuppression were observed in 1% of Radium-223 dichloride-treated patients compared to 0.3% of patients treated with placebo. The incidence of infection-related deaths (2%), serious infections (10%), and febrile neutropenia (<1%) were similar for patients treated with Radium-223 dichloride and placebo.

Myelosuppression; notably thrombocytopenia, neutropenia, pancytopenia, and leukopenia; has been reported in patients treated with Radium-223 dichloride. In the randomized trial, complete blood counts (CBCs) were obtained every 4 weeks prior to each dose and the nadir CBCs and times of recovery were not well characterized. In a separate single-dose phase 1 study of Radium-223 dichloride, neutrophil and platelet count nadirs occurred 2 to 3 weeks after Radium-223 dichloride administration at doses that were up to 1 to 5 times the recommended dose, and most patients recovered approximately 6 to 8 weeks after administration

Hematologic evaluation of patients must be performed at baseline and prior to every dose of Radium-223 dichloride. Before the first administration of Radium-223 dichloride, the absolute neutrophil count (ANC) should be ≥ 1.5 x 109/L, the platelet count ≥ 100 x 109/L and hemoglobin ≥ 9.0 g/dL. Before subsequent administrations of Radium-223 dichloride, the ANC should be ≥ 1 x 109/L and the platelet count ≥ 50 x 109/L. If there is no recovery to these values within 6 to 8 weeks after the last administration of Radium-223 dichloride, despite receiving supportive care, further treatment with Radium-223 dichloride should be discontinued. Patients with evidence of compromised bone marrow reserve should be monitored closely and provided with supportive care measures whenclinically indicated. Discontinue Radium-223 dichloride in patients who experience life-threatening complications despite supportive care for bone marrow failure.

The safety and efficacy of concomitant chemotherapy with Radium-223 dichloride have not been established. Outside of a clinical trial, concomitant use with chemotherapy is not recommended due to the potential for additive myelosuppression. If chemotherapy, other systemic radioisotopes or hemibody external radiotherapy are administered during the treatment period, Radium-223 dichloride should be discontinued.

4.2.6 Rationale for US National Institute of Standards and Technology (NIST) Update

The quantification of radium-223 radioactivity in Xofigo® is based on the primary standardization performed by the US National Institute of Standards and Technology (NIST). The NIST Standard Reference Material is used to calibrate the instruments in production and quality control of both the drug substance and drug product. Additionally, the calibrated instruments in production at the Institute for Energy Technology (IFE, Norway) are used to prepare the NIST traceable radium-223 reference material, which are then sent to the treatment sites (e.g., nuclear medicine laboratory physicians or technicians) for dial setting of their dose calibrators, to allow verification of the patient dose. A reassessment of the primary standardization was initiated by the NIST. A discrepancy of approximately 10% between the published NIST primary standardization (Cessna, 2010, NIST 2010)

and current measurements was confirmed and a revised NIST primary reference standard has been issued (Zimmerman, 2015, NIST update). As a result of the revised NIST primary standardization, an adaption of the numerical description of patient dose and the description of radioactive concentration of the drug product solution becomes necessary. This concerns Xofigo® for commercial use and product used in clinical trials.

After the implementation of the new standard (NIST update) the numerical description of the patient dose will be adjusted from 50 kBq/kg to 55 kBq/kg, and the numerical description of the radioactivity in the vial will be changed from 1,000 kBq/mL to 1,100 kBq/mL. The values in this protocol have been revised as per the United States National Institute of Standards and Technology (NIST) standardization update.

Bayer has submitted a variation application to the FDA. The current standard (NIST 2010), dial setting and dose will remain in effect until Bayer has confirmed the unique implementation date in the 2nd quarter of 2016 as agreed with FDA and notified Duke University Medical Center.

4.3 Study Purpose/Rationale

The purpose of this study is to estimate ALP expression in CTCs and bone metastases and expression patterns of other biomarkers of osteomimicry in men with bone metastatic CRPC. We will also describe the molecular characteristics of bone metastases in men with bone metastatic prostate cancer and evidence for osteomimicry and epithelial plasticity (mesenchymal transitions) in the blood and tumor tissue. These findings may help elucidate the underlying mechanism of action of radium-223's benefit in men with bone metastases. We will evaluate radium-223 localization and its decay products in bone metastatic lesions and the surrounding micro and macroenvironment to provide insights into the target (tumor, host, or both) of this radiopharmaceutical.

5 OBJECTIVES AND ENDPOINTS

	Objective	Endpoint	Analysis
**Primary**<br><br><br><br>**Secondary**	Describe the proportion of patients who overexpress alkaline phosphatase (ALP) in CTCs at each time point.<br><br>Secondary objectives will look at biomarkers of epithelial plasticity and osteomimicry in the metastases of men with bone metastatic CRPC.	Proportion of patients who overexpress ALP in CTCs at each time point.<br><br>Secondary: summary statistics on the expression of alkaline phosphatase, PSA, CK, O-cadherin, N-cadherin, vimentin, TWIST, SNAIL, beta-catenin, ZEB1, androgen receptor (AR), AR variants (ARv) and γ-H2AX in bone biopsies of men with mCRPC	See Section 8.6
**Exploratory**	To evaluate the molecular characteristics of bone metastases and CTCs in men with mCRPC through DNA aCGH and RNA Sequencing, with a focus on pathways important in epithelial plasticity and bone formation.<br><br>To evaluate peripheral blood DNA and RNA expression profiling for evidence of biomarkers of epithelial plasticity.<br><br>To evaluate plasma DNA for evidence of biomarkers of drug resistance	Description of whole genomic DNA and RNA lesions in CTCs and bone metastases biopsy by DNA arrayCGH and RNA sequencing.<br><br>Whole exome sequencing and sequencing RNA obtained from peripheral blood.<br><br>Target sequencing of DNA extracted from plasma	See Section 8.6
**Exploratory**	To describe the anti-tumor effects over time during treatment with radium-223 based on sequential tumor metastatic biopsies as measured by changes in proliferation and induction of apoptosis	Detection of induced apoptosis and reduced proliferation markers in paired bone metastatic biopsies: TUNEL, cleaved caspase-3 expression, Ki-67, the abundance of tumor cells (CK + or PSA + cells in bone)	See Section 8.6
**Exploratory**	To evaluate the regional extent of the treatment effect of radium-223 in the central and peri-blastic regions of the bone metastases.	Biopsy at the edge of bone metastasis and adjacent bone tissue about 1cm distant to the metastasis lesion ; autoradiography of radium-223 and its decay products bound to hydroxyapatite in Ilford K5 emulsion; survey the extent of physiologic impact of radium-223 (apoptosis and proliferation as above) according to region of bone and bone metastasis biopsied	See Section 8.6
**Exploratory**	To measure the accumulation in bone metastases of stable daughter decay products (Pb-207 and possible others) at month 3 in men treated with radium-223	Measure the Pb207 in bone metastasis biopsy	See Section 8.6
**Exploratory**	To estimate progression free survival and overall survival	Time from registration to progression or death and overall survival time	See Section 8.6

6 INVESTIGATIONAL PLAN

6.1 Study Design

This will be a prospective, pharmacodynamic biomarker study of men with symptomatic multifocal bone metastatic CRPC, inclusive of men in the pre-docetaxel and post-docetaxel treatment space. Radium-223 will be given to all men, who will be followed until disease progression. Men will be permitted to be treated concurrently with standard of care hormonal therapies (androgen deprivation therapy and abiraterone acetate and prednisone or enzalutamide or other glucocorticoids). Eligible men must have disease progression (radiographic, symptomatic, or PSA based progression) at study entry and for those men who elect to have an optional bone biopsy, must also have a bone metastatic site amenable to a research bone biopsy under radiographic guidance. **This is a Duke-only, single site open-label investigator initiated trial.**

In this study, 20 men with bone metastatic CRPC and disease progression will be prospectively consented and enrolled from the Duke Cancer Institute prostate cancer clinic. See eligibility criteria, which briefly includes men over age 18 with >2 sites of bone metastatic disease, castration resistant progression on ADT based on PSA, bone/radiographic criteria, or clinical criteria such as pain). The study radiologist must approve the metastatic site as amenable to radiographically guided biopsy for all optional bone biopsies. Treatment with radium-223 as per standard practice (50 kBq/kg (55 kBq after the NIST update) q 4weeks x up to 6 doses), and concurrent abiraterone acetate/enzalutamide and ongoing ADT and bone anti-resorptive agents is permitted following registration and eligibility verification. A baseline optional CT guided bone metastatic biopsy prior to therapy will be performed for correlative studies as described below, and optional second and optional third bone metastatic biopsies will also be performed 15-20 days after the 3rd cycle of radium-223 treatment and 15-25 days after the 6th cycle of radium/or on progression, respectively.

Metastatic bone biopsies will be prospectively collected at baseline in these 20 men if they opt in for this procedure. All men will have CTCs collected over time for the primary endpoint, however. Tissue will be formalin fixed and evaluated by IHC and DNA and RNA extraction for aCGH, WES and RNA Sequencing. PSA and CK expression will be examined on the biopsy samples by IHC. ALP, O-cadherin, N-cadherin, vimentin, TWIST, SNAIL, beta-catenin, ZEB1 expression and γ-H2AX will also be evaluated by IHC as osteomimicry/EMT biomarker, which are known to promote epithelial plasticity. FISH will be explored to study androgen receptor (AR) and AR variants. Several core biopsies in addition will be collected and snap frozen for DNA and RNA extraction to proceed with RNA and DNA extraction for aCGH, WES and RNA sequencing to examine in an unbiased manner broad tumor genomic differences in men identified based on ALP levels. This correlative data will provide evidence as to the molecular characteristics of men with bone metastases and evidence for osteomimicry and epithelial plasticity (mesenchymal transitions) that may promote bone formation and thus targeting by radium-223. Given the relevance of ALP levels as a potential predictive biomarker for bone targeted agents such as radium-223, understanding the source (tumor vs. host) of ALP and bone formation is critical to selecting patients and understanding and improving therapeutic efficacy in the pre-docetaxel treatment space.

The optional second and third bone biopsies will explore the regional extent of radium-223 efficacy as determined by the physiologic response in the bone metastasis. This will be determined by changes in markers of apoptosis (TUNEL, cleaved caspase-3 expression), reduction in proliferation (Ki-67), the abundance of tumor cells (CK + or PSA + cells in bone) after cycle 1 radium-223 treatment and cycle 6/or on progression as compared to baseline. This is an exploratory objective and will provide descriptive data as to the physiologic impact of radium-223 in the bone metastasis. We will attempt to sample where feasible the bone metastasis at the edge of bone metastatic lesion and at adjacent bone about 1 cm distant to the bone metastatic lesion to survey the extent of physiologic impact of radium-223 and describe the changes in apoptosis and proliferation regionally. FFPE samples from the 2nd and 3rd bone metastasis biopsies will be utilized for autoradiography of radium-223 and its decay products which cooperate to hydroxyapatite in Ilford K5 emulsion. Finally, we will collect metastatic tumor tissue to evaluate the final decay product of Ra-223 in bone using assays by a member of our team in nuclear medicine (stable Pb-207) (Vaidyanathan, 2011). Localization of lead decay products in the tumor cells, surrounding stroma and more distant normal osteoblastic stroma will be evaluated by microscopy (electron) and IHC to discern

localization of radium-223 impact and pharmacodynamics. The purpose of this exercise is to characterize the levels of this decay product directly in the metastatic tumor in patients receiving radium-223.

Additionally, CTC samples will be collected at baseline (prior to initiating a new systemic therapy), month 3, and at progression (PSA or radiographic, determined by treating investigator). CTCs will be analyzed by a research CTC assay. Cells will be stained for DAPI, CD45, and pan-CK as per standard practice (Armstrong et al, 2011). A Profile Kit (Veridex) will also be used to enrich cells based on EpCAM expression and to exclude contaminating leukocytes. Profile kit captured CTC will also be stained for ALP, cytokeratin, PSA, PSMA expression by using standard commercial antibodies validated in control cells. Leukocytes from patients will be used as internal negative controls. AR amplification in CTC will be examined by FISH. Three additional research tubes for CTC analysis and ALP expression will be collected for flow cytometric analysis. Cells >4 microns will be analyzed for lack of CD45 and positive for ALP using Alexafluor immunofluorescence. This second experiment may identify more mesenchymal CTCs that have lost EpCAM expression and may be missed by the research Cellsearch assay. To examine whether any ALP-expressing CD45-negative cells are prostate cancer cells and not circulating stromal cells (i.e. osteoblasts), cells will also be evaluated for cytokeratin, PSA, and PSMA expression. Evaluable cells will also be examined for AR amplification by FISH to determine if AR amplifications present. These results will help determine if circulating prostate cancer cells undergo osteomimicry and express ALP and thus may be a potential biomarker for radium-223 efficacy in men with bone metastases. CTC sorted from two research tubes will be used for DNA and RNA isolation to proceed with aCGH, WES and RNA sequencing. Given that men with widespread bone metastases have variable levels of ALP and response to radium-223, identifying men most likely to benefit in this pre-docetaxel CRPC treatment space is clinically important.

In addition to CTCs, ALP levels will be evaluated prospectively over time along with PSA and CTC enumeration. Baseline clinical, pathologic, and demographic characteristics will be collected and treatment response and progression tracked over time. Bone scans and/or F-18 PET/CTs will be used to image patients as per standard of care practice.

Additional peripheral blood (15 ml) will be obtained prior to initiation of radium-223 for plasma isolation and whole blood RNA extraction. Extracted whole blood RNA will then be processed for RNA sequencing. And extracted peripheral blood plasma will be used for DNA isolation and target sequencing. CTC culture will also be attempted, based on flow cytometry sorting of cells with EpCAM+/CD45- or O-cadherin+/CD45- expression. The sorted EpCAM+/CD45- or O-cadherin+/CD45- CTCs will be conditionally cultured with multiple growth factors, Y-27632 and feeder cells to stimulate the CTC growth.

In order to evaluate the biomarkers of interest (bone protein biomarkers, DNA studies, RNA studies, and CTCs), we will utilize both cell line experiments as controls (negative and positive) and prospective patient samples (CTCs, metastases). Control cell lines (osteoblasts, C4-2, LnCAP, PC-3 cells) will be used to establish immunofluorescent (IF) parameters for scoring CTCs and used in real time, and archival tumor tissue controls will be used for immunohistochemistry (IHC) studies. Peripheral blood mononuclear cells (CD45+ cells) from patients will be used as real time internal controls for ALP expression in CTCs.

6.2 Selection of Dose and Treatment Duration

The dosage of Radium-223 dichloride is 50 kBq/kg body weights. The total activity to be injected will be calculated volumetrically using the patient's body weight (kg), the 50 kBq/kg (55 kBq/kg upon implementation of the NIST update) body weight dosage level, and the decay correction factor (DK) to correct for physical decay of Radium-223 dichloride. A table with DK values according to physical decay of the study medication will be provided with each vial of Radium-223 dichloride. The total amount (volume to be drawn into the syringe) to be administered to a patient should be calculated as follows:

Body weight (kg) x50 kBq/kg = volume to be injected (mL)
DK x 1000 kBq/mL

After NIST update:
Body weight (kg) x55 kBq/kg = volume to be injected (mL)
DK x 1100 kBq/mL

Duration of treatment with radium-223 is every 4 weeks up to 6 doses or until disease progression once eligibility has been verified and patient registered. Concurrent abiraterone acetate or enzalutamide and ongoing ADT and bone anti-resorptive agents is permitted.

6.3 Subject eligibility

In this study, 20 men with bone metastatic CRPC and progression will be prospectively consented and enrolled from the Duke Cancer Institute prostate cancer clinic.

Patients must have baseline evaluations performed prior to the first dose of study drug and must meet all inclusion and exclusion criteria. Results of the baseline evaluations, which assure that all inclusion and exclusion criteria have been satisfied, must be reviewed by the Principal Investigator or his/her designee prior to enrollment of that patient. In addition, the patient must be thoroughly informed about all aspects of the study, including the study visit schedule and required evaluations and all regulatory requirements for informed consent. The written informed consent must be obtained from the patient prior to any screening procedures being performed. The following criteria apply to all patients enrolled onto the study unless otherwise specified.

6.3.1 Inclusion criteria

Inclusion criteria

1) Age ≥ 18 years.
2) Life expectancy of at least 12 weeks (3 months).
3) Subjects must be able to understand and be willing to sign the written informed consent form. A signed informed consent form must be appropriately obtained prior to the conduct of any trial-specific procedure.
4) Histologically confirmed diagnosis of adenocarcinoma of the prostate. Histologic variants of prostate cancer, including neuroendocrine features are permitted; however, pure small cell carcinoma of the prostate is excluded.
5) Presence of >2 sites of metastatic disease in bone as determined by bone scan or CT, and for men who opt-in for bone biopsy, they must have at least one site amenable to radiographically-guided metastatic biopsy as determined by the study radiologist.
6) Symptomatic castration-resistant bone metastatic disease as determined by the provider.
7) Prior or concurrent therapy with either abiraterone acetate or enzalutamide.
8) Ongoing ADT using an LHRH agonist (e.g. leuprolide, goserelin) or antagonist (e.g. degarelix) must continue on therapy unless prior bilateral orchiectomy has been performed.
9) Current evidence of disease progression as evidenced by one of the following:
    a. 2 consecutive rising PSA levels separated at least 1 week apart above nadir PSA on last systemic therapy. If no nadir, then 2 rising PSA values greater than baseline pretreatment value is required from the most immediate prior therapy, **OR**
    b. CT or bone scan based evidence of disease progression with bone metastasis (new lesions or growth of existing lesions), **OR**

c. Evidence of symptomatic progression (increased pain in an area with known lesions confirmed on imaging).

10) All acute toxic effects of any prior treatment have resolved to NCI-CTCAE v4.0 Grade 2 or less.

11) Men of childbearing potential must agree to use adequate contraception beginning at the signing of the ICF until at least 30 days after the last dose of study drug. The definition of adequate contraception will be based on the judgment of the principal investigator or a designated associate.

12) Acceptable hematology and serum biochemistry screening values:

    a. White Blood Cell Count (WBC) ≥ 3,000/mm3
    b. Absolute Neutrophil Count (ANC) ≥ 1,500/mm3
    c. Platelet (PLT) count ≥ 100,000/mm3
    d. Hemoglobin (HGB) ≥ 9.0 g/dl
    e. Total bilirubin level ≤ 1.5 x institutional upper limit of normal (ULN)
    f. Aspartate aminotransferase (AST) and alanine aminotransferase (ALT) ≤ 2.5 x ULN
    g. Creatinine ≤ 1.5 x ULN
    h. Albumin > 2.5 g/dL

13) Willing and able to comply with the protocol, including follow-up visits and examinations

6.3.2 Exclusion Criteria

1) Treatment with cytotoxic chemotherapy within previous 4 weeks, or failure to recover from AEs down to grade 2 or less due to cytotoxic chemotherapy administered more than 4 weeks previous (however, ongoing neuropathy is permitted)

2) Receiving concurrent systemic therapy with radionuclides (e.g., strontium-89, samarium-153, rhenium-186, or rhenium-188) for the treatment of bony metastases. Prior therapy with radium-223 is not permitted.

3) Other malignancy treated within the last 3 years (except non-melanoma skin cancer or low-grade superficial bladder cancer)

4) Visceral (i.e. liver, lung, etc) metastases (pulmonary nodules ≤1cm are permitted) as assessed by chest, abdominal or pelvic computed tomography (CT) (or other imaging modality)

5) Presence of active untreated CNS parenchymal or epidural spinal metastases

6) Lymphadenopathy exceeding 3 cm in short-axis diameter

7) Imminent spinal cord compression based on clinical findings and/or magnetic resonance imaging (MRI). Treatment should be completed for spinal cord compression.

8) Any other serious illness or medical condition, such as but not limited to:

    a. Any infection ≥ National Cancer Institute Common Terminology Criteria for Adverse Events (NCI-CTCAE) version 4.0 Grade 2
    b. Cardiac failure New York Heart Association (NYHA) III or IV
    c. Crohn's disease or ulcerative colitis
    d. Bone marrow dysplasia
    e. Fecal incontinence

9) Inability to comply with the protocol and/or not willing or not available for follow-up assessments.

10) Any condition which, in the investigator's opinion, makes the subject unsuitable for trial participation.
11) Concurrent cytotoxic chemotherapy or anticancer therapies other than abiraterone, prednisone or other glucocorticoids, enzalutamide, androgen deprivation therapy, bisphosphonates, and denosumab.
12) Concurrent use of another investigational drug or device therapy (i.e., outside of study treatment) during, or within 2 weeks of treatment initiation.
13) Major surgery within 30 days prior to start of study drug.

6.4 Patient Registration

After signing informed consent and completing eligibility screening, subjects who are selected to participate will be registered. A record of subjects who fail to meet entry criteria (i.e., screen failures) will be maintained. Subject registration must be complete before beginning any treatment.

6.4.1 Informed Consent

Authorized study personnel should fully explain the scope of the study to each subject before obtaining informed consent. Subjects should be advised of any known risks inherent in the planned procedures, of any alternative treatment options, of their right to withdraw from the study at any time for any reason, and of their right to privacy.

When obtaining informed consent, study personnel should:
First: Confirm that the subject is a potential candidate for study participation.
Next: Obtain dated and signed informed consent.
Finally: Confirm that the subject is eligible as defined in **Section 6.3** (Inclusion/Exclusion Criteria). A record of subjects who fail to meet entry criteria (i.e., screening failures) will be maintained.

Patients will be enrolled only after all pre-treatment screening evaluations are completed and all eligibility criteria are met. Once the patient has signed consent and been found to meet all eligibility criteria, the subject will be enrolled, and a unique patient study identification number will be assigned.

6.5 Early Study Termination

This study can be terminated at any time for any reason by the PI-sponsor. If this occurs, all subjects on study should be notified as soon as possible. Additional procedures and/or follow up should occur in accordance with Section 10.7, which describes procedures and process for prematurely withdrawn patients. For more details about withdrawal from study and drop out, please refer to section 8.5.

7 STUDY DRUG

7.1 Names, Classification, and Mechanism of Action

Radium-223 dichloride, an alpha particle-emitting pharmaceutical, is a radiotherapeutic drug (Miller, 1965). Radium-223 dichloride is supplied as a clear, colorless, isotonic, and sterile solution to be administered intravenously with pH between 6 and 8. Each milliliter of solution contains 1,000 kBq (1,100 kBq/ml after implementation of the NIST update) radium-223 dichloride [27 microcurie (30 microcurie after NIST update)], corresponding to 0.53 ng radium 223, at the reference date. Radium is present in the solution as a free divalent cation. Each vial contains 6 mL of solution (6,000 kBq (162 microcurie) radium-223 dichloride at the reference date (6,600 kBq (178 microcurie) after implementation of the NIST update). The inactive ingredients are 6.3 mg/mL sodium chloride USP (tonicity agent), 7.2 mg/mL sodium citrate USP (for pH adjustment), 0.2 mg/mL hydrochloric acid USP (for pH adjustment), and water for injection USP.

The molecular weight of radium-223 dichloride, 223RaCl2, is 293.9 g/mol. Radium-223 has a half-life of 11.4 days. The specific activity of radium-223 is 1.9 MBq (51.4 microcurie)/ng. The six-stage-decay of radium-223 to stable lead-207 occurs via short-lived daughters, and is accompanied predominantly by alpha emissions. There are also

beta and gamma emissions with different energies and emission probabilities. The fraction of energy emitted from radium-223 and its daughters as alpha-particles is 95.3% (energy range of 5 - 7.5 MeV). The fraction emitted as beta-particles is 3.6% (average energies are 0.445 MeV and 0.492 MeV), and the fraction emitted as gamma-radiation is 1.1% (energy range of 0.01 - 1.27 MeV).

The active moiety of Radium-223 dichloride is the alpha particle-emitting isotope radium-223 (as radium Ra 223 dichloride), which mimics calcium and forms complexes with the bone mineral hydroxyapatite at areas of increased bone turnover, such as bone metastases (Cheetham, 2012; Henriksen, 2002; Henriksen, 2003). The high linear energy transfer of alpha emitters (80 keV/micrometer) leads to a high frequency of double-strand DNA breaks in adjacent cells, resulting in an anti-tumor effect on bone metastases. The alpha particle range from radium-223 dichloride is less than 100 micrometers (less than 10 cell diameters) which limits damage to the surrounding normal tissue.

7.2 Labeling

Radium-223 dichloride is manufactured by Algeta's contract manufacturer: Institute for Energy Technology, Isotope laboratories, Kjeller, Norway. The product is produced according to Good Manufacturing Practice (GMP). Labeling will be per standard of care practices per FDA guidelines.

7.3 General warning

Radium 223 dichloride should be received, used and administered only by authorized persons in designated clinical settings. The receipt, storage, use, transfer and disposal Radium-223 dichloride are subject to the regulations and/or appropriate licenses of the competent official organization. Radium-223 dichloride should be handled by the user in a manner which satisfies both radiation safety and pharmaceutical quality requirements. Appropriate aseptic precautions should be taken.

7.4 Radiation protection

The administration of Radium-223 dichloride is associated with potential risks for other persons (e.g. medical staff, care givers and members of the patient's family) from radiation or contamination from spills of body fluids such as urine, feces, or vomit. Therefore, radiation protection precautions must be taken in accordance with national and local regulations.

<u>For drug handling</u>

Follow the normal working procedures for the handling of radiopharmaceuticals and use universal precautions for handling and administration such as gloves and barrier gowns when handling blood and bodily fluids to avoid contamination. In case of contact with skin or eyes, the affected area should be flushed immediately with water. In the event of spillage of Radium-223 dichloride, the local radiation safety officer should be contacted immediately to initiate the necessary measurements and required procedures to decontaminate the area. A complexing agent such as 0.01 M ethylene-diaminetetraacetic acid (EDTA) solution is recommended to remove contamination.

<u>For patient care</u>

Whenever possible, patients should use a toilet and the toilet should be flushed several times after each use. When handling bodily fluids, simply wearing gloves and hand washing will protect caregivers. Clothing soiled with Radium-223 dichloride or patient fecal matter or urine should be washed promptly and separately from other clothing.

Radium-223 is primarily an alpha emitter, with a 95.3% fraction of energy emitted as alpha-particles. The fraction emitted as beta-particles is 3.6%, and the fraction emitted as gamma-radiation is 1.1%. The external radiation exposure associated with handling of patient doses is considerably lower in comparison to other radiopharmaceuticals for therapeutic purposes as the administered radioactivity will usually be below 8 MBq (0.216 mCi) (8.8 MBq (0.238 mCi) after NIST). In keeping with the **A**s **L**ow **A**s **R**easonably **A**chievable (ALARA) principle for minimization of radiation exposure, it is recommended to minimize the time spent in radiation areas, to maximize the distance to radiation sources, and to use adequate shielding. Any unused product or materials

used in connection with the preparation or administration are to be treated as radioactive waste and should be disposed of in accordance with local regulations. The gamma radiation associated with the decay of radium-223 and its daughters allows for the radioactivity measurement of Radium-223 dichloride and the detection of contamination with standard instruments.

7.5 Dose calibration

Radium-223 dichloride can be measured in a normal dose calibrator instrument. When written approvals for the use of Radium Ra 223 dichloride from the Radiation Protection Agency for the specific center have been received by the sponsor, a vial of Radium Ra 223 dichloride for technical use will be sent to the study center. (a new reference vial will be sent to each center corresponding to the updated NIST reference material). The dose calibration will be performed by the Nuclear Medicine Division at Duke, who is responsible for the administration of radium-223 to patients.

Different clinical study centers possess dose calibrators from various suppliers; thus, the isotope calibration factor may differ from center to center. Consequently, each center must perform the Radium-223 dichloride dial setting on their relevant dose calibrator(s) (upon notification by Bayer each center is required to update the dial settings to correspond to the new NIST standard). The current dial settings are to remain in effect until Bayer obtains FULL approval from the FDA for implementation. In preparation for implementation of the NEW dial setting, the clinical study center will receive a sealed vial labeled NIST standard containing a Radium Ra 223 dichloride solution for calibration only. The vial is identical to the vials used for study treatment. The amount of Radium-223 dichloride in the vial will be stated on the label. Instructions for the dial setting, including the calibration log form, will be enclosed with the dispatch of the calibration sample. All sites will be notified by Bayer when FINAL regulatory approval from the FDA is in place and the updated NISTstandardization is to be implemented.

7.6 Dosimetry

The absorbed radiation dose calculation was performed based on clinical biodistribution data. Calculations of absorbed doses were performed using OLINDA/EXM (Organ Level INternal Dose Assessment/EXponential Modeling), a software based on the Medical Internal Radiation Dose (MIRD) algorithm, which is widely used for established beta and gamma emitting radionuclides. For radium-223, which is primarily an alpha emitter, additional assumptions were made for the intestine, red marrow and bone/osteogenic cells to provide the best possible absorbed dose calculations for Radium-223 dichloride, considering its observed biodistribution and specific characteristics.

For an administered activity of 3.65 MBq (0.0987 mCi) (50 kBq (0.00135 mCi) (55kBq (.0015 mCi after NIST update)per kg body weight to a 73-kg adult), the calculated absorbed doses to the bone (osteogenic cells) is 4.2050 Gy (420.5 rad) and to the red marrow is 0.5066 Gy (50.66 rad). The calculated absorbed doses to the main excretory organs are 0.0265 Gy (2.65 rad) for the small intestine wall, 0.1180 Gy (11.8 rad) for the upper large intestine wall and 0.1696 Gy (16.96 rad) for the lower large intestine wall.

The calculated absorbed doses to other organs are low, e.g. heart wall (0.0063 Gy, 0.63 rad), lung (0.0003 Gy, 0.03 rad), liver (0.0109 Gy, 1.09 rad), kidneys (0.0117 Gy, 1.17 rad), urinary bladder wall (0.0147 Gy, 1.47 rad), testes (0.0003 Gy, 0.03 rad), and spleen (0.0003 Gy, 0.03 rad).

The hematological adverse drug reactions observed in the clinical studies with Radium-223 are much lower in frequency and severity than what could be expected from the calculated absorbed doses to the red marrow. This may be related to spatial distribution of alpha particle radiation resulting in non-uniform radiation dose to the red marrow.

7.7 Dose handling

The Radium-223 dichloride vials must be stored inside their lead container in a secure facility. The study drug should be used within 28 days of production. Radium-223 dichloride is an alpha-pharmaceutical and should be handled by individuals who are qualified by training and experience in the safe handling of radionuclides. One dedicated person and a back-up designee will have responsibility as assigned from the Primary Investigator for

handling and storage of Radium-223 dichloride. All administrations of Radium-223 dichloride are based on the certified activity of Radium-223 dichloride at the calibration date.

7.8 Dose calculation

The dosage of Radium-223 dichloride is 50 kBq/kg body weight (55kBq/kg after NIST update). The total activity to be injected will be calculated volumetrically using the patient's body weight on (kg), the 50 kBq/kg body weight dosage level (55kBq/kg after NIST update), and the decay correction factor (DK) to correct for physical decay of Radium-223 dichloride. A table with DK values according to physical decay of the study medication will be provided with each vial of Radium-223 dichloride. Radium-223 is an alpha particle emitter with a physical t1/2 of 11.4 days. The radioactive concentration at the reference date is 1,000 kBq/mL (1,100 kBq/mL after implementation of NIST update). The total amount (volume to be drawn into the syringe) to be administered to a patient should be calculated as follows:

Body weight (kg) x50 kBq/kg = volume to be injected (mL)
DK x 1000 kBq/mL

After the NIST update:
Body weight (kg) x55 kBq/kg = volume to be injected (mL)
DK x 1100 kBq/mL

Data regarding activity should be recorded on the appropriate electronic case report form (eCRF) page.

7.9 Dose preparation

Personnel should use appropriate protective clothing and equipment during syringe filling and application to prevent contamination with the radioactive solution (medical gloves / protective glasses). The individual responsible for study drug preparation will draw the correct volume of study drug into a syringe. The size of the syringe should be chosen according to the applied volume to reach the required dosing accuracy. Radium-223 dichloride should not be diluted or mixed with any solutions. Do not store above 40°C (104°F). If the vials have been stored in a refrigerator, they should be left at room temperature for 1 hour prior to use, since cold material should not be injected in a patient. Store Radium-223 dichloride in the original container or equivalent radiation shielding. This preparation is approved for use by persons under license by the Nuclear Regulatory Commission or the relevant regulatory authority of an Agreement State.

7.10 Dose administration

Before administration of study drug, the patient must be well hydrated; the patient should be instructed to drink ad libitum. Aseptic technique should be used in the administration of Radium-223 dichloride. The syringe should be handed over to the individual who will perform the injection. The study medication will be administered as a bolus intravenous (IV) injection (up to 1 minute) at intervals of every 4 weeks for up to 6 cycles or till progression, whichever happens first. After administration, the equipment used in connection with the preparation and administration of drug is to be treated as radioactive waste and should be disposed in accordance with local procedure for the handling of radioactive material.

7.11 Dose Modification

Every effort should be made to administer the full dosing regimen of Radium-223 dichloride. Adjustment of dose level is not permitted.

Study visits during the treatment period should occur at 4 weeks intervals (within a window of +/- 7 days). Circumstances under which dosing delays may be instituted include but are not limited to the following:

Disease progression: The Investigator should delay cytotoxic chemotherapy, other systemic radioisotope, hemibody external radiotherapy or other investigational drug until the follow-up period. If such treatments have

to be given during the treatment period, further study drug administrations must be discontinued. Patients with disease progression may continue treatment at the Investigator's discretion. Myelosuppression:

Changes in hematology parameters:

- If a patient experiences CTCAE v4.0 Grade 3 or 4 neutropenia, thrombocytopenia, or anemia the administration of study drug should be delayed until recovery to Grade 2 or better.
- If a patient experiences CTCAE v4.0 Grade 3 or 4 neutropenia, thrombocytopenia, or anemia lasting > 14 days, further study drug administrations must be discontinued.
- Blood transfusion is acceptable between study drug administrations. Use of biologic response modifiers, such as G-CSF or GM-CSF, is allowed in the management of acute toxicity.

Gastrointestinal events: Diarrhea should be treated as per local practice. A further dose of study medication should not be given before diarrhea is recovered to CTCAE v4.0 Grade 2 or baseline levels. Nausea or vomiting should be treated as per local practice. A further dose of study medication should not be given before nausea or vomiting is recovered to CTCAE v.4.0 Grade 2 or baseline levels.

Spinal Cord Compression: If the patient experiences spinal cord compression during the treatment period, the patient should be treated for the event, and may receive further study drug administration if adequately recovered.

Surgical Intervention: If surgery is required, the patient should continue with study treatment, if this is considered safe in the treating Investigator's opinion. The surgeon needs to be notified that the patient has been given radioactive drug, and needs to follow the guidelines for radioactive protection.

Non-pathological fractures: For traumatic fractures in weight-bearing bones during treatment phase, the study drug administration should be delayed for 2-4 weeks from the time of fracture.

Pathological fractures: Pathological fractures may occur as the result of either progressive disease or increased physical activity associated with significant pain palliation. Pathologic fractures are to be treated in a manner that attempts to maintain the best functional status and quality of life. Study treatment may continue as planned.

7.12 Compliance and Accountability

An adequate record of receipt, distribution, and destruction of all study drugs must be kept in the form of a Drug Accountability Form.

Subject compliance with the treatment and protocol includes willingness to comply with all aspects of the protocol, and to have blood collected for all safety evaluations. At the discretion of the principal investigator, a subject may be discontinued from the trial for non-compliance with follow-up visits or study drug.

The investigator, or a responsible party designated by the investigator, must maintain a careful record of the inventory and disposition of the agent (investigational or free of charge) using the NCI Drug Accountability Record or another comparable drug accountability form. (See the CTEP website at http://ctep.cancer.gov/protocolDevelopment for the "Policy and Guidelines for Accountability and Storage of Investigational Agents" or to obtain a copy of the drug accountability form.)

7.13 Prior and Concomitant Therapy

All medication that is considered necessary for the subject's welfare, and which is not expected to interfere with the evaluation of the study treatment, may be given at the discretion of the investigator. All medications (including contrast media) taken within 2 weeks prior to the start of the study and during the study must be recorded in the subject's source documentation and in the CRF (including start/stop dates, dose frequency, route of administration, and indication).

Permitted

- Treatment with non-conventional therapies (e.g., herbs [with the exception of St. John's Wart], acupuncture) and vitamin/mineral supplements is acceptable provided that, in the opinion of the investigator, such treatment will not interfere with the trial endpoints.
- Subjects may receive standard of care for any underlying illness.
- In the event of neutropenia, anemia, or thrombocytopenia, subjects may receive appropriate supportive care (e.g., transfusion, biologic response modifiers such as G-CSF or GM-CSF, prophylactic antibiotics, antifungals and/or antivirals, hematopoietic growth factors). This supportive care should not substitute a recommended dose modification.
- Blood transfusions and erythropoietin are allowed during the study period.
- If surgery is required during study drug treatment, the surgeon needs to be notified that the patient has been treated with a radioactive product and adequate precautions for radioactive protection should be applied during the surgical procedure. The patient should continue with study treatment if considered safe in the treating Investigator's opinion.
- Concomitant treatments for prostate cancer will be recorded in the CRFs. These treatments may include, but are not necessarily limited to: Luteinizing-Hormone-Releasing hormone (LHRH) analogs or antagonists, surgery, palliative external beam radiation therapy, enzalutamide, or abiraterone, bicalutamide, flutamide, nilutamide, ketoconazole, and corticosteroids. Combined use of enzalutamide or abiraterone is not permitted. Additional anti-cancer agents are not permitted with radium-223 including experimental agents. **Treatment with new anti-cancer agents should not be started during the screening period or during the first 3 months of therapy with radium-223 but treatments ongoing at the time of screening and progression at entry may be continued during radium-223 therapy.** Dutasteride or finasteride use are permitted.

7.14 Disposal and Destruction

At the end of the study, unused supplies of Radium-223 dichloride should be destroyed according to institutional policies. Destruction will be documented in the Drug Accountability Record Form. The certificate of destruction should be sent to Bayer as per standard of care practice.

8 SCREENING AND ON-STUDY TESTS AND PROCEDURES

The following table summarizes tests and procedures that occur during the screening phase, treatment phase, maintenance phase, and follow-up phase of the protocol.

Procedures	Screening Baseline[a]	Study Visits			Follow up
	Within 30 days of registration	Day 1 of each cycle (+/- 7 days)	Cycle 3 Day 15 (+5 days)	Cycle 6 Day 15 (+10days) or at progression (clinically defined, + 30 days)	28 days post last dose of radium (+/- 7 days)
Informed consent	X				
Inclusion / exclusion criteria	X				
Demographic data	X				
Prior and concomitant medications	X	X	X	X	X
Medical history, pain and AE assessment[b]	X	X	X	X	X
Vital signs, height, and weight[c]	X	X	X	X	X
Karnofsky performance status[d]	X	X	X	X	X
Physical examination[b]	X	X	X	X	X
Tumor Site Assessments: CT scans (chest, abdomen, pelvis) and bone scan[e]	X			X	
Radium-223 therapy		X[f]			
Standard-of-care laboratory assessments[g]					
CBC with differential [g]	X	X	X	X	X
Serum chemistries[g]	X	X	X	X	X
PT/PTT[k]		X[k]	X	X	
Testosterone, total	X				
PSA, total[g]	X	X		X[l]	
LDH[g]		X		X[l]	
Correlative studies for research					
Metastatic bone biopsy[h]		X	X	X	
Bone alkalkine phosphatase[m]		X		X[l]	
CTC research samples[i]		X		X[l]	
Peripheral blood plasma cfDNA and peripheral whole blood RNA extraction[j]		X			

<u>Footnotes to Study Flow Chart</u>:

a. Screening/baseline evaluations must be completed within 30 days prior to registration.

b. Medical history, physical examination, pain assessments and AE assessments will be performed at each time point.

c. Vital signs and weight will be recorded at each visit.

d. Karnofsky performance status (Appendix 1) will be evaluated and recorded at each time point.

e. CT with IV contrast of the chest, abdomen, and pelvis and Tc-99 bone scans will be performed within 30 days prior to registration (screening), and at progression or after cycle 6 as part of routine tumor assessments. Imaging may be performed locally or at Duke University. NaF (F-18) PET/CT imaging may be used in eligible men who enroll on the Medicare NOPR registry in place of the CT and bone scan.

f. **Radium-223** will be given every 4 weeks (with a 2 week window) for up to 6 doses until progression. Dosing will take place in Duke nuclear medicine as part standard institutional practice under the guidance of Dr. Bennett Chin.

g. **Standard-of-care laboratory assessments** to be collected at the following intervals (+/- 14 days) and will include:

   <u>Complete blood count (CBC) with differential</u>: WBC count with differential, platelet count, hemoglobin, and hematocrit. To be performed at baseline and up to 14 days prior to each cycle of radium-223, which can be done at either Duke or patient's local hospital or laboratory. When collected within 14 days prior to the treatment, this does not need to be repeated on the day of the visit. This is also to be collected prior to any bone biopsy procedure, at progression and at the 28 days post last dose of study drug visit.

   <u>Serum chemistries:</u> Sodium, potassium, chloride, blood urea nitrogen (BUN), creatinine, glucose, carbon dioxide or bicarbonate, calcium, phosphorus (optional), total protein, albumin, aspartate aminotransferase (AST), alanine aminotransferase (ALT), total bilirubin, and alkaline phosphatase. To be performed at baseline and up to 14 days prior to each cycle of radium-223 dosing, which can be done at either Duke or patient's local hospital. When collected within 14 days prior to the treatment, this does not need to be repeated on the day of the visit. This is also to be collected prior to any bone biopsy procedure, at progression and at the 28 days post last dose of study drug visit.

   <u>LDH</u>: Lactate dehydrogenase. Drawn within 14 days of cycle 1 day 1, cycle 3 day 1 and cycle 6 day 1 (or at progression).

   <u>PSA</u>: Drawn within 14 days of baseline, cycle 3 day 1, and at progression.

   <u>Testosterone</u>: Drawn at screening only.

h. **Optional metastatic tumor biopsies (bone)**: performed under radiologic guidance is to be performed prior to radium-223 initiation, following informed consent and determination of eligibility and registration. The first biopsy may be up to 7 days prior to radium-223 initiation. The second biopsy will be at 15-20 days after cycle 3 radium-223 treatment. The third biopsy will be 15-25 days after the cycle 6 radium-223 given or on progression, and this is optional. Up to 3 FFPE and 2 frozen (OCT) at each time point will be collected (5 total), and for the cycle 3 and 6 time points, 1 fresh (6 total) samples will be collected at each time for correlative studies (see laboratory manual). IHC staining will be done on FFPE sample for expression of PSA, CK, ALP, O-cadherin, N-cadherin, vimentin, TWIST, SNAIL, beta-catenin, ZEB1 and γ-H2AX; and FISH will be done for androgen receptor (AR) expression and AR variants. Fresh samples will also be utilized for autoradiography of radium-223 and its decay products. OCT sample will be used for DNA and RNA isolation to proceed to aCGH (array comparative genomic hybridization) and RNA sequencing. The bone metastasis biopsies on treatment/or progression will be compared with baseline biopsy for changes in apoptosis (TUNEL, cleaved caspase-3 expression), reduction in proliferation (Ki-67), the abundance of tumor cells (CK + or PSA + cells in bone). Bone metastasis will be sampled at the edge of metastatic lesion and adjacent to the lesion to survey the extent of physiologic impact of radium-223 on apoptosis and proliferation regionally where feasible. Bone metastatic tumor tissue will also be used to evaluate the final delay product of Ra-223 using assays by a member of our team in nuclear medicine. **The third bone biopsy is also optional. Standard-of-care biopsies with extra cores taken for research are also allowed. The patient and the investigator will determine whether the biopsy will be considered standard-of-care or research only.**

i. **Circulating tumor cells for correlative studies:** 7.5 mL Cellsave tubes will be collected (2) on day 1 of cycles 1, cycle 3, and at cycle 6 day 1 or at progression. In addition, 10 mL EDTA tubes (5) will be collected at the same time points for flow cytometric analysis. A total of 90 mL for research is planned maximally over an 8 week

period. Leftover samples may also be used for cfDNA analysis. CTCs will be examined for enumeration (research Cellsearch assay), ALP, CK, CD45, DAPI expression by IHC.

j. Additional peripheral blood will be obtained prior to initiation of radium-223 for plasma cfDNA and whole blood RNA extraction for genomic profiling at cycle 1 day 1 (see laboratory manual).

k. PT/PTT is to be performed prior to the bone biopsy visits only (per standard of care), not at all Day 1 visits.

l. Drawn at progression only.

m. **Bone Alkaline Phosphatase:** Bone alkaline phosphatase will be drawn at cycle 1 day 1, cycle 3 day 1, cycle 6 day 1 and at progression.

8.1 Screening Examination

The screening examination will take place within 30 days of registration. An informed consent form must be signed by the patient before any screening procedure takes place. Subject data to be collected at the Screening Examination includes:

- demographic data
- prior and concomitant medications
- medical history and AE assessment
- vital signs, height, and weight
- karnofsky performance status
- physical examination
- pain assessment
- tumor Site Assessments: CT scans (chest, abdomen, pelvis) and bone scan
- CBC with differential
- serum chemistries including testosterone, PSA, and CTCs (if available). Patient registration must be complete before beginning any treatment or study activities.

8.2 Treatment Period

After registration, radium-223 will be given at dose of 50 kBq/kg every 4weeks for up to 6 doses (55kBq/kg body weight after implementation of the NIST update). Subject data to be collected during the Treatment Period includes:

<u>Day 1 of each cycle (+/- 7 days):</u>

- Medical history and AE assessment
- Prior and concomitant medications
- vital signs and weight
- karnofsky performance status
- physical examination
- pain assessment
- Radium-223 therapy (CBC-diff and CMP must be obtained within 14 days prior to treatment, either at Duke or locally)
- CBC with differential (does not need to be repeated if obtained within 14 days prior to treatment)
- Other SOC lab assessments within 14 days of treatment: serum chemistries, PSA (Cycle 3 only and at progression), LDH (cycle 1, 3, and 6 only or at progression)
- correlative studies cycle 1 day 1, cycle 3 day 1, and cycle 6 day 1: bone alkaline phosphatase, CTC research samples, blood for DNA and RNA extraction.
- First optional bone biopsy is to be performed up to 7 days prior to the first radium-223 dose. Subsequent biopsies will be performed as detailed below. A CBC with differential and PT/PTT (per standard of care) must be obtained prior to any biopsy.

<u>Cycle 3 Day 15 (+5 days) - *Optional*:</u>

- Second optional bone biopsy.

- vital signs
- physical examination
- pain assessment
- CBC with differential
- PT/PTT (day of biopsy per standard of care))
- serum chemistries

8.3 End of Treatment

Radium-223 will be given at dose of 50 kBq/kg (55kBq/kg after implementation of the NIST update) every 4weeks till progression or up to 6 doses, whichever happens first. Patient will be followed in the Duke Cancer Institute GU oncology clinic while on study, and then 28 days (+/- 7days) after patients finish the last dose of radium-223 or post progression.

Subject data to be collected at the end of treatment visit (either Cycle 6 Day 15 visit or at progression) include:

- medical history and AE assessment,
- prior and concomitant medications
- vital signs and weight,
- karnofsky performance status,
- physical examination,
- pain assessment,
- tumor Site Assessments: CT scans (chest, abdomen, pelvis) and bone scan,
- CBC with differential,
- serum chemistries
- Optional bone biopsy (If biopsy is to be performed, a PT/PTT should also be collected per standard of care)
- Labs to be drawn at progression only (not cycle 6 day 15): PSA, LDH, CTC (standard of care and research) and Bone Alkaline Phosphatase.

Subject data to be collected 28 days after patient finished the last dose of radium-223:

- medical history and AE assessment,
- vital signs and weight,
- karnofsky performance status,
- physical examination,
- CBC with differential,
- serum chemistries.

Following progression, ascertainment of the date of death and cause of death will be performed every 3 months (+/- 14 days) through chart review, patient contact, or death index reviews.

8.4 Interruption or discontinuation of treatment

For patients who are unable to tolerate the protocol-specified dosing schedule, dosing delays according to 7.11 are permitted in order to keep the patient on study drug. If administration of radium 223 must be interrupted because of unacceptable toxicity, drug dosing will be interrupted or modified according to rules described in 7.11. Ultimately, discontinuation of the study drug is at the discretion of the treating physician.

Toxicity will be assessed using the NIH-NCI Common Terminology Criteria for Adverse Events, version 4.0.

8.5 Early Withdrawal of Subject(s)

8.5.1 Criteria for Early Withdrawal, drop outs/screen failures

Subjects **must be withdrawn from the trial** (treatment and procedures) for the following reasons:

- Subject withdraws consent from study treatment and study procedures. A subject must be removed from the trial at his own request or at the request of his/her legally acceptable representative. At any time during the trial and without giving reasons, a subject may decline to participate further. The subject will not suffer any disadvantage as a result.
- Subject is lost to follow-up.
- Death.

Subjects **may be** withdrawn from the study for the following reasons:

- The subject is non-compliant with study drug, trial procedures, or both; including the use of anti-cancer therapy not prescribed by the study protocol.
- If, in the investigator's opinion, continuation of the trial would be harmful to the subject's well-being.
- The development of a second cancer.
- Development of an intercurrent illness or situation which would, in the judgment of the investigator, significantly affect assessments of clinical status and trial endpoints.
- Deterioration of KPS performance status to <40%.
- Use of illicit drugs or other substances that may, in the opinion of the investigator, have a reasonable chance of contributing to toxicity or otherwise skewing trial result.

Any subject removed from the trial will remain under medical supervision until discharge or transfer is medically acceptable. In all cases, the reason for withdrawal must be recorded in the CRF and in the subject's medical records.

A subject who discontinues study participation prematurely for any reason is defined as a “dropout” if the subject has already been administered at least one dose of study drug.

A subject who, for any reason (e.g. failure to satisfy the selection criteria), terminates the study before the time point used for the definition of “dropout” (see above) is regarded a “screening failure”.

8.5.2 Recording Requirements for Early Withdrawal

All interruptions or changes to study drug administration must be recorded. It will be documented whether or not each patient completed the clinical study. If for any patient either study treatment or observations were discontinued the reason will be recorded.

8.5.3 Replacement of Early Withdrawal(s)

No withdrawn subjects will be replaced.

8.6 Study Assessments

8.6.1 Medical History

Medical history findings (i.e. previous diagnoses, diseases or surgeries) meeting all criteria listed below will be collected:

- Not pertaining to the study indication
- Start before signing of the informed consent

- Considered relevant to the study.

8.6.2 Vital signs, Performance status and Physical Exam

Vital sign assessment consists of height (first visit), pulse, blood pressure, respiration rate, temperature and weight. Blood pressure, pulse and respiration rate should be measured on patients in the sitting position.

Performance status will be assessed at screening and per the visit schedule using the Karnofsky performance status scale (Appendix 1).

Physical examination will comprise a total body examination (general appearance, skin, neck, including thyroid, eyes, ears, nose, throat, lungs, heart, abdomen, back, lymph nodes, extremities and basic nervous system). Significant findings made after the start of study drug which meet the definition of an Adverse Event must be recorded. Following progression, ascertainment of the date of death and cause of death will be performed every 3 months (+/- 14 days) through chart review, patient contact, or death index reviews.

8.6.3 Laboratory Evaluations

The following laboratory studies will be obtained at specified intervals to assess subject safety and disease progression.

- **Hematology:** Complete blood count (CBC) consisting total white blood cell count (WBC) with differential (total neutrophil count including bands, lymphocyte, monocyte, eosinophil, and basophil counts), hemoglobin, hematocrit, and platelet count.
- **Blood chemistry:** Blood urea nitrogen (BUN), creatinine, sodium, potassium, chloride, calcium, phosphorus, glucose, albumin, total protein, total bilirubin (direct and indirect), ALP, AST (SGOT), ALT (SGPT).
- **Standard-of-care biomarkers**: include PSA, lactate dehydrogenase (LDH), and circulating tumor cell enumeration, to be drawn prior to initiation and after each cycle of therapy. If CTC is evaluated at screening, another evaluation will not be performed on cycle 1 day 1, if drawn within 14 days of cycle 1 day 1.Circulating tumor cells to be drawn when available.

8.6.4 Imaging

A total body Tc^{99} nuclear bone scan, F-18 PET/CTs or CT scan of chest, abdomen and pelvis will be performed within 30 days of registration and on progression. The imaging can be done at Duke Hospital or at patient's local hospital.

8.6.5 Exploratory correlative studies

Bone metastasis biopsy (optional): All patients who consent for this optional test will have a biopsy of bone metastasis prior to initiation of therapy. A radiologist must confirm that a site is amenable to biopsy as part of eligibility determination. At each biopsy, up to 6 core biopsies will be taken, including, as available, 3 cores from near the center of the lesion, 2 cores from the periphery approximately 1 cm outside of the lesion, and 1 additional core if available from either region. 1-2 cores will be collected on optimal cutting temperature (OCT) media and up to 3 cores will undergo immediate formalin fixation, and one core will be collected fresh (cycle 3 and cycle 6 biopsies only). For details of collection of tissue from, refer to the laboratory manual established for this study. Up to 3 cores from each site biopsy will be formalin preserved for IHC and FISH studies, 1-2 cores will be flash frozen on OCT for DNA and RNA extraction, and a single center fresh core will be used to evaluate the decay product of Ra-223 in bone. Frozen tissue will be stored in liquid nitrogen in the GU Oncology laboratory of Dr. Armstrong in the Snyderman building, 3rd floor. Formalin fixed samples will be stored in the Research Immunohistology Lab in the Duke Department of Pathology. The second and third optional bone biopsies will be obtained 15-20 days after cycle 3 radium-223 and 15-25 days after cycle 6 treatment/or progression to explore the regional extent of radium-223 efficacy as determined by the physiologic response in the bone metastasis. Cycle 3 Day 15 visit and assessmets are only required if the subject opts in for the biopsy. Standard-of-care biopsies with extra cores taken for research

are also allowed. The patient and the investigator will determine whether the biopsy will be considered standard-of-care or research only.

Processing of biopsied tissue: Biopsied bone metastasis samples will be processed and stored for analysis relating to genetic, genomic, and protein-based studies that may predict who will benefit from radium 223 therapy. The samples will be labeled with a subject number (such as 01-001, 01-002 etc) and subject initials. Samples will also be stored for protocol-planned and future specified prostate cancer research.

The formalin fixed tissue will be used to detect the co-expression of epithelial and prostate cancer biomarkers PSA, and CK by IHC staining with specific antibodies to PSA or CK. Some samples will also be examined for expression of ALP by IHC with specific antibody to ALP. Formalin fixed metastatic tissue will also be evaluated for expression of other biomarkers known to promote epithelial plasticity, including O-cadherin, N-cadherin, vimentin, TWIST, SNAIL, beta-catenin, ZEB1 and γ-H2AX by IHC. Androgen receptor (AR) and AR variant will be evaluated by FISH. FISH and IHC will be done by Dr. Wen Chi Foo in Pathology Department of Duke Hospital.

OCT frozen tissues will be used for DNA and RNA extraction to proceed with aCGH and RNA sequencing. Laser microdissection (LCM) will be explored to isolate tumor cells from OCT frozen bone biopsy samples to proceed to DNA and RNA extraction. aCGH genomic copy number profiling, whole exome sequencing (WES) and RNA expression profiling from RNA sequencing will be compared between patients who have high vs. low baseline ALP. Please refer to the laboratory manual for details of handling and processing instructions. aCGH profiling, WES and RNA sequencing data will be analyzed by Dr. David Corcoran in Omics Data Analysis Core of Duke University.

Fresh biopsied sample will be used to evaluate the decay product of Ra-223 in bone, which will be done by Dr. Zalutsky's Lab of our team in nuclear medicine. This will be processed using an automated dual-window sodium iodide gamma counter (Perkin Elmer Model 1480) located in Room 155, Bryan Research Building. A single energy window will be set to encompass emissions fromradium-223 and its decay products. Biopsy samples will be placed in sealed plastic tubes and counted in the gamma counter. Activity levels will be compared with ^{223}Ra samples of known activity (about 0.1 mCi), representing a known fraction of the injected dose administered to the patient, and will be corrected for room background radioactivity. Because it is anticipated that radioactivity levels in the biopsy samples will be low, each tube will be counted for 10 minutes or until 1000 net counts are reached, which corresponds to about a 3% counting error. The measurement will be repeated three times. Results will be expressed as nCi per gram tissue and percent injected dose per gram tissue. FFPE sample will be utilized for autoradiography of radium-223 and its decay products bond to hydroxyapatite in Ilford K5 emulsion, which will also be done by Dr. Zalustky's lab.

The second and third bone biopsies will be obtained 15-20 days after cycle 3 radium-223 treatment and 15-20 days after cycle 6 treatment/or on progression to explore the regional extent of radium-223 efficacy as determined by the physiologic response in the bone metastasis. This will be determined by changes in expression of apoptosis markers including TUNEL and cleaved caspase-3 expression by IHC staining, and reduction in proliferation (by Ki-67), the abundance of tumor cells (CK + or PSA + cells in bone) after cycle 1 of radium-223 and cycle 6/on progression as compared to baseline. We will sample the bone metastasis at the edge of metastatic lesion and 1cm adjacent to the lesion to survey the extent of physiologic impact of radium-223 and describe the changes in apoptosis (TUNEL, cleaved caspase-3 expression) and proliferation (Ki-67) regionally.

Research CTC Assays: Blood will be collected aseptically by venipuncture or from a venous port into 2 CellSave® Preservative Tubes and 5 EDTA tubes. The tubes will be filled until blood flow stops to ensure the correct ratio of sample to anticoagulant and preservative, for a total of approximately 7.5 mL of blood into the CellSave® tube and 10 ml of blood into the EDTA tube. It is estimated that total blood collection will not exceed 90 mL of blood per subject over 8 weeks period. The tubes will be immediately mixed by gently inverting eight times to prevent clotting. Blood

samples may be stored or transported in CellSave® Preservative Tubes for up to 96 hours and in EDTA tubes for up to 48 hours at room temperature prior to processing. All samples will be hand delivered to:

A Profile Kit (Veridex) will be used to enrich CTCs in 1 CellSave tube based on EpCAM expression and to exclude contaminating leukocytes; and enriched cells will be studied for ALP, CK, CD45, DAPI. The 2nd 7.5 mL CellSave tube will be used for leukocyte isolation. Isolated leukocytes from patients will be used as internal negative controls. 5 research 7.5 mL EDTA tubes will be collected for flow cytometric analysis for the following studies. First, cells >4 microns will be analyzed for positive ALP expression but lack of CD45 using immunofluorescence by flow. This experiment may identify more mesenchymal CTCs that have lost EpCAM expression and may be missed by the research CellSearch assay. To examine whether any ALP-expressing CD45-negative cells are prostate cancer cells and not circulating stromal cells (i.e. osteoblasts), 1 EDTA tube sorted cells will be stained for cytokeratin, PSA, and PSMA expression. Evaluable cells from profile kit and flow cytometry will also be examined for AR amplification by FISH to determine if AR amplification is present. Second, flow sorted cells from an EDTA tubes (2) will be used for DNA isolation to precede to aCGH and WES.. For more details about DNA isolation, aCGH, WES and RNA sequencing, please refer to the lab manual. 2 EDTA tubes will be used for CTC culture as the following.

CTC culture: Two 7.5 mL research EDTA tubes blood will be sorted for CTCs through flow cytometry based on expression of EpCAM+/CD45- or O-cadherin+/CD45- on CTCs. The sorted EpCAM+/CD45- or O-cadherin+/CD45- CTCs will be plated into media pre-coated 96 well plate respectively. In brief, the sorted EpCAM+/CD45- or O-cadherin+/CD45- CTCs will be plated into ultralow attachment plates (Corning) containing tumor sphere medium consisting of RPMI-1640 medium (with phenol red) supplemented with EGF (20ng/ml), basic FGF (20ng/ml), B27 (10ml), and 1x Antibiotic-antimycotic (Life Technologies). Culture media were change every 3-4 days under microscopic monitoring of cell clusters.
Please refer to lab manual for more details.

Circulating plasma DNA target sequencing: An additional tube of peripheral blood will be obtained prior to initiation of radium-223 cycle one for plasma DNA extraction. 10 ml blood will be collected directly into Cell-Free DNA BCT tube (Streck). Cell free DNA (cfDNA) will then be purified from the plasma. Isolated DNA will be preceded for targeted sequencing involving isolation of genomic regions of interest. cfDNA analyses may also be performed on leftover research blood samples collected at C3D1 and C6D1 or progression.

Peripheral whole blood RNA profiling: Peripheral whole blood RNA will be extracted prior to the initiation of radium-223 cycle 1. 2.5ml peripheral blood will be collected into PAXgene blood RNA tubes for RNA isolation and 2 tubes will be collected for duplication. Globin mRNA will be depleted from total RNA, and gene expression profiling will be performed using Illumina HiSeq.

Controls: Cultured cell lines including osteoblast cell line (HOb) and prostate cancer cell lines (C4-2, LnCAP, PC-3 cells) will be used to establish IHC and immunofluorescent (IF) parameters for ALP expression to help score CTCs and used in real time. Archival tumor tissue controls will be used for immunohistochemistry (IHC) studies. Peripheral blood mononuclear cells (CD45+ cells) from patients will be used as real time internal controls for ALP expression in CTCs.

Disease progression and survival: Response and progression will be evaluated in this study using a combination of the international criteria proposed by the Response Evaluation Criteria in Solid Tumors (RECIST) Committee (Eisenhauer, 2009, updated as RECIST 1.1) and the guidelines for prostate cancer bone scan endpoints developed by the Prostate Cancer Clinical Trials Working Group (PCWG2) (Scher, 2008).

PSA Because the rate of rise has known prognostic significance, estimate a pretreatment PSA doubling time (PSA-DT) if at least 3 values are available, but do not delay either treatment or enrollment onto a trial simply to estimate PSA-DT. To report PSA-based outcomes, PCWG2 recommends that the percent of change in PSA from baseline to 12 weeks (or earlier for those who discontinue therapy) and the maximum decline in PSA that occurs at any point after treatment be reported for each patient using a waterfall plot. Because declines in serum PSA, if they occur,

may not do so for several weeks, PSA measurements obtained during the first 12 weeks should not be used as the sole criterion for clinical decision making. As long as patient safety is the primary concern, in the absence of other indicators of disease progression, therapy should not be discontinued solely on the basis of a rise in PSA.

Target Lesions Bone metastasis lesions will be followed by bone scan and CT scan. Record post-treatment changes as either “no new lesions” or “new lesions.” Disease progression in bone is defined as 2 or more new lesions seen on bone scan compared with the baseline scan used for trial entry. In situations where scan findings suggest a flare reaction or where new lesion(s) may represent trauma, confirm these results with other imaging modalities. In the absence of clearly worsening soft-tissue (nodal and visceral) disease or disease-related symptoms, progression at the first scheduled assessment should be confirmed on a second scan performed 6 or more weeks later.

Symptoms Transient increases in pain may occur before improvement, and those occurring in the first 12 weeks should not affect the course of treatment in the absence of other compelling evidence of disease progression. Changes in symptoms should be documented and confirmed as per other outcome measures.

Overall response Patients with global deterioration of health status who require treatment to be discontinued without objective evidence of disease progression should be classified as having symptomatic deterioration. Every effort should be made to document their objective progression, even after discontinuing treatment.

Patients who do not have tumor response assessment due to rapid progression or toxicity will be considered nonresponders, will be included in the denominator for the response rate, and will be classified into one of the categories listed below:

- Death attributed to disease progression
- Early discontinuation attributed to disease progression
- Death attributed to drug toxicity
- Early discontinuation attributed to drug toxicity

Progression free survival Progression is defined as:

- Radiologic progression by RECIST 1.1 and/or bone scan progression by PCWG2 criteria, or
- Symptomatic progression, including clinical deterioration requiring new systemic therapy or a new a skeletal-related event (pathologic fracture, need for radiation to tumor site, malignant spinal cord compression).

A rise in PSA alone or CTC number alone, in the absence of radiologic or symptomatic indicators of disease progression, will not be considered disease progression. Increased pain alone, in the absence of changes in imaging or need for radiation therapy, will not be considered disease progression.

All assessments of disease should be collected at the same time interval. Post-treatment changes will be confirmed based on measurable target lesions, radionuclide bone scans, and symptoms as indicated in appendix 3.

Overall survival Patients will be followed for up to 36 months after study enrollment. Patients surviving past this time will be followed every three months, and their date of death will be recorded. Following progression, ascertainment of the date of death and cause of death will be performed every 3 months (+/- 14 days) through chart review, patient contact, or death index reviews.

9 SAFETY MONITORING AND REPORTING

The PI is responsible for the identification and documentation of adverse events and serious adverse events, as defined below. At each study visit, the PI or designee must assess, through non-suggestive inquiries of the subject or evaluation of study assessments, whether an AE or SAE has occurred.

9.1 Adverse Events

An adverse event (AE) is any untoward medical occurrence in a subject receiving study drug and which does not necessarily have a causal relationship with this treatment. For this protocol, the definition of AE also includes worsening of any pre-existing medical condition. An AE can therefore be any unfavorable and unintended or worsening sign (including an abnormal laboratory finding), symptom, or disease temporally associated with the use of radium 223, whether or not related to use of the radium 223. Abnormal laboratory findings without clinical significance (based on the PI's judgment) should not be recorded as AEs. But laboratory value changes that require therapy or adjustment in prior therapy are considered adverse events.

From the time the subject signs the informed consent form through the End of Study visit, all AEs must be recorded in the subject medical record and adverse events case report form.

AEs will be assessed according to the CTCAE version 4.0. If CTCAE grading does not exist for an AE, the severity of the AE will be graded as mild (1), moderate (2), severe (3), life-threatening (4), or fatal (5).

- **CTCAEv4 Grade 1**: mild; asymptomatic or mild symptoms; clinical or diagnostic observations only; intervention is not indicated.
- **CTCAEv4 Grade 2**: moderate; minimal, local, or noninvasive intervention is indicated; limiting to age-appropriate instrumental activities of daily living (ADL; instrumental ADL refers to preparing meals, shopping for groceries or clothes, using the telephone, managing money, etc).
- **CTCAEv4 Grade 3**: Severe or medically significant but not immediately life threatening; hospitalization or prolongation of hospitalization is indicated; disabling; limiting to self care ADL (self care ADL refers to bathing, dressing and undressing, feeding self, using the toilet, taking medications, and not bedridden).
- **CTCAEv4 Grade 4**: life-threatening consequences; urgent intervention is indicated.
- **CTCAEv4 Grade 5**: death due to an AE.

Attribution of AEs will be indicated as follows:

- **Definite**: The AE is clearly related to the study drug
- **Probably**: The AE is likely related to the study drug
- **Possible**: The AE may be related to the study drug
- **Unlikely**: The AE is doubtfully related to the study drug
- **Unrelated**: The AE is clearly NOT related to the study drug

Subjects must be carefully monitored for AEs. This monitoring also includes clinical laboratory tests. Adverse events should be assessed in terms of their seriousness, intensity, and relationship to the study drug, or other chemotherapy/treatment.

All adverse events should be treated appropriately. Such treatment may include changes in study drug treatment including possible interruption or discontinuation, starting or stopping concomitant treatments, changes in the frequency or nature of assessments, hospitalization, or any other medically required intervention. Once an adverse event is detected, it should be followed until its resolution, and assessment should be made at each visit (or more frequently, if necessary) of any changes in severity, the suspected relationship to the study drug, the interventions required to treat it, and the outcome.

Information about common side effects already known about radium 223 can be found in the Investigators' Brochure. This information should be included in the patient informed consent and should be discussed with the patient during the study as needed.

9.2 Serious Adverse Events

An AE is considered "serious" if in the opinion of the investigator it is one of the following outcomes:

- Fatal
- Life-threatening
- Constitutes a congenital anomaly or birth defect

- A medically significant condition (defined as an event that compromises subject safety or may require medical or surgical intervention to prevent one of the three outcomes above).
- Requires inpatient hospitalization or prolongation of existing hospitalization
- Results in persistent or significant incapacity or substantial disruption to conduct normal life functions.

Life-threatening (grade 4 or 5) SAEs and unknown reactions or unexpected events that occur in the course of any patient's treatment on study (from the time of registration) or within 30 days following cessation of treatment should be reported to the principal investigator at Duke University within 24 hours using the provided DCI SAE Report Form and theSAE Report Review Form. These documents should be sent to:

The initial report for each SAE or death should include at minimum the following information:

- protocol # and title
- patient initials, study identification number, sex, age
- date the event occurred
- description of the SAE
- dose level and cycle number at the time the SAE occurred
- description of the patient's condition
- indication whether the patient remains on study
- causality

Follow-up information including severity, action taken, concomitant medications, and outcome should be communicated to Duke as soon as possible.

Upon receipt of the Serious Adverse Event Reporting form by Duke, the PI will be notified and be required to complete the PI assessment of the DCI Safety SAE Report Review Form. The DCI safety desk will, in turn, report the SAE if at least possibly related to radium-223 to Bayer within 24 hours of knowledge of the SAE using the DCI SAE Report Form.

9.3 Adverse Events Summary

The following tables summarize the most common adverse events.

Adverse reaction:

System/ Organ Class Preferred Term	Radium-223 dichloride (n=600)		Placebo (n=301)	
	Grades 1-4 %	Grades 3-4 %	Grades 1-4 %	Grades 3-4 %
**Blood and lymphatic system disorders**				
Pancytopenia	2	1	0	0
**Gastrointestinal disorders**				
Nausea	36	2	35	2
Diarrhea	25	2	15	2
Vomiting	19	2	14	2
**General disorders and administration site conditions**				
Peripheral edema	13	2	10	1
**Renal and urinary disorders**				
Renal failure and impairment	3	1	1	1

Laboratory Abnormalities

**Hematologic**	**Xofigo (n=600)**		**Placebo (n=301)**	
**Laboratory**	**Grades 1-4**	**Grades 3-4**	**Grades 1-4**	**Grades 3-4**
**Abnormalities**	**%**	**%**	**%**	**%**
Anemia	93	6	88	6
Lymphocytopenia	72	20	53	7
Leukopenia	35	3	10	<1
Thrombocytopenia	31	3	22	<1
Neutropenia	18	2	5	<1

Fluid Status: Dehydration occurred in 3% of patients on Radium-223 dichloride and 1% of patients on placebo. Radium-223 dichloride increases adverse reactions such as diarrhea, nausea, and vomiting which may result in dehydration. Monitor patients' oral intake and fluid status carefully and promptly treat patients who display signs or symptoms of dehydration or hypovolemia.

Injection Site Reactions: Erythema, pain, and edema at the injection site were reported in 1% of patients on Radium-223 dichloride.

Secondary malignant neoplasms: No cases of radiation-induced cancer have been reported in reported in clinical trials with radium-223 dichloride in follow-up of up to three years. However, the radiation dose resulting from therapeutic exposure may result in higher incidence of cancer (e.g. sarcomas of the bone, or leukemia), mutations and a potential for development of hereditary defects.

Bone Marrow Suppression: In the randomized trial, 2% of patients on the Radium-223 dichloride arm experienced bone marrow failure or ongoing pancytopenia compared to no patients treated with placebo. There were two deaths due to bone marrow failure and for 7 of 13 patients treated with Radium-223 dichloride, bone marrow failure was ongoing at the time of death. Among the 13 patients who experienced bone marrow failure, 54% required blood transfusions. Four percent (4%) of patients on the Radium-223 dichloride arm and 2% on the placebo arm permanently discontinued therapy due to bone marrow suppression.

In the randomized trial, deaths related to vascular hemorrhage in association with myelosuppression were observed in 1% ofRadium-223 dichloride-treated patients compared to 0.3% of patients treated with placebo. The incidence of infection-related deaths (2%), serious infections (10%), and febrile neutropenia (<1%) were similar for patients treated with Radium-223 dichloride and placebo.

Myelosuppression; notably thrombocytopenia, neutropenia, pancytopenia, and leukopenia; has been reported in patients treated with Radium-223 dichloride. In the randomized trial, complete blood counts (CBCs) were obtained every 4 weeks prior to each dose and the nadir CBCs and times of recovery were not well characterized. In a separate single-dose phase 1 study of Radium-223 dichloride, neutrophil and platelet count nadirs occurred 2 to 3 weeks after Radium-223 dichloride administration at doses that were up to 1 to 5 times the recommended dose, and most patients recovered approximately 6 to 8 weeks after administration

Hematologic evaluation of patients must be performed at baseline and prior to every dose of Radium-223 dichloride. Before the first administration of Radium-223 dichloride, the absolute neutrophil count (ANC) should be ≥ 1.5 x 109/L, the platelet count ≥ 100 x 109/L and hemoglobin ≥ 9.0 g/dL. Before subsequent administrations of Radium-223 dichloride, the ANC should be ≥ 1 x 109/L and the platelet count ≥ 50 x 109/L. If there is no recovery to these values within 6 to 8 weeks after the last administration of Radium-223 dichloride, despite receiving supportive care, further treatment with Radium-223 dichloride should be discontinued. Patients with evidence of compromised bone marrow reserve should be monitored closely and

provided with supportive care measures when clinically indicated. Discontinue Radium-223 dichloride in patients who experience life-threatening complications despite supportive care for bone marrow failure.

Safety and efficacy of concomitant chemotherapy: the safety and efficacy of concomitant chemotherapy with Radium-223 dichloride have not been established. Outside of a clinical trial, concomitant use with chemotherapy is not recommended due to the potential for additive myelosuppression. If chemotherapy, other systemic radioisotopes or hemibody external radiotherapy are administered during the treatment period, Radium-223 dichloride should be discontinued.

9.4 Pregnancies

The investigator must report to Bayer any pregnancy occurring in a study subject's partner, during the subject's participation in this study. The report should be submitted within the same timelines as an SAE, although a pregnancy per se is not considered an SAE.

For the pregnancy of a study subject's partner, all efforts should be made to obtain information on course and outcome, subject to the partner's consent.

9.5 Safety Oversight Committee (SOC)

The Duke Cancer Institute SOC is responsible for annual data and safety monitoring of DUHS sponsor-investigator phase I and II, therapeutic interventional studies that do not have an independent Data Safety Monitoring Board (DSMB). The primary focus of the SOC is review of safety data, toxicities and new information that may affect subject safety or efficacy. Annual safety reviews includes but may not be limited to review of safety data, enrollment status, stopping rules if applicable, accrual, toxicities, reference literature, and interim analyses as provided by the sponsor-investigator. The SOC in concert with the DCI Monitoring Team (see Section 10.1 for Monitoring Team description) oversees the conduct of DUHS cancer-related, sponsor-investigator therapeutic intervention and prevention intervention studies that do not have an external monitoring plan, ensuring subject safety and that the protocol is conducted, recorded and reported in accordance with the protocol, standing operating procedures (SOPs), Good Clinical Practice (GCP), and applicable regulatory requirements.

10 QUALITY CONTROL AND QUALITY ASSURANCE

10.1 Monitoring

The Duke Cancer Institute (DCI) Monitoring Team will conduct monitoring visits to ensure subject safety and to ensure that the protocol is conducted, recorded, and reported in accordance with the protocol, standard operating procedures, good clinical practice, and applicable regulatory requirements. As specified in the DCI Data and Safety Monitoring Plan, the DCI Monitoring Team will conduct routine monitoring after the third subject is enrolled, followed by annual monitoring of 1 – 3 subjects until the study is closed to enrollment and subjects are no longer receiving study interventions that are more than minimal risk.

Additional monitoring may be prompted by findings from monitoring visits, unexpected frequency of serious and/or unexpected toxicities, or other concerns and may be initiated upon request of DUHS and DCI leadership, the DCI Cancer Protocol Committee, the Safety Oversight Committee (SOC), the sponsor, the Principal Investigator, or the IRB. All study documents must be made available upon request to the DCI Monitoring Team and other authorized regulatory authorities, including but not limited to the National Institute of Health, National Cancer Institute, and the FDA. Every reasonable effort will be made to maintain confidentiality during study monitoring.

10.2 Data Management and Processing

Study Documentation: Study documentation includes but is not limited to source documents, case report forms, monitoring logs, appointment schedules, study team correspondence with sponsors or regulatory bodies/committees, and regulatory documents that can be found in the DCI-mandated "Regulatory Binder", which

includes but is not limited to signed protocol and amendments, approved and signed informed consent forms, FDA Form 1572, CAP and CLIA laboratory certifications, and clinical supplies receipts and distribution records.

Source documents are original records that contain source data, which is all information in original records of clinical findings, observations, or other activities in a clinical trial necessary for the reconstruction and evaluation of the trial. Source documents include but are not limited to hospital records, clinical and office charts, laboratory notes, memoranda, subjects' diaries or evaluation checklists, pharmacy dispensing records, recorded data from automated instruments, copies or transcriptions certified after verification as being accurate copies, microfiches, photographic negatives, microfilm or magnetic media, x-rays, subject files, and records kept at the pharmacy, at the laboratories and at medico-technical departments involved in the clinical trial. When possible, the original record should be retained as the source document. However, a photocopy is acceptable provided that it is a clear, legible, and an exact duplication of the original document.

Case Report Forms (CRFs): Case report forms (CRFs) will be generated by Duke University for the collection of all study data. Investigators will be responsible for ensuring that the CRFs are kept up-to-date.

Source Documents: Study documentation includes all paper case report forms, data correction forms, source documents, monitoring logs and appointment schedules, sponsor-investigator correspondence and regulatory documents (e.g., signed protocol and amendments, Ethics or Institutional Review Committee correspondence and approval, approved and signed subject consent forms, Statement of Investigator form, and clinical supplies receipts and distribution records).

The investigator will prepare and maintain complete and accurate study documentation in compliance with Good Clinical Practice standards and applicable federal, state, and local laws, rules and regulations; and, for each subject participating in the study, promptly complete all original case report forms and such other reports as required by this protocol following completion or termination of the clinical study or as otherwise required pursuant to any agreement with the Sponsor-Investigator.

By signing the protocol, the investigator acknowledges that, within legal and regulatory restrictions and institutional and ethical considerations, study documentation will be promptly and fully disclosed to the Sponsor-Investigator/Regulatory Specialist by the investigator upon request and also shall be made available at the investigator's site upon request for inspection, copying, review and audit at reasonable times by representatives of Sponsor-Investigator or responsible government agencies as required by law. The investigator agrees to promptly take any reasonable steps that are requested by Sponsor-Investigator as a result of an audit to cure deficiencies in the study documentation and case report forms.

Record retention: The investigator will maintain adequate and accurate records to enable the conduct of the study to be fully documented and the study data to be subsequently verified. After study closure, the investigator will maintain all source documents, study-related documents, and CRFs. Because the length of time required for retaining records depends upon a number of regulatory and legal factors, documents should be stored until the investigator is notified that the documents may be destroyed. In this study, records are to be retained and securely stored for a minimum of 6 years after the completion of all study activities.

Research study assistants and research program coordinators: A research study assistant (RSA) at the coordinating center will be assigned to the study. A research program coordinator (RPC) will manage the study activities at each of the participating sites. The responsibilities of the RSA and RPCs include project compliance, data collection, data entry, data reporting, regulatory monitoring, problem resolution and prioritization, and coordination of the activities of the protocol team.

Study monitoring and quality assurance: The Sponsor-Investigator is responsible for monitoring the protocol to ensure that the investigation is conducted in accordance with the general investigational plan and all applicable regulatory requirements. In addition, the protocol will be monitored independently via the Duke Cancer Institute (DCI) Monitoring Team. The degree of monitoring is made at the time of initial Cancer Protocol Committee (CPC)

approval to commensurate with the type and level of intervention, phase, endpoints, degree of risk, size and complexity of the study. The minimum level of monitoring for interventional, therapeutic (agent or device) clinical studies is after the first 3 subjects have been enrolled, followed by annual monitoring of 1-3 subjects until closed to enrollment, subjects are no longer receiving study drug or other interventions that are more than minimal risk. Monitoring includes review of regulatory, eligibility, conduct, data quality and adverse event reporting.

Data Safety and Monitoring: Data for safety and severe adverse events will be monitored on an ongoing basis through monthly investigator and staff meetings.

In addition a recruitment and withdrawal summary will be discussed at these meetings. Withdraws will be broken down into those due to AEs and what they were. In terms of internal review, the Investigator will continuously monitor and tabulate adverse events. Appropriate reporting to the DUHS IRB will be made. If an unexpected frequency of Grade III or IV events occurs, depending on their nature, action appropriate to the nature and frequency of these adverse events will be taken. This may require a protocol amendment or closure of the study.

Protocol Amendments or Changes in Study Conduct: Any change or addition to this protocol requires a written protocol amendment that must be reviewed by Duke University before implementation.

Study Closure: Following completion of the studies, the PI will be responsible for ensuring the following activities:

- Data clarification and/or resolution
- Accounting, reconciliation, and destruction/return of used and unused study drugs
- Review of site study records for completeness
- Shipment of all remaining laboratory samples to the designated laboratories

11 STATISTICAL METHODS AND DATA ANALYSIS

Sample Size Justification:
The primary objective in this pilot study is to estimate the proportion of patients who will over-express alkaline phosphatase in their circulating tumor cells as a measure of osteomimicry. Secondary objectives will be to estimate other markers (RNA biomarkers of EMT and epithelial plasticity including alkaline phosphatase in their bone metastases, IHC biomarkers in metastases) measured from the baseline biopsy. Exploratory objectives will be to estimate the changes in markers obtained from the first and subsequent biopsies.

This is the first study to estimate the proportion of patients who will over-express ALP in the bone metastases and CTCs and as such the prevalence of patients who are expected to over-express ALP in the bone metastases is unknown. It is though anticipated that at least 50% will over-express ALP in their CTCs and/or bone metastases. CTC ALP positivity will be determined by the presence of at least one CTC expressing ALP, with a CTC defined as a CD45 negative, nucleated, intact cell. CTCs will also be defined by their EpCAM and cytokeratin expression, and ALP expression described within the EpCAM or CK negative fraction of CTCs. With 20 patients enrolled in the study, the proportion of patients who will over-express alkaline phosphatase in the bone metastases (defined as any over-expression) can be estimated with a standard error of no greater than 11.2 percentage points.

Data Analysis
This is a pilot correlative science study. For the primary objective, the proportion of patients who will over-express ALP in their CTCs and bone metastases will be estimated and the exact 95% confidence intervals based on the binomial distribution will be computed. The expression levels of the biomarkers will be summarized using descriptive statistics (means, SE, median, and inter-quartile ranges) at baseline and over time, with 95% confidence intervals around these estimates. Scatterplots and waterplots for changes from baseline for all markers at the indicated time point will be used. The Pearson's correlation coefficient and 95% confidence interval will be calculated for each paired markers. The Kaplan-Meier product limit method will be used to estimate the PFS and

OS distributions. While it is expected that this pilot study with a small sample size to be exploratory, the estimates will be useful in the planning of larger trials.

12 ADMINISTRATIVE AND ETHICAL CONSIDERATIONS

12.1 Regulatory and Ethical Compliance

This protocol was designed and will be conducted and reported in accordance with the International Conference on Harmonization (ICH) Harmonized Tripartite Guidelines for Good Clinical Practice, the Declaration of Helsinki, and applicable federal, state, and local regulations.

12.2 DUHS Institutional Review Board and DCI Cancer Protocol Committee

The protocol, informed consent form, advertising material, and additional protocol-related documents must be submitted to the DUHS Institutional Review Board (IRB) and DCI Cancer Protocol Committee (CPC) for review. The study may be initiated only after the Principal Investigator has received written and dated approval from the CPC and IRB.

The Principal Investigator must submit and obtain approval from the IRB for all subsequent protocol amendments and changes to the informed consent form. The CPC should be informed about any protocol amendments that potentially affect research design or data analysis (i.e. amendments affecting subject population, inclusion/exclusion criteria, agent administration, statistical analysis, etc.).

The Principal Investigator must obtain protocol re-approval from the IRB within 1 year of the most recent IRB approval. The Principal Investigator must also obtain protocol re-approval from the CPC within 1 year of the most recent IRB approval, for as long as the protocol remains open to subject enrollment.

12.3 Informed Consent

The informed consent form must be written in a manner that is understandable to the subject population. Prior to its use, the informed consent form must be approved by the IRB.

The Principal Investigator or authorized key personnel will discuss with the potential subject the purpose of the research, methods, potential risks and benefits, subject concerns, and other study-related matters. This discussion will occur in a location that ensures subject privacy and in a manner that minimizes the possibility of coercion. Appropriate accommodations will be made available for potential subjects who cannot read or understand English or are visually impaired. Potential subjects will have the opportunity to contact the Principal investigator or authorized key personnel with questions, and will be given as much time as needed to make an informed decision about participation in the study.

Before conducting any study-specific procedures, the Principal Investigator must obtain written informed consent from the subject or a legally acceptable representative. The original informed consent form will be stored with the subject's study records, and a copy of the informed consent form will be provided to the subject. The Principal Investigator is responsible for asking the subject whether the subject wishes to notify his/her primary care physician about participation in the study. If the subject agrees to such notification, the Principal Investigator will inform the subject's primary care physician about the subject's participation in the clinical study.

12.4 Privacy, Confidentiality, and Data Storage

The Principal Investigator will ensure that subject privacy and confidentiality of the subject's data will be maintained. Research Data Security Plans (RDSPs) will be approved by the appropriate institutional Site Based Research group.

To protect privacy, every reasonable effort will be made to prevent undue access to subjects during the course of the study. Prospective participants will be consented in an exam room where it is just the research staff, the

patient and his family, if desired. For all future visits, interactions with research staff (study doctor and study coordinators) regarding research activities will take place in a private exam room. All research related interactions with the participant will be conducted by qualified research staff who are directly involved in the conduct of the research study.

To protect confidentiality, subject files in paper format will be stored in secure cabinets under lock and key accessible only by the research staff. Electronic records of subject data will be maintained using a dedicated database, which is housed in an encrypted and password-protected DCI file server. Access to electronic databases will be limited to delegated personnel. The security and viability of the IT infrastructure will be managed by the DCI and/or Duke Medicine.

Upon completion of the study, research records will be archived and handled per DUHS HRPP policy. Subject names or identifiers will not be used in reports, presentations at scientific meetings, or publications in scientific journals.

12.5 Data and Safety Monitoring

Data and Safety Monitoring will be performed in accordance with the DCI Data and Safety Monitoring Plan. For a more detailed description of the DSMP for this protocol, refer to Section 10.1.

12.6 Protocol Amendments

All protocol amendments must be initiated by the Principal Investigator and approved by the IRB prior to implementation. IRB approval is not required for protocol changes that occur to protect the safety of a subject from an immediate hazard. However, the Principal Investigator must inform the IRB and all other applicable regulatory agencies of such action immediately.

Though not yet required, the CPC should be informed about any protocol amendments that potentially affect research design or data analysis (i.e. amendments affecting subject population, inclusion/exclusion criteria, agent administration, etc.).

12.7 Records Retention

The Principal Investigator will maintain study-related records for at least six years after study completion.

12.8 Publication policy

Bayer recognizes the right of the investigator to publish results upon completion of the study. However, the investigator must send a draft manuscript of the publication or abstract to Bayer at least thirty days in advance of submission in order to obtain approval prior to submission of the final version for publication or congress presentation. This will be reviewed promptly and approval will not be withheld unreasonably. In case of a difference of opinion between Bayer and the investigator(s), the contents of the publication will be discussed in order to find a solution which satisfies both parties. All relevant aspects regarding data reporting and publication will be part of the contract between Bayer and the investigator/institution

The Principal Investigator should ensure that the information regarding the study be publicly available on the internet at www.clinicaltrials.gov.

Bayer recommends inclusion of the new NIST standard in abstracts/ publications submitted OR pending publication January 2016 onwards from IIRs and other non- Bayer supported abstracts/publications for consistency. Investigators may choose to add a footnote to the publication or within the body of the publication include the new NIST standard.

12.9 Conflict of Interest

The Principal Investigator and Sub-Investigators must comply with applicable federal, state, and local regulations regarding reporting and disclosure of conflict of interest. Conflicts of interest may arise from situations in which

financial or other personal considerations have the potential to compromise or bias professional judgment and objectivity. Conflicts of interest include but are not limited to royalty or consulting fees, speaking honoraria, advisory board appointments, publicly-traded or privately-held equities, stock options, intellectual property, and gifts.

The Duke University School of Medicine's Research Integrity Office (RIO) reviews and manages research-related conflicts of interest. The Principal Investigator and Sub-Investigators must report conflicts of interest annually and within 10 days of a change in status, and when applicable, must have a documented management plan that is developed in conjunction with the Duke RIO and approved by the IRB/IEC.

13 References

Armstrong AJ, Garrett-Mayer ES, et al. A contemporary prognostic nomogram for men with hormone-refractory metastatic prostate cancer: a TAX327 study analysis. Clin Cancer Res. 2007;13:6396–403.

Armstrong AJ, et al. Circulating tumor cells from patients with advanced prostate and breast cancer display both epithelial and mesenchymal markers. Mol Cancer Res. 2011;9:997-1007.

Cessna JT, Zimmerman BE. Standardization of radium-223 by liquid scintillation counting. Appl. Radiat. Isot. 2010;68:1523-8.

Cheetham PJ, et al. Alpha particles as radiopharmaceuticals in the treatment of bone metastases: mechanism of action of radium-223 chloride (Alpharadin) and radiation protection. Oncology. 2012;26(4):330-7, 341.

Chu K et al. Cadherin-11 promotes the metastasis of prostate cancer cells to bone. Mol Cancer Res. 2008, 6(8):1259-67.

Clarke JM, Armstrong AJ. Novel Therapies for the Treatment of Advanced Prostate Cancer. Curr Treat Options Oncol. 2013;14(1):109-26.

Eisenhauer, E.A., et al. New response evaluation criteria in solid tumours: revised RECIST guideline (version 1.1). Eur J Cancer, 2009. 45(2): 228-47.

Green DA, et al. Predictive tools for prostate cancer staging, treatment response and outcomes. Arch Esp Urol.2012;65(9):787-807.

Halabi S, Small EJ, Kantoff PW, et al. Prognostic model for predicting survival in men with hormone-refractory metastatic prostate cancer. J Clin Oncol. 2003;21:1232–7.

Halabi S, Ou S, Vogelzang NJ, et al. The prognostic value of change in hemoglobin (HGB), LDH and PSA levels at 3 months from baseline in men with castrate recurrent prostate cancer [abstract]. At: American Society of Clinical Oncology Prostate Cancer Symposium; February 22-24, 2007; Orlando, FL, USA.

Harrison MR, Wong TZ. Radium-223 chloride: a potential new treatment for castration-resistant prostate cancer patients with metastatic bone diseas. Cancer Manag Res. 2013; 5: 1–14.

Henriksen G, Breistøl K, Bruland OS, Fodstad O, Larsen RH. Significant antitumor effect from bone-seeking, alpha-particle-emitting (223)Ra demonstrated in an experimental skeletal metastases model. Cancer Res. 2002;62(11):3120-3125.

Henriksen G, Fisher DR, Roeske JC, Bruland OS, Larsen RH. Targeting of osseous sites with alpha-emitting 223Ra: comparison with the beta-emitter 89Sr in mice. J Nucl Med. 2003;44(2):252-259.

Jemal, A., et al. Cancer statistics, 2009. CA Cancer J Clin. 2009.;59(4):225-49.

Koeneman KS, et al. Osteomimetic properties of prostate cancer cells: a hypothesis supporting the predilection of prostate cancer metastasis and growth in the bone environment.Prostate. 1999;39(4):246-61.

Knerr K, et al. Bone metastases: osteoblasts affect growth adhesion regulations in prostate tumor cells and provoke osteominicry. Int J Cancer. 2004;111:152–9.

Lin D. et al. Bone metastatic LNCaP-derivative C4-2B prostate cancer cell line mineralizes in vitro. Prostate 47: 212-221, 2001.

Miller CE et al. Radium retention in mice after single intravenous injection. Radiat Res. 1965; 26:269-286.

Nilsson S, et al. A randomized, dose–response, multicenter phase II study of radium-223 chloride for the palliation of painful bone metastases in patients with castration-resistant prostate cancer. Eur J Cancer. 2012;48(5):678-86

Nilsson S, Franzen L, Parker C, et al. Two-year survival follow-up of the randomized, double-blind, placebo-controlled phase II study of radium-223 chloride in patients with castration-resistant prostate cancer and bone metastases. Clin Genitourin Cancer. 2013;11(1):20-26.

Parker C. et al. ASCO 2012. Overall Survival Benefit and Safety Profile of Radium-223 Chloride (Alpharadin), A First-in-Class Alpha-Pharmaceutical: Results from a Phase III Randomized Trial (ALSYMPCA) in Patients With Castration-Resistant Prostate Cancer (CRPC) With Bone Metastases.

Parker C. et al. ICPU 2013. Quality of life (QOL) and updated survival and safety data of radium-223 dichloride in patients with castration-resistant prostate cancer (CRPC) with bone metastases from the phase 3 ALSYMPCA study.

Parker C, et al. Alpha emitter radium-223 and survival in metastatic prostate cancer. N Eng J Med. 2013 Jul 18;369(3):213-23.

Scher, H.I., et al., Design and end points of clinical trials for patients with progressive prostate cancer and castrate levels of testosterone: recommendations of the Prostate Cancer Clinical Trials Working Group. J Clin Oncol, 2008. 26(7): 1148-59.

Smaletz O, et al. Nomogram for overall survival of patients with progressive metastatic prostate cancer after castration. Clin Oncol. 2002;20(19):3972-82.

Sonpavde G, Pond GR, Berry WR, et al. Serum alkaline phosphatase changes predict survival dependent of PSA changes in men with castration-resistant prostate cancer and bone metastasis receiving chemotherapy. Urol Oncol. 2010; 5: 607-613.

Vaidyanathan G, et al. Applications of 211At and 223Ra in Targeted Alpha-Particle Radiotherapy. Current Radiopharmaceuticals. 2011, 4, 283-294.

Zhau HE et al. Human Prostate Cancer Harbors the Stem Cell Properties of Bone Marrow Mesenchymal Stem Cells. Clin Cancer Res. 2011;17(8):2159-69.

Zimmerman BE, Bergeron DE, Cessna JT, Fitzgerald R. Revision of the NIST Standard for 223Ra: New Measurements and Review of 2008 data.J Res Natl InstStand Technol. 2015;120:37-57.

14 APPENDICES

14.1 Performance Status

ECOG Performance Status Scale		Karnofsky Performance Scale	
**Grade**	**Description**	**%**	**Description**
0	Normal activity. Fully active, able to continue all pre-disease performance without restriction.	100	Normal, no complaints, no evidence of disease
		90	Able to carry on normal activity, minor signs or symptoms of disease
1	Symptoms, but ambulatory. Restricted in physically strenuous activity but ambulatory and able to carry out work of a light or sedentary nature (e.g., light housework, office work).	80	Normal activity with effort, some signs or symptoms of disease
		70	Cares for self, unable to carry on normal activity or to do active work
2	In bed < 50% of the time. Ambulatory and capable of all self-care but unable to carry out any work activities. Up and about more than 50% of waking hours.	60	Requires occasional assistance but is able to care for most needs
		50	Requires considerable assistance and frequent medical care
3	In bed > 50% of the time. Capable of only limited self-care, confined to bed or chair > 50% of waking hours.	40	Disabled, requires special care and assistance
		30	Severely disabled, hospitalization indicated. Death not imminent.
4	100% bedridden. Completely disabled cannot carry on any self-care, totally confined to bed or chair.	20	Very sick, hospitalization indicated. Death not imminent.
		10	Moribund, fatal processes progressing rapidly
5	Dead	0	Dead

14.2 NCI COMMON TOXICITY CRITERIA

NCI Common Terminology Criteria for Adverse Events (CTCAE) version 4.0 can be found here: http://evs.nci.nih.gov/ftp1/CTCAE/About.html

14.3 CRITERIA FOR DISEASE PROGRESSION

PROSTATE CANCER WORKING GROUP 2 (PCWG2) GUIDELINES

Variable	Assessments	Progression
**Soft-tissue lesions**	Use RECIST 1.1 criteria, except:<br>• Only report changes in lymph nodes that were ~2 cm in diameter at baseline<br>• Record changes in nodal and visceral soft tissue sites separately<br>• Record complete elimination of disease at any site separately<br>• Confirm favorable change with next planned scan 8 weeks later	Use RECIST 1.1 criteria for progression, except:<br>• Progression at first assessment must be confirmed by a second scan 6-8 weeks later<br>(For some treatments, a lesion may increase in size before it decreases)
**Bone**	Record as either *new lesions* or *no new lesions*<br><br>If no new lesions, continue therapy<br><br>If new lesions: continue therapy but perform a confirmatory scan 8 weeks later.<br><br>If confirmatory scan has no new lesions, continue therapy. If there are *additional* new lesions, this is progression.	The appearance of 2 new lesions<br>AND<br>A confirmatory scan 8 weeks later showing at least 2 *additional* new lesions<br><br>The date of progression is the date of the first scan that shows the change
**Symptoms**	Consider independently of other outcome measures<br>One or more of the following criteria indicates disease progression:<br>• Need for new systemic therapy<br>• Need for palliative radiation therapy<br>• Development of a new skeletal-related event	
